# Augmented balancing weights as linear regression

**DOI:** 10.1093/jrsssb/qkaf019

**Published:** 2025-04-24

**Authors:** David Bruns-Smith, Oliver Dukes, Avi Feller, Elizabeth L Ogburn

**Affiliations:** Graduate School of Business, Stanford University, Stanford, CA, USA; Department of Mathematics, Computer Science and Statistics, Ghent University, Ghent, Belgium; Goldman School of Public Policy, Department of Statistics, University of California, Berkeley, CA, USA; Department of Biostatistics, Bloomberg School of Public Health, Johns Hopkins University, Baltimore, MD, USA

**Keywords:** balancing weights, debiased machine learning, doubly robust estimation, undersmoothing

## Abstract

We provide a novel characterization of augmented balancing weights, also known as automatic debiased machine learning. These popular *doubly robust* estimators combine outcome modelling with balancing weights—weights that achieve covariate balance directly instead of estimating and inverting the propensity score. When the outcome and weighting models are both linear in some (possibly infinite) basis, we show that the augmented estimator is equivalent to a single linear model with coefficients that combine those of the original outcome model with those from unpenalized ordinary least-squares (OLS). Under certain choices of regularization parameters, the augmented estimator in fact collapses to the OLS estimator alone. We then extend these results to specific outcome and weighting models. We first show that the augmented estimator that uses (kernel) ridge regression for both outcome and weighting models is equivalent to a single, undersmoothed (kernel) ridge regression—implying a novel analysis of undersmoothing. When the weighting model is instead lasso-penalized, we demonstrate a familiar ‘double selection’ property. Our framework opens the black box on this increasingly popular class of estimators, bridges the gap between existing results on the semiparametric efficiency of undersmoothed and doubly robust estimators, and provides new insights into the performance of augmented balancing weights.

## 1 Introduction

Combining outcome modelling and weighting, as in augmented inverse propensity score weighting (AIPW) and other doubly robust (DR) or double machine learning (DML) estimators, is a core strategy for estimating causal effects using observational data. A growing body of the literature finds weights by solving a ‘balancing weights’ optimization problem to estimate weights directly, rather than by first estimating the propensity score and then inverting. DR versions of these estimators are referred to by a number of terms, including *augmented balancing weights* (Athey et al., 2018; Hirshberg & Wager, 2021), *automatic debiased machine learning* (AutoDML; Chernozhukov et al., 2022a), and *generalized regression estimators* (GREG; Deville & Särndal, 1992); see Ben-Michael, Feller, Hirshberg, et al. (2021) for a review. Moreover, this strategy has been applied to a wide range of linear estimands via the Riesz representation theorem (e.g. Chernozhukov et al., 2022b; Hirshberg & Wager, 2021). In this paper, we consider augmented balancing weights in which the estimators for both the outcome model and the balancing weights are based on penalized linear regressions in some possibly infinite basis; in addition to all high-dimensional linear models, this broad class includes popular nonparametric models such as kernel regression and certain forms of random forests and neural networks.

We first show that, somewhat surprisingly, augmenting any regularized linear outcome regression (the ‘base learner’) with linear balancing weights is numerically equivalent to a single linear outcome regression applied to the target covariate profile. The resulting coefficients are an affine (and often convex) combination of the base learner model coefficients and unregularized OLS coefficients; the hyperparameter for the balancing weights estimator directly controls the regularization path defining the affine combination. In the extreme case where the weighting hyperparameter is set to zero—which we show can occur in practice—the entire procedure is equivalent to estimating a single, unregularized OLS regression.

We specialize these results to ridge and lasso regularization ($\ell_{2}$ and $\ell_{\infty }$ balancing, respectively) and show that augmenting an outcome regression estimator with balancing weights generally corresponds to a form of *undersmoothing*. Most notably, we show that an augmented balancing weight estimator that uses (kernel) ridge regression for both outcome and weighting models—which we refer to as ‘double ridge’—collapses to a single, undersmoothed (kernel) ridge regression estimator.

We leverage these results to prove novel *statistical* results for double ridge estimators and to make progress towards practical hyperparameter tuning, which remains an open problem in this area. We first make explicit the connection between asymptotic results for double kernel ridge estimators (e.g. Singh, 2024) and prior results on optimal undersmoothing for a single kernel ridge outcome model (e.g. Mou et al., 2023), showing that the latter is also semiparametrically efficient. This generalizes the argument in J. Robins et al. (2007) that ‘OLS is DR’ to a much broader class of penalized parametric and nonparametric regression estimators. As a complementary analysis, we next adapt existing finite sample error analysis results for single ridge regression (Dobriban & Wager, 2018) to derive the finite-sample-exact bias and variance of double ridge estimators. Using these expressions, we can compute oracle hyperparameters for any given data-generating process (DGP).

Finally, we illustrate our results with several numerical examples. We first explore hyperparameter tuning for double ridge regression in an extensive simulation study on 36 DGPs, and compare three practical methods to the optimal hyperparameter computed using our finite sample analysis. Both asymptotic theory and our simulation results suggest equating the hyperparameters for the outcome and weighting models. We further caution against the naive application of hyperparameter tuning based solely on cross-validating the weighting model, forms of which have been suggested previously. This approach can lead to setting the weighting hyperparameter to exactly zero—and therefore recovering standard OLS—even in scenarios where OLS is far from optimal. We emphasize this point by applying our results to the canonical (LaLonde, 1986) study, highlighting that researchers can inadvertently recover OLS in practice.

Broadly, our results provide important insights into the nexus of causal inference and machine learning. First, these results open the black box on the growing number of methods based on augmented balancing weights and AutoDML—methods that can sometimes be difficult to taxonomize or understand. We show that, under linearity, these estimators all share an underlying and very simple structure. Our results further highlight that estimation choices for augmented balancing weights can lead to potentially unexpected behaviour. At a high level, as causal inference moves towards incorporating machine learning and automation, our work highlights how the traditional lines between weighting and regression-based approaches are becoming increasingly blurred.

Second, our results connect two approaches to ‘automate’ semiparametric causal inference. AutoDML and related methods exploit the fact that we can estimate a Riesz representer without a closed-form expression for a wide class of functionals. The estimated Riesz representer then augments a base learner by bias correcting a plug-in estimator of the functional. Older approaches, such as undersmoothing (Goldstein & Messer, 1992; Newey et al., 1998), twicing kernels (Newey et al., 2004), and sieve estimation (Newey, 1994; Shen, 1997), avoid estimation of the Riesz representer altogether, instead tuning the base learner regression fit such that an additional bias correction is not required. Achieving this optimal tuning in practice has long been a hurdle for the implementation of these methods. Subject to certain conditions, both approaches can yield estimators that are asymptotically efficient. We show that if all required tuning parameters are defined in terms of an $\ell_{2}$-norm constraint, then these approaches can be numerically identical even in finite samples. We use these equivalences to make progress toward practical hyperparameter selection and find promising directions for new theoretical analysis.

In Section 2, we introduce the problem set-up, identification assumptions, and common estimation methods; we also review balancing weights and previous results linking balancing weights to outcome regression models. In Section 3, we present our new numerical results, and in Sections 4 and 5, we cache out the implications for $\ell_{2}$ and $\ell_{\infty }$ balancing weights specifically. Building on our numerical results, Section 6 explores both asymptotic and finite sample statistical results for kernel ridge regression. Section 7 illustrates our results with a simulation study and application to canonical datasets. Section 8 offers some other directions for future research. The online supplementary material, appendix includes extensive additional technical discussion and extensions.

### 1.1 Related work

#### 1.1.1 Balancing weights and AutoDML

With deep roots in survey calibration methods and the *generalized regression estimator* (GREG; see Deville & Särndal, 1992; Gao et al., 2023; Lumley et al., 2011), a large and growing causal inference literature uses balancing weights estimation in place of traditional inverse propensity score weighting (IPW). Ben-Michael, Feller, Hirshberg, et al. (2021) provide a recent review; we discuss specific examples at length in Section 2.3. This approach typically balances features of the covariate distributions in the different treatment groups, with the aim of minimizing the maximal design-conditional mean squared error of the treatment effect estimator. Of particular interest, here are augmented balancing weights estimators that combine balancing weights with outcome regression (see, for example, Athey et al. (2018), Hirshberg and Wager (2021), and Ben-Michael, Feller, Rothstein (2021)).

A parallel literature in econometrics instead focuses on the so-called *automatic* estimation of the Riesz representer, of which IPW is a special case, where ‘automatic’ refers to the fact that we can estimate the Riesz representer without obtaining a closed-form expression. Estimating the Riesz representer directly, under the assumption that it is linear in some basis, dates back at least to J. Robins et al. (2008); see also J. Robins et al. (2007). The corresponding augmented estimation framework has more recently come to be known as AutoDML; see, among others, Chernozhukov, Escanciano, et al. (2022), Chernozhukov, Newey, Quintas-Martinez, et al. (2022), and Chernozhukov et al. (2022a, 2022b). This approach has also been applied in a range of settings, including to corrupted data (Agarwal & Singh, 2021), to dynamic treatment regimes (Chernozhukov, Newey, Singh, et al., 2022), and to address noncompliance (Singh & Sun, 2022). As we discuss in online supplementary material, Appendix C.3, the AutoDML approach nearly always employs cross-fitting and is typically motivated by asymptotic properties rather than achieving minimax design-conditional mean squared error.

#### 1.1.2 Numerical equivalences for balancing weights

Many seminal papers highlight connections between weighting approaches, such as balancing weights and IPW, and outcome modelling; see Bruns-Smith and Feller (2022) for discussion. Most relevant are a series of papers that show numerical equivalences between linear regression and (exact) balancing weights, especially (Chattopadhyay & Zubizarreta, 2023; Kline, 2011; J. Robins et al., 2007), and between kernel ridge regression and forms of kernel weighting (Hirshberg et al., 2019; Kallus, 2020). We discuss these equivalences at length in online supplementary material, Appendix A.5. Finally, as we discuss in online supplementary material, Appendix D, there are close connections between balancing weights and Empirical Likelihood (Hellerstein & Imbens, 1999; Newey & Smith, 2004).

## 2 Problem set-up and background

### 2.1 Set-up and motivation

The core results in our paper are numeric equivalences for existing estimation procedures, and as such these results hold absent any causal assumptions or statistical model. Nonetheless, a primary motivation for this work is the task of estimating unobserved counterfactual means in causal inference, as well as estimating the broad class of linear functionals described in Chernozhukov, Newey, et al. (2018). We briefly review the corresponding set-up, emphasizing that this is purely for interpretation.

#### 2.1.1 Example: estimating counterfactual means

Let X,Y,Z be random variables defined on X,R,Z with joint probability distribution *p*. To begin, consider the example of a binary treatment, Z={0,1} and covariates *X*. Define potential or counterfactual outcomes Y(1) and Y(0) under assignment to treatment and control, respectively. Under SUTVA (Rubin, 1980), we observe outcomes Y=ZY(1)+(1−Z)Y(0). To estimate the average treatment effect, E[Y(1)−Y(0)], we first estimate the means of the partially observed potential outcomes. We initially focus on estimating E[Y(1)]; a symmetric argument holds for E[Y(0)].

Let m(x,z):=E[Y∣X=x,Z=z] be the *outcome model*, e(x):=P[Z=1∣X=x] be the *propensity score*, and α(x,z)=z/e(x) be the *inverse propensity score weights* (IPW). Under the additional assumptions of *conditional ignorability*, Y(1)⊥⊥Z∣X, and *overlap*, $E[\alpha (X,Z{)}^{2}]<\infty$, E[Y(1)] is identified by E[m(X,1)], a linear functional of the observed data distribution.

There are three broad strategies for estimating E[Y(1)]. First, the identifying functional above suggests estimating the outcome model, m(x,1) among those units with Z=1, and plugging this into the *regression functional*, E[m(X,1)]. Second, the equality E[m(X,1)]=E[Z/e(X)Y]=E[α(X,Z)Y] suggests estimating the inverse propensity score weights, α(x,z)=z/e(x), and plugging these into the *weighting functional*. Finally, we can combine these two via the *DR functional* (J. M. Robins et al., 1994):


E[m(X,1)+α(X,Z)(Y−m(X,1))].

This functional has the attractive property of being equal to E[m(X,1)] even if either one of *α* or *m* is replaced with an arbitrary function of *X* and *Z*, hence the term ‘DR’. DR estimators have been studied extensively in semiparametric theory; note that m(X,1)+α(X,Z)(Y−m(X,Z))−ψ(m) coincides with the efficient influence function for ψ(m) under a nonparametric model. See Chernozhukov, Chetverikov, et al. (2018) and Kennedy (2022) for overviews of the active literature in causal inference and machine learning focused on estimating versions of this functional.

#### 2.1.2 General class of functionals via the Riesz representer

Our results apply well beyond the example above. In particular, they apply to any functional of the form


(1) ψ(m)=E[h(X,Z,m)],

where *Z* a random variable with support Z; and *h* is a real-valued, mean-squared continuous linear functional of *m* (Chernozhukov, Newey, et al., 2018; Chernozhukov et al., 2022a; Hirshberg & Wager, 2021). Following Chernozhukov et al. (2022a, 2022b), we can generalize the weighting functional to this general class of estimands via the *Riesz representer*, which is a function $\alpha (X,Z)\in L_{2}(p)$ such that, for all square-integrable functions $f\in L_{2}(p)$:


(2) E[h(X,Z,f)]=E[α(X,Z)f(X,Z)].

As in the counterfactual mean example, we can identify the more general target functional in (2) via the outcome regression functional in (1), via the Riesz representer functional in (2) with f=m, or via the doubly robust functional


(3) E[h(X,Z,m)+α(X,Z)(Y−m(X,Z))].

Estimators of this DR functional are *augmented* in the sense that they augment the ‘plug-in’, ‘outcome regression’, or ‘base learner’ estimator of E[h(X,Z,m)] with appropriately weighted residuals; or, equivalently, augment the weighting estimator with an appropriate outcome regression. This is the class of estimators to which our results apply. As before, h(X,Z,m)+α(X,Z)(Y−m(X,Z))−ψ(m) coincides with the efficient influence function for ψ(m) under a nonparametric model. In future work, we will explore whether we can extend our results to a different class of functionals that admit DR functional forms, first introduced by J. Robins et al. (2008), and to the superset of such functionals characterized by Rotnitzky et al. (2021).

### 2.2 Balancing weights: background and general form

The core idea behind balancing weights is to estimate the Riesz representer directly—rather than via an analytic functional form (e.g. by estimating the propensity score and inverting it). As a result, balancing weights do not require a known analytic form for the Riesz representer (Chernozhukov et al., 2022b), are often more stable (Zubizarreta, 2015), and can offer improved control of finite sample covariate imbalance (Zhao, 2019). We briefly describe two primary motivations for this approach.

First, a central property of the Riesz representer is that the corresponding weights, w(X,Z)=α(X,Z), are the unique weights that satisfy the *population balance property* property in Equation (2) for all square-integrable functions $f\in L_{2}(p)$. For our target estimand ψ(m), we only need to satisfy the condition in Equation (2) for the special case of f=m. If we are willing to assume that *m* lies in a model class $F\subset L_{2}(p)$, then it suffices to balance functions in that class. This is achieved by minimizing the imbalance over F:


(4) $${\text{Imbalance}}_{F}(w)\;:=\underset{\;f\in F}{sup}\;\{E[w(X,Z)f(X,Z)]-E[h(X,Z,f)]\}.$$

As we discuss next, balancing weights minimize a (penalized) sample analogue of Equation (4).

Alternatively, Chernozhukov et al. (2022a) consider finding weights *f* that minimize the mean-squared error for α(X,Z):


(5) $$\min_{\;f\in F}\left\{E\left[{\left(\;f(X,Z)-\alpha (X,Z)\right)}^{2}\right]\right\}.$$

Automatic estimation of the Riesz representer, also known as *Riesz regression* (Chernozhukov et al., 2024), minimizes a sample analogue of Equation (5). When F is convex, then up to choice of hyperparameters (see (6)), the solutions to Equations (4) and (5) are equivalent.

### 2.3 Linear balancing weights

In this paper, we consider the special case in which outcome models are linear in some basis expansion of *X* and *Z*. This is an extremely broad class that encompasses linear and polynomial models of arbitrary functions of *X* and *Z* and with dimension possibly larger than the sample size, as well as nonparametric models such as reproducing kernel Hilbert spaces (RKHSs; Gretton et al., 2012), the Highly adaptive Lasso (Benkeser & Van Der Laan, 2016), the neural tangent kernel space of infinite-width neural networks (Jacot et al., 2018), and ‘honest’ random forests (Agarwal et al., 2022). However, this class excludes models for *m* that are fundamentally nonlinear in their parameters, like general neural networks or generalized linear models with a nonlinear link function. We sketch a preliminary extension of our results to arbitrary nonlinear balancing weights in online supplementary material, Appendix D.

Under linearity, the imbalance over all f∈F has a simple closed form. Because our results concern numeric equivalences, we will focus on the finite sample version of the linear balancing weights problem. Let $F=\{f(x,z)=\theta^{⊤}\phi (x,z)\;:\;\|\theta \|\leq 1\}$ where ‖⋅‖ can be any norm on $R^{d}$. The general set-up constrains ‖θ‖≤r; we set r=1 without loss of generality, which simplifies exposition below. Let $\|\cdot {\|}_{*}$ be the *dual norm* of ‖⋅‖; that is, $\|v{\|}_{*}\;:={\text{sup}}_{\|u\|\leq 1}u^{⊤}v.$ Many common vector norms have familiar, closed-form, dual norms, e.g. the dual norm of the $\ell_{2}$-norm is the $\ell_{2}$-norm; and the dual norm of the $\ell_{1}$-norm is the $\ell_{\infty }$-norm. Let $X_{p},Y_{p},Z_{p}$ be *n* i.i.d. samples from the distribution *p* of the observed data. Define the feature map $\phi \;:\;X\times Z\to R^{d}$ and let $\phi_{j}\;:\;X\times Z\to R$ denote the mapping for the *j*th feature. Define $\Phi_{p}\;:=\phi (X_{p},Z_{p})$ and let $\Phi_{q}\;:=h(X_{p},Z_{p},\phi )$ denote the *target features*. We will write $\hat{E}$ for sample averages; define ${\bar{\Phi }}_{p}\;:=\hat{E}[\Phi_{p}]$ and ${\bar{\Phi }}_{q}\;:=\hat{E}[\Phi_{q}]$. For exposition, we assume that d<n and that $\Phi_{p}$ has rank *d*. We emphasize that this is not necessary for our results—one can replace $R^{d}$ with an infinite-dimensional Hilbert space H and relax the rank restriction. See online supplementary material, Appendix B for a formal presentation of the high-dimensional (d>n) setting.

In what follows we write *w* for the 1×n vector $w(\Phi_{p})$, to highlight the fact that we will estimate *w* directly rather than as an explicit function of *X* or $\Phi_{p}$. Using the derivation above, we can directly calculate the finite sample imbalance as:


$$\hat{{\text{Imbalance}}_{F}}(w)={\left\|\frac{1}{n}w\Phi_{p}-{\bar{\Phi }}_{q}\right\|}_{*}.$$

Now we can write the penalized sample analogue of balancing weights optimization problem in (4) equivalently as either:


$$\begin{array}{rl} \text{Penalized form}:\; & \min_{w\in R^{n}}\left\{{\left\|\frac{1}{n}w\Phi_{p}-{\bar{\Phi }}_{q}\right\|}_{*}^{2}+\;\delta_{1}\|w{\|}_{2}^{2}\right\}\\ \text{Constrained form}:\; & \min_{w\in R^{n}}\|w{\|}_{2}^{2}\\ & \text{such that }{\left\|\frac{1}{n}w\Phi_{p}-{\bar{\Phi }}_{q}\right\|}_{*}\leq \delta_{2}. \end{array}$$

Furthermore, we can write the equivalent problem in (5) as:


(6) $$\text{Riesz regression form}\;:\;\;\min_{\theta \in R^{d}}\left\{\frac{1}{n}\theta^{⊤}(\Phi_{p}^{⊤}\Phi_{p})\theta -\frac{1}{n}2\theta^{⊤}{\bar{\Phi }}_{q}+\delta_{3}\|\theta \|\right\},$$

where we use the terminology ‘Riesz regression’ from Chernozhukov et al. (2024). For any parameter $\delta_{2}>0$ and corresponding constrained problem solution $\hat{w}$, there exists a parameter $\delta_{3}>0$ such that $\hat{w}=\delta_{3}\Phi_{p}\hat{\theta }$, where $\hat{\theta }$ is the solution to the Riesz regression form. As a result, for any norm ‖⋅‖, the penalized and constrained forms will always produce weights that are linear in $\Phi_{p}$ (see Ben-Michael, Feller, Hirshberg, et al., 2021, Section 9). Therefore, since the three problems are equivalent, we typically use a generic *δ* to denote the regularization parameter, and will specify the particular form only if necessary. In online supplementary material, Appendix A.2 we illustrate several concrete examples for this problem and in online supplementary material, Appendix D we consider alternative dispersion parameters and discuss popular forms of balancing that constrain the weights to be nonnegative.

Remark 1 **Intercept**

An important constraint in practice is to normalize the weights, $\frac{1}{n}\sum_{i=1}^{n}w_{i}=1$. This corresponds to replacing $\Phi_{p}$ and $\Phi_{q}$ with their centred versions, $\Phi_{p}-{\bar{\Phi }}_{p}$ and $\Phi_{q}-{\bar{\Phi }}_{p}$, in the dual form of the balancing weights problem. This is also equivalent to adding a column of 1s to $\Phi_{p}$. Appropriately accounting for this normalization, however, unnecessarily complicates the notation. Therefore, without loss of generality, we will assume that the features are centred throughout, that is, ${\bar{\Phi }}_{p}=0$.

Remark 2 **Equivalence with kernel ridge regression**

For the special case of $\ell_{2}$ balancing (as in online supplementary material, Appendix A.2) the balancing weights problem is numerically equivalent to directly estimating the conditional expectation $E[Y_{p}|\Phi_{p}]$ via (kernel) ridge regression and applying the estimated coefficients to ${\bar{\Phi }}_{q}$. Moreover, the solution to the balancing weights problem has a closed form that is always linear in ${\bar{\Phi }}_{q}$; we provide further details in online supplementary material, Appendix A.5. For exact balance with δ=0, the balancing weights problem is equivalent to fitting unregularized OLS; see, for example, J. Robins et al. (2007), Kline (2011), and Chattopadhyay et al. (2020).

## 3 Novel equivalence results for (augmented) balancing weights and outcome regression models

Our first main result demonstrates that *any* linear balancing weights estimator is equivalent to applying OLS to the reweighted features. Our second result provides a novel analysis of augmented balancing weights, demonstrating that augmenting any linear balancing weights estimator with a linear outcome regression estimator is equivalent to a plug-in estimator of a new linear model with coefficients that are a weighted combination of estimated OLS coefficients and the coefficients of the original linear outcome model.

### 3.1 Weighting alone

Our first result is that estimating the target estimand ψ(m) with any linear balancing weights is equivalent to fitting OLS for the regression of $Y_{p}$ on $\Phi_{p}$ and then applying those coefficients to the reweighted target feature profile. The key idea for this result begins with the simple unregularized regression prediction for ψ(m), ${\bar{\Phi }}_{q}{\hat{\beta }}_{\mathrm{ols}}$.

Proposition 3.1 Let ${\hat{w}}^{\delta }\;:={\hat{\theta }}^{\delta }\Phi_{p}^{⊤}$, ${\hat{\theta }}^{\delta }\in R^{d}$, be any linear balancing weights, with corresponding weighted features ${\hat{\Phi }}_{q}^{\delta }\;:=\frac{1}{n}{\hat{w}}^{\delta }\Phi_{p}$. Let ${\hat{\beta }}_{\mathrm{ols}}=(\Phi_{p}^{⊤}\Phi_{p}{)}^{†}\Phi_{p}^{⊤}Y_{p}$ be the OLS coefficients of the regression of $Y_{p}$ on $\Phi_{p}$. Then:

$$\begin{array}{rl} \hat{E}\left[{\hat{w}}^{\delta }\circ Y_{p}\right] & ={\hat{\Phi }}_{q}^{\delta }{\hat{\beta }}_{\mathrm{ols}}\\ & =\left({\bar{\Phi }}_{p}+{\hat{\Delta }}^{\delta }\right){\hat{\beta }}_{\mathrm{ols}}, \end{array}$$

where ${\hat{\Delta }}^{\delta }={\hat{\Phi }}_{q}^{\delta }-{\bar{\Phi }}_{p}$ is the mean feature shift implied by the balancing weights and where superscript *δ* indicates possible dependence on a hyperparameter. We have assumed without loss of generality that ${\bar{\Phi }}_{p}=0$, but we sometimes use $\hat{\Delta }$ notation to demonstrate the role of mean feature shift in various expressions. We use the symbol ° to denote element-wise multiplication.

Note that here we have written the OLS coefficients using the pseudo-inverse †. For clarity in the main text, we focus on the full rank setting, where $(\Phi_{p}^{⊤}\Phi_{p}{)}^{†}=(\Phi_{p}^{⊤}\Phi_{p}{)}^{-1}$; we provide a proof for the general setting in online supplementary material, Appendix B.3. In online supplementary material, Appendix D, we extend Proposition 3.1 to nonlinear balancing weights, including those with a nonnegativity constraint.

We can interpret this result via a contrast with standard regularization. Regularized regression models navigate a bias-variance trade-off by regularizing estimated coefficients ${\hat{\beta }}_{\mathrm{reg}}$ relative to ${\hat{\beta }}_{\mathrm{ols}}$, leading to ${\bar{\Phi }}_{q}{\hat{\beta }}_{\mathrm{reg}}$. The balancing weights approach instead keeps ${\hat{\beta }}_{\mathrm{ols}}$ fixed and regularizes the target feature distribution by penalizing the implied feature shift, ${\hat{\Delta }}^{\delta }={\hat{\Phi }}_{q}^{\delta }-{\bar{\Phi }}_{p}$.

We emphasize that this is a new and quite general result. As we discuss in online supplementary material, Appendix A.5, it has been shown previously that for exact balancing weights, $\hat{E}[{\hat{w}}_{\mathrm{exact}}Y_{p}]={\bar{\Phi }}_{q}{\hat{\beta }}_{\mathrm{ols}}$. However, Proposition 3.1 holds for any weights of the form $w=\theta \Phi_{p}^{⊤}$ with arbitrary $\theta \in R^{d}$. In Sections 4 and 5, we consider the particular form of ${\hat{\Phi }}_{q}^{\delta }$ for $\ell_{2}$ and $\ell_{\infty }$ balancing, respectively.

### 3.2 Augmented balancing weights

We can immediately extend this to augmented balancing weights, which regularize *both* the coefficients and the feature shift. Let ${\hat{\beta }}_{\mathrm{reg}}^{\lambda }$ be the coefficients of any regularized linear model for the relationship between $Y_{p}$ and $\Phi_{p}$, where the superscript *λ* indicates dependence on a hyperparameter (e.g. estimated by regularized least squares). We consider augmenting $\hat{E}[{\hat{w}}^{\delta }\circ Y_{p}]$ with ${\hat{\beta }}_{\mathrm{reg}}^{\lambda }$ using the doubly robust functional representation in Equation (3). The augmented estimator is


(7) $$\hat{E}[\Phi_{q}{\hat{\beta }}_{\mathrm{reg}}^{\lambda }]+\hat{E}[{\hat{w}}^{\delta }\circ (Y_{p}-\Phi_{p}{\hat{\beta }}_{\mathrm{reg}}^{\lambda })]=\hat{E}[{\hat{w}}^{\delta }\circ Y_{p}]+\hat{E}\left[\left(\Phi_{q}-{\hat{\Phi }}_{q}^{\delta }\right)\;{\hat{\beta }}_{\mathrm{reg}}^{\lambda }\right].$$

Many recently proposed estimators have this form; see e.g. Athey et al. (2018) and Ben-Michael, Feller, Hirshberg, et al. (2021). If the weighting model and outcome model have different bases, our result applies to a shared basis by either combining the dictionaries as in Chernozhukov et al. (2022a) or by applying an appropriate projection as in Hirshberg and Wager (2021).

We apply Proposition 3.1 to the first term of the right-hand side of (7) to yield the following result. As this result is purely numerical, it applies to arbitrary vectors ${\hat{\beta }}_{\mathrm{reg}}^{\lambda }\in R^{d}$, but substantively we think of ${\hat{\beta }}_{\mathrm{reg}}^{\lambda }$ as the estimated coefficients from an outcome model.

Proposition 3.2 For any ${\hat{\beta }}_{\mathrm{reg}}^{\lambda }\in R^{d}$, and any linear balancing weights estimator with estimated coefficients ${\hat{\theta }}^{\delta }\in R^{d}$, and with ${\hat{w}}^{\delta }\;:={\hat{\theta }}^{\delta }\Phi_{p}^{⊤}$ and ${\hat{\Phi }}_{q}^{\delta }\;:=\frac{1}{n}{\hat{w}}^{\delta }\Phi_{p}$, the resulting augmented estimator

$$\begin{array}{rl} & \hat{E}[{\hat{w}}^{\delta }\circ Y_{p}]+\hat{E}\left[\left(\Phi_{q}-{\hat{\Phi }}_{q}^{\delta }\right){\hat{\beta }}_{\mathrm{reg}}^{\lambda }\right]\\ & \;=\hat{E}\left[{\hat{\Phi }}_{q}^{\delta }{\hat{\beta }}_{\mathrm{ols}}+\left(\Phi_{q}-{\hat{\Phi }}_{q}^{\delta }\right){\hat{\beta }}_{\mathrm{reg}}^{\lambda }\right]\\ & \;=\hat{E}[\Phi_{q}{\hat{\beta }}_{\mathrm{aug}}], \end{array}$$

where the *j*th element of ${\hat{\beta }}_{\mathrm{aug}}$ is

$$\begin{array}{rl} {\hat{\beta }}_{\mathrm{aug},j} & \;:=\left(1-a_{j}^{\delta }\right){\hat{\beta }}_{\mathrm{reg},j}^{\lambda }+a_{j}^{\delta }{\hat{\beta }}_{\mathrm{ols},j}\\ a_{j}^{\delta } & \;:=\frac{{\hat{\Delta }}_{\;j}^{\delta }}{\Delta_{j}}, \end{array}$$

where $\Delta_{j}={\bar{\Phi }}_{q,j}-{\bar{\Phi }}_{\;p,j}$ is the observed mean feature shift for feature *j*; and ${\hat{\Delta }}_{j}^{\delta }={\hat{\Phi }}_{q,j}^{\delta }-{\bar{\Phi }}_{\;p,j}$ is the feature shift for feature *j* implied by the balancing weights model. Finally, $a^{\delta }\in [0,1{]}^{d}$ when the covariance matrix is diagonal, $\left(\Phi_{p}^{⊤}\Phi_{p})=\text{diag}(\sigma_{1}^{2},\sigma_{2}^{2},\ldots ,\sigma_{d}^{2}\right)$, with $\sigma_{j}^{2}>0$.

This is our central numerical result for augmented balancing weights: when both the outcome and weighting models are linear, the augmented estimator is equivalent to a linear model applied to the target features $\Phi_{q}$, with coefficients that are element-wise affine combinations of the base learner coefficients, ${\hat{\beta }}_{\mathrm{reg}}^{\lambda }$, and the coefficients ${\hat{\beta }}_{\mathrm{ols}}$ from an OLS regression of $Y_{p}$ on $\Phi_{p}$. (The coefficients are additionally *convex* combinations of ${\hat{\beta }}_{\mathrm{reg}}^{\lambda }$ and ${\hat{\beta }}_{\mathrm{ols}}$ when the covariance matrix is diagonal.) In Sections 4 and 5, we analyse some of the properties of the augmented estimator for $\ell_{2}$ and $\ell_{\infty }$ balancing weights problems, respectively.

The regularization parameter for the balancing weights problem, *δ*, parametrizes the path between ${\hat{\beta }}_{\mathrm{reg}}^{\lambda }$ and ${\hat{\beta }}_{\mathrm{ols}}$. To see this, consider the cases where δ→0 and δ→∞. As δ→0 the balancing weights problem prioritizes minimizing balance over controlling variance, and ${\hat{\Delta }}_{j}^{\delta }\to \Delta_{j}$ for all *j*. (Recall that we assume ${\bar{\Phi }}_{\;p,j}=0$ for all *j*. Thus, $\Delta_{j}={\bar{\Phi }}_{q,j}$ and ${\hat{\Delta }}_{j}^{\delta }={\hat{\Phi }}_{q,j}^{\delta }$. So ${\hat{\Delta }}_{\;j}^{\delta }\to \Delta_{j}$ is equivalent to ${\hat{\Phi }}_{q}^{\delta }\to {\bar{\Phi }}_{q,j}$.) In this case, $a_{j}^{\delta }={\hat{\Delta }}_{j}^{\delta }/\Delta_{j}\to 1$, and the weights fully ‘de-bias’ the original outcome model by recovering unregularized regression, ${\hat{\beta }}_{\mathrm{aug}}\to {\hat{\beta }}_{\mathrm{ols}}$. In Section 7.2, we will see that when chosen by cross-validation, *δ* sometimes equals exactly 0 in applied problems; thus even when ${\hat{\beta }}_{\mathrm{reg}}^{\lambda }$ is a sophisticated regularized estimator, the final augmented point estimate can nonetheless be numerically equivalent to the simple OLS plug-in estimate. Conversely, as δ→∞, the balancing weights problem prioritizes controlling variance, leading to uniform weights and ${\hat{\Delta }}_{j}\to 0$. In this case, $a_{j}^{\delta }={\hat{\Delta }}_{j}^{\delta }/\Delta_{j}\to 0$, the weighting model does very little, and ${\hat{\beta }}_{\mathrm{aug}}\to {\hat{\beta }}_{\mathrm{reg}}^{\lambda }$.

It is also instructive to consider two other extremes: unregularized outcome model and unregularized balancing weights. First, consider the special case of fitting an unregularized linear regression outcome model, i.e. ${\hat{\beta }}_{\mathrm{reg}}^{\lambda }={\hat{\beta }}_{\mathrm{ols}}$. Then Proposition 3.2 reproduces the result, originally due to J. Robins et al. (2007), that ‘OLS is doubly robust’ (see also Kline, 2011). This is because ${\hat{\beta }}_{\mathrm{aug}}={\hat{\beta }}_{\mathrm{ols}}$ for arbitrary linear weights ${\hat{\theta }}^{\delta }\in R^{d}$. Thus, OLS augmented by *any* choice of linear balancing weights collapses to OLS alone. Equivalently, we can view OLS alone as an augmented estimator that combines an OLS base learner with linear balancing weights.

A similar result holds for unregularized balancing weights, i.e. exact balancing weights. Let ${\hat{w}}_{\mathrm{exact}}$ be the solution to a balancing weights problem in Section 2.3 with hyperparameter δ=0, and let ${\hat{\beta }}_{\mathrm{reg}}^{\lambda }\in R^{d}$ be arbitrary coefficients. Then from the balance condition, ${\hat{\Phi }}_{q}={\bar{\Phi }}_{q}$, $a_{j}^{\delta }=1$ for all *j*, and we have that ${\hat{\beta }}_{\mathrm{aug}}={\hat{\beta }}_{\mathrm{ols}}$. Thus, the augmented exact balancing weights estimator also collapses to the OLS regression estimator. Equivalently, the augmented exact balancing weights estimator collapses to the *unaugmented* exact balancing weights estimator. Zhao and Percival (2017) use a very similar result to argue that entropy balancing, a form of exact balancing weights, is DR.

Finally, before we turn to new results for $\ell_{2}$ and $\ell_{\infty }$ balancing, we briefly comment on several points that are discussed in more detail in the online supplementary material, Appendix.

Remark 3 **Sample splitting**

Sample splitting is a common technique in the AutoDML literature especially, in which we only apply the outcome and weighting models to data points not used for estimation; see, for example, Newey and Robins (2018) and Chernozhukov et al. (2022a). Since Proposition 3.2 holds for arbitrary vectors ${\hat{\beta }}_{\mathrm{reg}}^{\lambda }$ and ${\hat{\theta }}^{\delta }$, the results still hold under cross-fitting. See online supplementary material, Appendix C for an extended discussion.

Remark 4 **Infinite-dimensional setting**

While we emphasize the linear, low-dimensional setting where $\Phi_{p}^{⊤}\Phi_{p}$ is invertible, Proposition 3.2 holds far more broadly. The result remains true when the function class F is a subset of *any* Hilbert space. This includes the high-dimensional setting where d>n and the infinite dimensional setting. See online supplementary material, Appendix B for a formal statement.

Remark 5 **Nonlinear balancing weights**

A rich tradition in survey statistics (e.g. Deville & Särndal, 1992), machine learning (e.g. Menon & Ong, 2016), and causal inference (e.g. Tan, 2020; Vermeulen & Vansteelandt, 2015; Zhao, 2019) focuses on *nonlinear* balancing weights, such as when the weights correspond to a specific *link function*  g(⋅) applied to the linear predictor, $\hat{w}=g(\hat{\theta }\Phi_{p}^{⊤})$, or, equivalently, when the balancing weights problem penalizes an alternative dispersion penalty. In online supplementary material, Appendix D, we briefly consider extending Proposition 3.1 to nonlinear weights and show that the nonlinearity introduces an additional approximation error. A more thorough extension is a promising direction for future research.

Remark 6 **Nonnegative weights**

A common modification of the (minimum variance) balancing weights problem is to constrain the estimated weights to be nonnegative or on the simplex; examples include Stable Balancing Weights (Zubizarreta, 2015) and the Synthetic Control Method (Abadie et al., 2010), as well as their augmented analogues (Athey et al., 2018; Ben-Michael, Feller, Rothstein, 2021). Such weights have a number of attractive practical properties: they limit extrapolation; they ensure that the final weighting estimator is sample bounded; and they are typically sparse, which can sometimes aid interpretability (J. Robins et al., 2007). In online supplementary material, Appendix D.2, we extend Proposition 3.1 and show that restricting weights to be nonnegative is equivalent to sample trimming. In particular, let ${\hat{w}}_{+}^{\delta }$ be the estimated nonnegative weights and ${\hat{\beta }}_{\mathrm{ols}}^{+}$ be the OLS coefficient of the regression of $Y_{p}$ on $\Phi_{p}$, but restricted to units with positive weight. Then, Proposition 3.1 continues to hold, but with ${\hat{\beta }}_{\mathrm{ols}}^{+}$ in place of the unrestricted ${\hat{\beta }}_{\mathrm{ols}}$: $\hat{E}[{\hat{w}}_{+}^{\delta }\circ Y_{p}]={\hat{\Phi }}_{q}^{\delta }{\hat{\beta }}_{\mathrm{ols}}^{+}$.

Remark 7 **Bilinear form**

As pointed out by a reviewer, (many of) the functionals we consider can be written as a bilinear form $\alpha^{T}\Sigma \beta$ where *β* is the coefficient for the outcome model, *α* is the coefficient for the Riesz representer and Σ is the some weighted population Gram matrix (J. Robins et al., 2008); for E[Y(1)], it would be $E[Z\phi (X)\phi {\left(X\right)}^{T}]$. Proposition 3.2 suggests that *β* can be estimated using the methods we discuss here, and moreover that the aggregation weights would then be entangled with Σ or *α*. Understanding whether this could be used to then motivate new estimators is an interesting topic for future work.

## 4 Augmented $\ell_{2}$ balancing weights

In this section, we study $\ell_{2}$ balancing weights estimators, which are commonly used in the context of kernel balancing (Ben-Michael, 2024; Gretton et al., 2012; Hirshberg et al., 2019; Kallus, 2020) and for panel data methods (Abadie et al., 2010; Ben-Michael, Feller, Rothstein, 2021). We first show that the regularization path $a_{j}^{\delta }$ from Proposition 3.2 follows typical ridge regression shrinkage, with a smooth decay. Moreover, augmenting with $\ell_{2}$ balancing weights is equivalent to boosting with ridge regression, and always overfits relative to the unaugmented outcome model alone. We then show that when the outcome model used to augment $\ell_{2}$ balancing weights is also a ridge regression (which we refer to as ‘double ridge’), the augmented estimator is itself equivalent to a single, generalized ridge regression, albeit undersmoothed relative to the base learner. These results extend immediately to the RKHS setting of ‘double kernel ridge’ estimation, combining kernel balancing weights and kernel ridge regression. In Section 6, we show the implications of these numeric results for undersmoothing in the statistical sense.

While the following results hold for arbitrary covariance matrices, in the main text we simplify the presentation by assuming that $\Phi_{p}^{⊤}\Phi_{p}$ is diagonal; that is, $\left(\Phi_{p}^{⊤}\Phi_{p})=\text{diag}(\sigma_{1}^{2},\sigma_{2}^{2},\ldots ,\sigma_{d}^{2}\right)$, with $\sigma_{j}^{2}>0$. We show that this is without loss of generality for $\ell_{2}$ balancing in online supplementary material, Appendix E.

### 4.1 General linear outcome model

Following Remark 2, $\ell_{2}$ balancing weights, including kernel balancing weights, have a closed form that is always linear in ${\bar{\Phi }}_{q}$. Our next result applies this closed form to Proposition 3.2 to derive the regularization path that results from augmenting an arbitrary linear outcome model with $\ell_{2}$ balancing weights. Although this is an immediate consequence of Proposition 3.2, the resulting form of the augmented estimator has unique structure that warrants a new result.

Proposition 4.1 Let ${\hat{w}}_{\ell_{2}}^{\delta }$ be (penalized) linear balancing weights with regularization parameter *δ* and $F=\{f(x)=\theta^{⊤}\phi (x)\;:\;\|\theta {\|}_{2}\leq 1\}$. Then $\frac{1}{n}{\hat{w}}_{\ell_{2}}^{\delta }={\bar{\Phi }}_{q}(\Phi_{p}^{⊤}\Phi_{p}+\delta I{)}^{-1}\Phi_{p}^{⊤}.$ Therefore, the augmented $\ell_{2}$ balancing weights estimator with outcome model ${\hat{\beta }}_{\mathrm{reg}}^{\lambda }\in R^{d}$ has the form

$$\hat{E}[\Phi_{q}{\hat{\beta }}_{\mathrm{reg}}^{\lambda }]+\hat{E}[{\hat{w}}_{\ell_{2}}^{\delta }(Y_{p}-\Phi_{p}{\hat{\beta }}_{\mathrm{reg}}^{\lambda })]=\hat{E}[\Phi_{q}{\hat{\beta }}_{\ell_{2}}],$$

where the *jth* coefficient of ${\hat{\beta }}_{\ell_{2}}$ is given by

(8) $$\begin{array}{rl} {\hat{\beta }}_{\ell_{2},j} & \;:=\left(1-a_{j}^{\delta }\right){\hat{\beta }}_{\mathrm{reg},j}^{\lambda }+a_{j}^{\delta }{\hat{\beta }}_{\mathrm{ols},j}\\ a_{j}^{\delta } & \;:=\frac{\sigma_{j}^{2}}{\sigma_{j}^{2}+\delta }. \end{array}$$

In this case, the $a_{j}^{\delta }$ are exactly equal to the standard regularization path of ridge regression. To see this, recall that ridge regression with penalty *δ* shrinks the ${\hat{\beta }}_{\mathrm{ols}}$ coefficients as follows:


(9) $${\hat{\beta }}_{\mathrm{ridge},j}^{\delta }=\left(\frac{\sigma_{j}^{2}}{\sigma_{j}^{2}+\delta }\right){\hat{\beta }}_{\mathrm{ols},j}=a_{j}^{\delta }{\hat{\beta }}_{\mathrm{ols},j}.$$

This is identical to the expression in (8) but with ${\hat{\beta }}_{\mathrm{reg}}^{\lambda }$ set to 0: Ridge regression shrinks ${\hat{\beta }}_{\mathrm{ols}}$ towards 0 with regularization path $a_{j}^{\delta }$, while $\ell_{2}$ augmenting shrinks ${\hat{\beta }}_{\mathrm{ols}}$ towards ${\hat{\beta }}_{\mathrm{reg}}^{\lambda }$ with the same regularization path.

**Figure 1.**
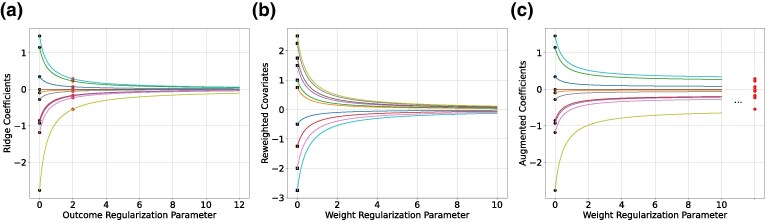
Regularization paths for ‘double ridge’ augmented $\ell_{2}$ balancing weights. Panel (a) shows the coefficients ${\hat{\beta }}_{\mathrm{reg}}^{\lambda }$ of a ridge regression of $Y_{p}$ on $\Phi_{p}$ with hyperparameter *λ*. The dots on the left are the ordinary least-squares (OLS) coefficients, with λ=0. The dots at λ=2 illustrate the coefficients at a plausible hyperparameter value, ${\hat{\beta }}_{\mathrm{reg}}^{2}$. Panel (b) shows reweighted covariates, ${\hat{\Phi }}_{q}^{\delta }$, for the $\ell_{2}$ balancing weights problem with hyperparameter *δ*; the squares on the left show exact balance, which corresponds to OLS. As *δ* increases, the weights converge to uniform weights and ${\hat{\Phi }}_{q}^{\delta }$ converges to ${\bar{\Phi }}_{p}$, which we have centred at zero. Panel (c) shows the augmented coefficients, ${\hat{\beta }}_{\ell_{2}}$ as a function of the weight regularization parameter *δ*. The dots on the left are the OLS coefficients. As δ→∞, the coefficients converge to ${\hat{\beta }}_{\mathrm{reg}}^{2}$. All three regularization paths have essentially identical qualitative behaviour. (a) Outcome model, (b) weighting model and (c) augmented model.

As an illustration, the right panel of Figure 1 shows ${\hat{\beta }}_{\ell_{2}}$ (on the *y*-axis) for 10 covariates, with *δ* increasing from 0 (on the *x*-axis). The dots on the left pick out ${\hat{\beta }}_{\mathrm{ols}}$; when δ=0, then $a_{j}^{0}=1$ and ${\hat{\beta }}_{\ell_{2}}={\hat{\beta }}_{\mathrm{ols}}$. The limit on the right shows ${\hat{\beta }}_{\mathrm{reg}}^{\lambda }$. The smooth regularization path is characteristic of ridge regression shrinkage.

We can also view ${\hat{\beta }}_{\ell_{2}}$ as the output of a single iteration of a ridge boosting procedure, fit using $Y_{p}$ and $\Phi_{p}$ alone. See Bühlmann and Yu (2003) and Park et al. (2009) for detailed discussion; Newey et al. (2004) make a similar connection in the context of twicing kernels.

Proposition 4.2 Let ${\overset{ˇ}{Y}}_{p}=Y_{p}-\Phi_{p}{\hat{\beta }}_{\mathrm{reg}}^{\lambda }$ be the residuals from the base learner. Let ${\hat{\beta }}_{\mathrm{boost}}^{\delta }$ be the coefficients from the ridge regression of ${\overset{ˇ}{Y}}_{p}$ on $\Phi_{p}$ with hyperparameter *δ*. Then, ${\hat{\beta }}_{\ell_{2}}={\hat{\beta }}_{\mathrm{reg}}^{\lambda }+{\hat{\beta }}_{\mathrm{boost}}^{\delta },$ and $\|Y_{p}-\Phi_{p}{\hat{\beta }}_{\ell_{2}}{\|}_{2}^{2}\leq \|Y_{p}-\Phi_{p}{\hat{\beta }}_{\mathrm{reg}}^{\lambda }{\|}_{2}^{2}.$

So for a fixed *δ*, the augmented $\ell_{2}$ balancing estimator is equivalent to estimating a new outcome model coefficient estimator ${\hat{\beta }}_{\ell_{2}}$ that *overfits* relative to ${\hat{\beta }}_{\mathrm{reg}}^{\lambda }$ (in the sense of having smaller in-sample training error), and then applying that model to $\Phi_{q}$.

Surprisingly—and in contrast to the general result in Proposition 3.2—the augmented coefficients ${\hat{\beta }}_{\ell_{2}}$ are the same for *every* target covariate profile $\Phi_{q}$. To see this, note that Proposition 4.1 shows that $\ell_{2}$ balancing weights are always linear in ${\bar{\Phi }}_{q}$. Therefore, the corresponding regularization path $a_{j}^{\delta }$ does not depend on the target profile $\Phi_{q}$; it depends only on *δ* and the source distribution variances $\sigma_{j}^{2}$. This property is closely related to *universal adaptability* in the computer science literature on multigroup fairness (Kim et al., 2022). The particular $\Phi_{q}$ may nonetheless impact the choice of *δ* in hyperparameter selection, e.g. via cross-validating imbalance, which in turn influences the degree of overfitting; we do find this to be the case theoretically in Section 6.2.

### 4.2 Ridge regression outcome model

Proposition 4.1 holds for arbitrary linear outcome model coefficient estimators ${\hat{\beta }}_{\mathrm{reg}}^{\lambda }\in R^{d}$; we now state the corresponding result for a ‘double ridge’ estimator, where the base learner outcome model is itself fit via ridge regression. The key takeaway is that the implied augmented coefficients are *undersmoothed* relative to the base learner ridge coefficients.

For this section, we will consider the following generalized ridge regression, sometimes known as ‘adaptive’ ridge regression (Grandvalet, 1998). Let $\Lambda \in R^{d\times d}$ be a diagonal matrix with *j*th diagonal entry $\lambda_{j}\geq 0$. Then the generalized ridge coefficients are:


$$\begin{array}{rl} {\hat{\beta }}_{\mathrm{ridge}}^{\Lambda } & \;:=\underset{\beta \in R^{d}}{\text{argmin}}\;\|\Phi_{p}\beta -Y_{p}{\|}_{2}^{2}+\beta^{⊤}\Lambda \beta \\ & =(\Phi_{p}^{⊤}\Phi_{p}+\Lambda {)}^{-1}\Phi_{p}^{⊤}Y_{p}. \end{array}$$

Standard ridge regression is the special case where the $\lambda_{j}$ all take the same value and so Λ=λI. As above, the generalized ridge coefficients can be rewritten as shrinking the OLS coefficients:


(10) $${\hat{\beta }}_{\mathrm{ridge},j}^{\Lambda }=\left(\frac{\sigma_{j}^{2}}{\sigma_{j}^{2}+\lambda_{j}}\right){\hat{\beta }}_{\mathrm{ols},j}.$$

We now demonstrate that the augmented $\ell_{2}$ balancing weights estimator with base learner ${\hat{\beta }}_{\mathrm{ridge}}^{\Lambda }$ is equivalent to a plug-in estimator using generalized ridge with *smaller* hyperparameters, ${\hat{\beta }}_{\mathrm{ridge}}^{\Gamma }$, where *Γ* is a diagonal matrix with *j*th diagonal entry $\gamma_{j}\in [0,\lambda_{j}]$.

Proposition 4.3 Let ${\hat{\beta }}_{\mathrm{ridge}}^{\Lambda }$ denote the coefficients of a generalized ridge regression of $Y_{p}$ on $\Phi_{p}$ with hyperparameters Λ, and let ${\hat{w}}_{\ell_{2}}^{\delta }$ denote $\ell_{2}$ balancing weights with hyperparameter *δ* defined in Section 2.3. Define the diagonal matrix *Γ* with *j*th diagonal entry:

$$\gamma_{j}\;:=\frac{\delta \lambda_{j}}{\sigma_{j}^{2}+\lambda_{j}+\delta }\leq \lambda_{j}.$$

Then:

$$\hat{E}[\Phi_{q}{\hat{\beta }}_{\mathrm{ridge}}^{\Lambda }]+\hat{E}[{\hat{w}}_{\ell_{2}}^{\delta }(Y_{p}-\Phi_{p}{\hat{\beta }}_{\mathrm{ridge}}^{\Lambda })]=\hat{E}[\Phi_{q}{\hat{\beta }}_{\mathrm{ridge}}^{\Gamma }].$$

Furthermore, ${\hat{\beta }}_{\mathrm{ridge}}^{\Gamma }$ are standard ridge regression coefficients (i.e. $\gamma_{j}$ is a constant for all *j*) when $\lambda_{j}=\lambda$ and $\sigma_{j}=\sigma$ for all *j*.

The same result holds for kernel ridge regression; see online supplementary material, Appendix B.4.

In this setting, augmenting with balancing weights is equivalent to undersmoothing the original outcome model fit. In particular, we can use the expansion in Equation (10) to see the undersmoothing in ${\hat{\beta }}_{\mathrm{ridge}}^{\Gamma }$ explicitly:


$$\frac{\sigma_{j}^{2}}{\sigma_{j}^{2}+\gamma_{j}}=\underset{\mathrm{outcome}\;\mathrm{model}}{\underbrace{\left(\frac{\sigma_{j}^{2}}{\sigma_{j}^{2}+\lambda_{j}}\right)}}\underset{\mathrm{augmentation}}{\underbrace{\left(\frac{\sigma_{j}^{2}+\lambda_{j}+\delta }{\sigma_{j}^{2}+\delta }\right)}},$$

where the first term is the shrinkage from the original generalized ridge model alone, and the second term is due to augmenting with $\ell_{2}$ balancing weights. Importantly, the second term is in $\left[1,\frac{\sigma_{j}^{2}+\lambda_{j}}{\sigma_{j}^{2}}\right]$ and therefore partially reverses the shrinkage of the original estimate. In Section 6.1, we connect this to undersmoothing in the statistical sense.

## 5 Augmented $\ell_{\infty }$ balancing weights

In this section, we study $\ell_{\infty }$ balancing weights estimators, which are widely used in the balancing weights literature (Athey et al., 2018; Zubizarreta, 2015) and in the AutoDML literature (Chernozhukov et al., 2022a). In the main text, we consider the special case where the covariance matrix $\Phi_{p}^{⊤}\Phi_{p}$ is diagonal; that is, $\left(\Phi_{p}^{⊤}\Phi_{p})=\text{diag}(\sigma_{1}^{2},\sigma_{2}^{2},\ldots ,\sigma_{d}^{2}\right)$, with $\sigma_{j}^{2}>0$. Unlike with $\ell_{2}$ balancing, this is no longer without loss of generality. We discuss this general case in online supplementary material, Appendix E.3.

For diagonal covariance, we first show that $\ell_{\infty }$ balancing has a closed form: it is equivalent to applying a soft-thresholding operator to the feature shift from ${\bar{\Phi }}_{p}$ to ${\bar{\Phi }}_{q}$. We then write the resulting augmented estimator as applying coefficients ${\hat{\beta }}_{\ell_{\infty }}$ to $\Phi_{q}$ and show that ${\hat{\beta }}_{\ell_{\infty }}$ is a sparse, element-wise convex combination of the base learner coefficients and OLS coefficients. When the outcome model is also fit via the lasso, we use the resulting representation to demonstrate a familiar ‘double selection’ phenomenon (Belloni et al., 2014), where ${\hat{\beta }}_{\ell_{\infty }}$ inherits the nonzero coefficients of both the base learner and the weighting model. This is a form of undersmoothing in the $\ell_{0}$ ‘norm’, in the sense that ${\hat{\beta }}_{\ell_{\infty }}$ always has at least as many nonzero coefficients as the base learner, ${\hat{\beta }}_{\mathrm{reg}}$.

### 5.1 Weighting alone

We first define the soft-thresholding operator and show that the $\ell_{\infty }$ balancing problem has a closed-form solution.

Definition **Soft-thresholding operator**

For t>0, define the soft-thresholding operator,

$$T_{t}(z)\;:=\left\{ \begin{array}{ll} 0 & \text{if }|z|<t\\ z-t & \text{if }z>t\\ z+t & \text{if }z<-t \end{array}\right..$$

Proposition 5.1 **
$\ell_{\infty }$ Balancing**

If $\Phi_{p}^{⊤}\Phi_{p}$ is diagonal, the solution $w_{\ell_{\infty }}^{\delta }$ to the $\ell_{\infty }$ optimization problem online supplementary material, (3) is

$$\begin{array}{rl} \frac{1}{n}w_{\ell_{\infty }}^{\delta } & =\Phi_{p}(\Phi_{p}^{⊤}\Phi_{p}{)}^{-1}\left[{\bar{\Phi }}_{p}+T_{\delta }({\bar{\Phi }}_{q}-{\bar{\Phi }}_{p})\right]\\ & =\Phi_{p}(\Phi_{p}^{⊤}\Phi_{p}{)}^{-1}\left[{\bar{\Phi }}_{p}+T_{\delta }(\Delta )\right], \end{array}$$

where $\Delta ={\bar{\Phi }}_{q}-{\bar{\Phi }}_{p}$, where we include ${\bar{\Phi }}_{p}$ (equal to 0 by assumption) to emphasize the dependence on feature shift, and with corresponding reweighted features, ${\hat{\Phi }}_{q}^{\delta }={\bar{\Phi }}_{p}+T_{\delta }({\bar{\Phi }}_{q}-{\bar{\Phi }}_{p})$.

For intuition, compare the (unaugmented) $\ell_{\infty }$ balancing weights estimator to the lasso-based coefficient estimates (Hastie et al., 2009):


$$\begin{array}{rl} \hat{E}[w_{\ell_{\infty }}^{\delta }\circ Y_{p}] & =T_{\delta }({\bar{\Phi }}_{q}{)}^{⊤}{\hat{\beta }}_{\mathrm{ols}}\\ \hat{E}[\Phi_{q}{\hat{\beta }}_{\mathrm{lasso}}^{\lambda }] & ={{\bar{\Phi }}_{q}}^{⊤}T_{\lambda }({\hat{\beta }}_{\mathrm{ols}}), \end{array}$$

where we simplify ${\hat{\Phi }}_{q}^{\delta }$ here to emphasize the connections between the methods. Whereas lasso performs soft-thresholding on the OLS coefficients (regularizing the outcome regression), $\ell_{\infty }$ balancing performs soft-thresholding on the implied feature shift to the target features.

### 5.2 General linear outcome model

We can then plug the closed-form solution for the weights into Proposition 3.2.

Proposition 5.2 Let ${\hat{w}}_{\ell_{\infty }}^{\delta }$ be defined as above. Then the augmented $\ell_{\infty }$ balancing weights estimator with outcome model fit ${\hat{\beta }}_{\mathrm{reg}}^{\lambda }\in R^{d}$ has the form,

$$\hat{E}[\Phi_{q}{\hat{\beta }}_{\mathrm{reg}}^{\lambda }]+\hat{E}[{\hat{w}}_{\ell_{\infty }}^{\delta }(Y_{p}-\Phi_{p}{\hat{\beta }}_{\mathrm{reg}}^{\lambda })]=\hat{E}[\Phi_{q}{\hat{\beta }}_{\ell_{\infty }}],$$

where the *jth* coefficient of ${\hat{\beta }}_{\ell_{\infty }}$ equals:

$${\hat{\beta }}_{\ell_{\infty },j}=\left\{ \begin{array}{ll} {\hat{\beta }}_{\mathrm{reg},j}^{\lambda } & \text{if }|\Delta_{j}|<\delta \\ \left|\frac{\delta }{\Delta_{j}}\right|{\hat{\beta }}_{\mathrm{reg},j}^{\lambda }+\left(1-\left|\frac{\delta }{\Delta_{j}}\right|\right){\hat{\beta }}_{\mathrm{ols},j} & \text{otherwise} \end{array}\right.,$$

where $\Delta_{j}={\bar{\Phi }}_{q,j}-{\bar{\Phi }}_{\;p,j}$.

The augmented coefficients ${\hat{\beta }}_{\ell_{\infty }}$ are an element-wise convex combination of ${\hat{\beta }}_{\mathrm{reg}}^{\lambda }$ and ${\hat{\beta }}_{\mathrm{ols}}$. For features where the mean feature shift $\Delta_{j}$ is small (relative to *δ*), ${\hat{\beta }}_{\ell_{\infty }}$ is equivalent to the base learner coefficient ${\hat{\beta }}_{\mathrm{reg}}^{\lambda }$. The remaining coefficients are interpolated linearly toward the ${\hat{\beta }}_{\mathrm{ols}}$ coefficients.

**Figure 2.**
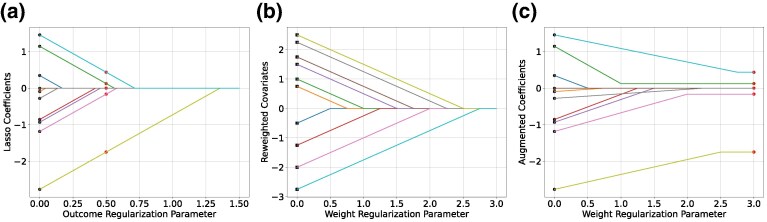
Regularization paths for ‘double lasso’ augmented $\ell_{\infty }$ balancing weights. Panel (a) shows the coefficients ${\hat{\beta }}_{\mathrm{reg}}^{\lambda }$ of a lasso regression of $Y_{p}$ on $\Phi_{p}$ with hyperparameter *λ*. The dots on the left are the ordinary least-squares (OLS) coefficients, with λ=0. The dots at λ=0.5 illustrate the coefficients at a plausible hyperparameter value, ${\hat{\beta }}_{\mathrm{reg}}^{0.5}$. Panel (b) shows reweighted covariates, ${\hat{\Phi }}_{q}^{\delta }$, for the $\ell_{\infty }$ balancing weights problem with hyperparameter *δ*; the squares on the left show exact balance, which corresponds to OLS. As *δ* increases, the weights converge to uniform weights and ${\hat{\Phi }}_{q}^{\delta }$ converges to ${\bar{\Phi }}_{p}$, which we have centred at zero. Panel (c) shows the augmented coefficients, ${\hat{\beta }}_{\ell_{\infty }}$ as a function of the weight regularization parameter *δ*. The dots on the left are the OLS coefficients. As δ→∞, the coefficients converge to ${\hat{\beta }}_{\mathrm{reg}}^{0.5}$. All three regularization paths show the typical lasso ‘soft thresholding’ behaviour. The regularization path for the augmented estimator also shows ‘double selection’ behaviour. (a) Outcome model, (b) weighting model and (c) augmented model.

Figure 2 summarizes these results and their implications for the augmented estimator. As with Figure 1, we generate simple simulated data with d=10. In the left panel, we plot the coefficients from lasso regression of $Y_{p}$ on $\Phi_{p}$ as a function of the lasso regularization parameter. The regularization path begins with the black dots, which represent the OLS coefficients. Each lasso coefficient (represented by a coloured line) then shrinks linearly to exactly zero, due to the soft-thresholding operator. The middle panel plots the reweighted covariates using $\ell_{\infty }$ balancing weights between $\Phi_{p}$ and $\Phi_{q}$ solved in the constrained form. The black dots represent ${\bar{\Phi }}_{q}$, corresponding to exact balance. Then as the weight regularization parameter increases, the reweighted covariates shrink linearly to exactly zero, just as in lasso. The right panel plots coefficients for the augmented estimator that combines a baseline outcome model fit ${\hat{\beta }}_{\mathrm{reg}}^{\lambda }$ with $\ell_{\infty }$ balancing weights. The lines correspond to ${\hat{\beta }}_{\ell_{\infty }}$ as defined in Proposition 5.2. The regularization path begins at the black dots, where ${\hat{\beta }}_{\ell_{\infty }}={\hat{\beta }}_{\mathrm{ols}}$, and eventually converges to ${\hat{\beta }}_{\mathrm{reg}}^{\lambda }$, showing the usual soft-thresholding behaviour. The order at which the coefficients go to zero reflects the size of ${\bar{\Phi }}_{q}$, because the regularization path depends on the weight coefficients from the middle panel. Thus, the augmented estimator shrinks ${\hat{\beta }}_{\mathrm{ols}}$ toward ${\hat{\beta }}_{\mathrm{reg}}^{\lambda }$ but via a soft-thresholding operator applied to the feature shift, $\Delta_{j}$.

### 5.3 Lasso outcome model

In the case where ${\hat{\beta }}_{\mathrm{reg}}^{\lambda }$ is itself fit via lasso, as studied in Chernozhukov et al. (2022a), then we recover a familiar double selection phenomenon (Belloni et al., 2014).

Proposition 5.3 **Double Selection**

Let ${\hat{\beta }}_{\mathrm{lasso}}^{\lambda }$ denote the coefficients of lasso regression of $Y_{p}$ on $\Phi_{p}$ with regularization parameter *λ*. Denote the indices of the nonzero coefficients as $I_{\lambda }$. Let ${\hat{w}}_{\ell_{\infty }}^{\delta }$ be $\ell_{\infty }$ balancing weights with parameter *δ* as in Proposition 5.1. Let $I_{\delta }$ denote the nonzero entries of the reweighted covariates ${\hat{\Phi }}_{q}$. Assume that ${\hat{\beta }}_{\mathrm{ols}}$ is dense. Then the indices of the nonzero entries of the augmented coefficients ${\hat{\beta }}_{\ell_{\infty }}$ are $I_{\mathrm{aug}}=I_{\lambda }\cup I_{\delta }.$

The lasso coefficients have a sparsity pattern generated by soft-thresholding the OLS coefficients. The augmented estimator then shrinks from OLS toward ${\hat{\beta }}_{\mathrm{reg}}^{\lambda }$ by soft-thresholding the implied feature shift to the target features. As a result, wherever the lasso coefficients are nonzero *or* the weight coefficients are nonzero, the final augmented coefficients are also nonzero. The ‘included coefficients’ for the final estimator are then the union of the coefficients included in either individual model. Therefore, augmenting a lasso outcome model with $\ell_{\infty }$ balancing also exhibits a form of undersmoothing in the $\ell_{0}$ ‘norm,’ $\|{\hat{\beta }}_{\ell_{\infty }}{\|}_{0}$, in the sense that there are always at least as many nonzero coefficients as for the unaugmented lasso outcome model. However, this will not correspond to undersmoothing the base learner in the traditional sense, because, in general, there will not exist a lasso hyperparameter *λ* that will produce sparsity pattern $I_{\mathrm{aug}}$.

As noted by, for example, Tang et al. (2023), the double selection estimator may suffer from imprecision due to adjustment for covariates that are associated with treatment but not outcome. One could, in principle, remove covariates that are only predictive of the treatment, but this can jeopardize statistical inference. See Moosavi et al. (2023) for further discussion on this trade-off.

## 6 Kernel ridge regression: asymptotic and finite sample analysis

The results above are *numerical*: they hold without any statistical or causal assumptions. However, the connection between augmented estimators and outcome models also presents *statistical* insights that we discuss here. In particular, we leverage the numerical result that double (kernel) ridge regression—which uses ridge regression for fitting both the outcome and weighting models—is equivalent to a single, undersmoothed outcome ridge regression plug-in estimator.

First, we consider an asymptotic analysis in Section 6.1: we use this equivalence to make explicit the connection between asymptotic results for augmented balancing weights with kernel ridge regression and prior results on optimal undersmoothing of a kernel ridge plug-in estimator. As a result, optimally undersmoothed kernel ridge regression inherits guarantees from augmented ridge regression. An implication is that we can generalize the insight from J. Robins et al. (2007) that ‘OLS is DR’ to a wider class of nonparametric estimators. This equivalence also suggests an appropriate hyperparameter scheme when the outcome regression is an element of an RKHS.

Second, we consider a finite sample analysis in Section 6.2: we use this equivalence to derive the finite-sample design-conditional mean squared error of augmented kernel ridge regression. We then use this expression to characterize finite-sample-optimal hyperparameter tuning. We turn to hyperparameter tuning in practice in the next section.

### 6.1 Asymptotic results

We now use our results in Proposition 4.3 to make explicit the connection between two otherwise distinct sets of asymptotic results. First, Wong and Chan (2018) and Singh (2024) argue that double kernel ridge regression can deliver $\sqrt{n}$-consistent estimation of functionals in certain scenarios. Wong and Chan (2018) also proposes an optimally undersmoothed $\ell_{2}$ balancing weights estimator. Separately, Hirshberg et al. (2019) and Mou et al. (2023) propose optimally undersmoothed (single) kernel ridge outcome regression. Since, as we have shown in Proposition 4.3 (see also Remark 2), these three procedures are equivalent, we can connect these results and show that plug-in estimators based on optimally undersmoothed kernel ridge regression or $\ell_{2}$ balancing weights can be $\sqrt{n}$-consistent. Moveover, results on RKHSs suggest a simple heuristic for hyperparameter choice. We give the high-level argument here and defer additional technical details to online supplementary material, Appendix L.

Assume that the outcome model, m(x,z):=E[Y∣X=x,Z=z], belongs to an RKHS H with kernel *k*, and that we observe *n* iid samples of $\left(x_{i},y_{i},z_{i}\right)$ from *p*. Define $K\in R^{n\times n}$ to be the kernel matrix with i,jth entry $K_{ij}=k((x_{i},z_{i}),(x_{j},z_{j}))$. Let $\sigma_{j}^{2}$ denote the eigenvalues of *K*. We assume that $\sigma_{j}^{2}=\sigma^{2}>0$ is constant for all *j*; we can relax this at the cost of additional complexity. The ‘single kernel ridge’ regression outcome regression estimator with parameter *λ* has coefficient estimates:


$${\hat{\beta }}_{\mathrm{ridge}}^{\lambda }=(K+\lambda I{)}^{-1}y.$$

Applying Proposition 4.3, the augmented ‘double kernel ridge’ estimator with hyperparameter *δ* is equivalent to a plug-in estimate for a new kernel ridge model:


$${\hat{\beta }}_{\mathrm{aug}}=(K+\gamma I{)}^{-1}y,\;\text{with}\;\gamma =\frac{\lambda \delta }{\sigma^{2}+\lambda +\delta }.$$

We can now use modern rate results for kernel ridge regression to explicitly link double kernel ridge and efficiently undersmoothed kernel ridge. First, if we choose hyperparameter schedule $\lambda_{n}$ for kernel ridge regression as in Fischer and Steinwart (2020), we obtain a corresponding convergence rate for the outcome model (see online supplementary material, Appendix L for specifics). Second, we can use the hyperparameter schedule $\delta_{n}$ and Theorem 1 from Singh (2024) to establish a convergence rate for the Riesz representer. Finally by Theorem 4.2 of Chernozhukov et al. (2022b), the augmented estimator that combines these two kernel ridge nuisance estimates is $\sqrt{n}$-consistent. Note that while in this discussion we assume well specification, i.e. m∈H, these rates are *model-agnostic*; similar results hold when m∉H (see Singh, 2024).

Applying our numerical results, the augmented estimator is also a kernel ridge estimator with a new (undersmoothed) hyperparameter schedule, $\gamma_{n}$. We will now show that $\gamma_{n}$ recovers existing rates for undersmoothed ridge estimators. In particular, we will consider the special case where the hyperparameter schedule $\delta_{n}=\lambda_{n}$ satisfies the conditions for Theorem 1 and Assumption 2 of Singh (2024). This is a nontrivial assumption—i.e. that the smoothness of the Riesz representer RKHS matches that of the outcome model—but the idea is motivated by the concept of the ‘minimal’ Riesz representer from Lemma S3.1 in Chernozhukov, Escanciano, et al. (2022). For two functions of *n*, $f_{n}$ and $g_{n}$, let $f_{n}≍g_{n}$ denote that $f_{n}=O(g_{n})$ and $g_{n}=O(f_{n})$. The resulting augmented hyperparameter is then $\gamma_{n}≍\lambda_{n}^{2}$.

When the RKHS is finite dimensional, the choice $\lambda_{n}=\delta_{n}=n^{-1/2}$ is optimal for controlling the prediction error for both the outcome and weighting models (Caponnetto & De Vito, 2007; Singh, 2024). The augmented estimator is then equivalent to a single ridge regression with hyperparameter $\gamma_{n}≍n^{-1}$, which recovers the rate of Hirshberg et al. (2019) and Mou et al. (2023).

When the RKHS is infinite-dimensional, when $\lambda_{n}=\delta_{n}=n^{-1/2}$, then $\gamma_{n}≍n^{-1}$, again matching the rate in Hirshberg et al. (2019) and Mou et al. (2023). This provides further motivation to fix $\delta_{n}=\lambda_{n}$. However, depending on the smoothness and effective dimension of the RKHS, $\lambda_{n}$ can take on a range of values, resulting possibly faster or slower rates than $n^{-1}$; we give concrete examples in the online supplementary material, Appendix. This somewhat contrasts with the results in Hirshberg et al. (2019) and Mou et al. (2023), and might be an interesting direction for future analysis. Inspired by these results, in the next section, we will assess the performance of setting δ=λ for hyperparameter tuning in practice. In fully generality, when $\lambda_{n}$ and $\delta_{n}$ differ, we will end up with a product rate, again contrasting with existing work. In this sense, Proposition 4.3 generalizes the standard undersmoothing arguments, which typically change the regularization schedule from $n^{-1/2}$ to $n^{-1}$.

Remark 8 **Single-model double robustness**

Another interesting implication of the equivalence of these two procedures is that the single kernel ridge procedure is doubly robust, much the same way OLS is. Because estimating the coefficients from an OLS regression of *Y* onto features of (Z,X) is equivalent to a balancing weights or an IPW estimator based on a model for the inverse weights that is linear in the same features, this procedure is consistent whenever *either* the weights or the outcome model is truly linear—that is, whenever either of these two linear models is correctly specified (J. Robins et al., 2007). Similarly, the single kernel ridge procedure is DR in that it is consistent if either the true outcome regression or the inverse propensity score is consistently estimated. However, valid inference in the case where the inverse weight model but *not* the outcome model is truly linear will typically require different tuning parameter selection.

### 6.2 Finite sample mean-squared error

We now use our numerical equivalences to write out the exact finite-sample mean squared error (MSE) of the augmented kernel ridge estimator: by rewriting the augmented balancing weights estimator as a single outcome model, we can immediately leverage existing results from Dobriban and Wager (2018).

Following their set-up, we define the diagonal matrix $\hat{\Sigma }\;:=\frac{1}{n}\Phi_{p}^{⊤}\Phi_{p}$; if $\hat{\Sigma }$ is not diagonal, we can apply the rotation in online supplementary material, Appendix E.2. We consider ridge regression with rescaled hyperparameter *λ* and solution $(\hat{\Sigma }+\lambda I{)}^{-1}\Phi_{p}Y_{p}/n$; this is equivalent to standard ridge regression above with hyperparameter nλ, and also accommodates kernel ridge regression with appropriate choice of $\Phi_{p}$. Assume that $Y_{p}=\Phi_{p}\beta_{0}+\epsilon$ with $\beta_{0}\in R^{d}$, and where $\epsilon \in R^{n}$ are iid with mean zero and variance $\sigma^{2}$. Then the exact, design-conditional, squared bias and variance of the ridge regression prediction applied to a new iid sample $(\Phi_{\mathrm{new}},Y_{\mathrm{new}})∼p$ are:


$$\begin{array}{rl} B_{p}^{2}(\lambda ) & =\lambda^{2}\beta_{0}^{⊤}(\hat{\Sigma }+\lambda I{)}^{-1}E[\Phi_{p}^{⊤}\Phi_{p}](\hat{\Sigma }+\lambda I{)}^{-1}\beta_{0}\\ V_{p}(\lambda ) & =\frac{\sigma^{2}}{n}\text{tr}\left[\hat{\Sigma }(\hat{\Sigma }+\lambda I{)}^{-1}E[\Phi_{p}^{⊤}\Phi_{p}](\hat{\Sigma }+\lambda I{)}^{-1}\right]. \end{array}$$

Applying Proposition 4.3, we can simlarly derive the squared bias and variance of an augmented ridge estimator for our linear functional estimand; we denote these quantities $B_{q}^{2}$ and $V_{q}$ respectively. We express the bias and variance in terms of the two hyperparameters, *λ* and *δ*:

Proposition 6.1 Let $\sigma_{j}^{2}$ denote the eigenvalues of $\hat{\Sigma }$ and define $\Gamma_{\lambda ,\delta }$ to be the diagonal matrix with nonzero entries $\gamma_{j}\;:=\frac{\delta \lambda }{\sigma_{j}^{2}+\delta +\lambda }$. Then,

$$\begin{array}{rl} B_{q}^{2}(\lambda ,\delta ) & =\beta_{0}^{⊤}(\hat{\Sigma }+\Gamma_{\lambda ,\delta }{)}^{-1}\Gamma_{\lambda ,\delta }E[\Phi_{q}{]}^{⊤}E[\Phi_{q}]\Gamma_{\lambda ,\delta }(\hat{\Sigma }+\Gamma_{\lambda ,\delta }{)}^{-1}\beta_{0}\\ V_{q}(\lambda ,\delta ) & =\frac{\sigma^{2}}{n}\text{tr}\left[\hat{\Sigma }(\hat{\Sigma }+\Gamma_{\lambda ,\delta }{)}^{-1}E[\Phi_{q}{]}^{⊤}E[\Phi_{q}](\hat{\Sigma }+\Gamma_{\lambda ,\delta }{)}^{-1}\right]. \end{array}$$

In the next section, we compare—numerically and via simulation—existing hyperparameter selection schemes to the optimal trade-off between $B_{q}^{2}$ and $V_{q}$. However, first we note that the analysis above opens up exciting new avenues for both theoretical and methodological work. One could theoretically analyse the mean squared error to understand how the optimal *δ* scales with the problem parameters; for example, by using proportionate asymptotics from random matrix theory as in the high-dimensional ridge regression literature (Hastie et al., 2022; Patil et al., 2024). Furthermore, while Proposition 6.1 requires the linear model to be well specified, in the mis-specified setting, we could adapt the model-agnostic decomposition from Proposition 1 of Patil et al. (2024). Finally, our analysis here suggests a novel, hyperparameter selection scheme based on plugging in the unknown quantities in Proposition 6.1. We leave this to future work.

## 7 Numerical illustrations and hyperparameter tuning

This section illustrates our results in practice. We first explore hyperparameter tuning for double ridge regression, comparing practical methods to the optimal hyperparameter computed using our results from Proposition 6.1. Following our asymptotic results in Section 6.1, we recommend equating the weighting and outcome model hyperparameters in practice. We then apply both double ridge and lasso-augmented $\ell_{\infty }$-balancing to two versions of the canonical (LaLonde, 1986) application. An important theme throughout is that some approaches for hyperparameter selection can choose δ=0, which collapses the augmented estimate to OLS alone—even in settings where this is far from optimal. Overall, we take this as a warning that existing hyperparameter tuning schemes can be potentially misleading when applied naively.

### 7.1 Hyperparameter tuning for ridge-augmented $\ell_{2}$ balancing

We begin with practical hyperparameter tuning for the special case of double ridge, building on the MSE expression in Section 6.2. There is an active literature on selecting hyperparameters for augmented balancing weights estimators and DML estimators more broadly (Bach et al., 2024; Ben-Michael, Feller, Hirshberg, et al., 2021; Kallus, 2020; Wang & Zubizarreta, 2020). We contribute to this literature by comparing practical hyperparameter tuning schemes based on cross-validation (CV) with an oracle hyperparameter tuning scheme based on Proposition 6.1.

Reflecting empirical practice, we focus here on choosing hyperparameters sequentially: we first select the outcome model hyperparameter *λ* (e.g. by cross-validation) and then select the weighting model hyperparameter *δ*. Ultimately, we find strong performance for both *CV imbalance* and *CV outcome* hyperparameters, as defined below. We especially recommend the latter as a reasonable starting point in practice. In addition to theoretical support from our asymptotic analysis, the outcome model hyperparameter scheme does not require any additional algorithm or code after having fit the initial outcome model.

#### 7.1.1 Oracle and practical hyperparameter tuning

##### Oracle hyperparameter

To compute oracle hyperparameters, we first compute the prediction-MSE-optimal *λ* using the standard ridge regression MSE expression, and then we use Proposition 6.1 to compute the corresponding optimal *δ* for the linear functional estimand:


$$\begin{array}{rl} \lambda^{*} & \;:={\text{argmax}}_{\lambda }\{B_{p}^{2}(\lambda )+V_{p}(\lambda )\}\\ \delta^{*} & \;:={\text{argmax}}_{\delta }\{B_{q}^{2}(\lambda^{*},\delta )+V_{q}(\lambda^{*},\delta )\}. \end{array}$$

While there is not a closed form for $\delta^{*}$, we can nonetheless directly compute this optimal hyperparameter and characterize its behaviour under a range of scenarios. We draw several conclusions about optimal $\delta^{*}$ for a wide range of DGPs of the form $Y_{p}=\Phi_{p}\beta_{0}+\epsilon$. First, $\delta^{*}$ is generally increasing in the noise, $\sigma^{2}$: larger $\sigma^{2}$ typically implies larger $\delta^{*}$. Second, $\delta^{*}$ generally depends on the target mean, $E[\Phi_{q}]$; that is, two DGPs that are identical except for $E[\Phi_{q}]$ can have different values of $\delta^{*}$. The optimal hyperparameter, however, does *not* depend on the magnitude of the shift in the target mean: replacing $E[\Phi_{q}]$ with $cE[\Phi_{q}]$ for c≠0, scales both the bias and variance by $c^{2}$, leaving $\delta^{*}$ unchanged.

##### Practical hyperparameter

We compare the oracle hyperparameter with three implementable practical proposals. In all cases, we first pick *λ* by cross-validating the mean squared error of a ridge outcome model.

- *CV imbalance.* Choose *δ* by cross-validating the estimated imbalance, $\|\frac{1}{n}\hat{w}\Phi_{p}-{\bar{\Phi }}_{q}{\|}_{2}^{2}$ , adapting a proposal from Wang and Zubizarreta (2020).
- *CV Riesz loss.* Choose *δ* by cross-validating the Riesz loss in Equation (6), adapting a proposal from Chernozhukov et al. (2022a); this is the dual form of cross-validating the estimated imbalance.
- *CV outcome.* Choose *δ* to be equal to the cross-validated ridge outcome *λ*, as inspired by the asymptotic theory in Mou et al. (2023) and Singh (2024).

Before presenting simulation results, we provide a preliminary analytic discussion, comparing these practical schemes to the behaviour of the oracle $\delta^{*}$. For the first two proposals: just like the oracle, both depend on the target mean $E[\Phi_{q}]$ and are invariant to rescaling. However, these two approaches are mechanically independent of the outcomes $Y_{p}$, unlike the oracle $\delta^{*}$ which, in general, depends on the variance of the outcomes. By constrast, the last proposal depends on the outcomes $Y_{p}$ but is mechanically independent of $E[\Phi_{q}]$.

This suggests that any one of these tuning parameter approaches cannot perform well across all DGPs. In future work, if we pursue a theoretical analysis of the oracle hyperparameter, e.g. in a proportionate asymptotics framework, we may be able predict when either the outcomes or the covariate shift is more important. In this work, we begin by demonstrating that no one tuning scheme does uniformly best in simulations.

#### 7.1.2 Simulation study

To assess the behaviour of these hyperparameter tuning schemes, we conduct a simulation study using 36 distinct DGPs: 30 synthetic and 6 semi-synthetic; see online supplementary material, Appendix H for a detailed discussion. For each DGP, we directly compute the oracle hyperparameter using the results in Section 6.2. We then compute values from the three practical hyperparameter tuning methods discussed above. The mean squared error that we consider is design-conditional, and so we draw samples of the covariates for each DGP only once.

Table 1 presents a summary of the MSE for the three methods across the 36 DGPs. Overall, we find that the *CV outcome* approach of choosing δ=λ and the *CV imbalance* approach both perform well in practice: one of these two achieves the lowest MSE all 36 DGPs, with CV imbalance performing slightly better on average. By contrast, selecting *δ* via CV for the Riesz loss has numerical stability problems that compromises performance. The performance for the *outcome* and *balance* approaches, on the other hand, seem to degrade gracefully and rarely perform catastrophically. Taken together, these preliminary findings suggest researchers should begin with these two tuning methods as defaults.

**Table 1.** Mean-squared error (relative to the oracle) for three hyperparameter selection methods for *double ridge regression* from a numerical investigation of 36 DGPs (30 synthetic and 6 semi-synthetic)

	No. of DGPs		Relative MSE			
Method	Best	Worst	Median	Best	Worst	Prop.(δ=0)
CV outcome	9	2	0.57	0.096	$2\times 10^{5}$	0
CV imbalance	27	2	0.41	0.043	$2\times 10^{5}$	0
CV Riesz Loss	0	32	9,268	0.330	$3\times 10^{7}$	0.56

*Note.* The final column is the proportion of draws where the hyperparameter δ=0.

##### Recovering the OLS point estimate

As we discuss above (see, e.g. Figure 1), when δ=0 the point estimate for the augmented balancing weights estimator is numerically identical to the OLS point estimate. Thus, when a hyperparameter tuning procedure chooses δ=0 in practice, researchers are simply estimating the equivalent of OLS—even if they are unaware they are doing so. This is especially problematic in settings where OLS is far from optimal (though see Hastie et al., 2022; Kobak et al., 2020, for counterexamples). In our synthetic and semi-synthetic DGPs, δ=0 is never optimal, and is usually associated with a very large error driven by extreme variance—see for example, online supplementary material, Figure I.11 in the Appendix. Thus, the fact that hyperparameter tuning procedures can return δ=0 in these DGPs represents a pathological case.

In our simulation study, we find that, when cross validating the Riesz loss, over half of all draws returned δ=0. By contrast, none of the other methods returned δ=0 in the synthetic DGPs, though, as we discuss below, we do observe exact zeros for *δ* occasionally when cross-validating imbalance in the standard LaLonde dataset. This highlights the potential numeric instability of hyperparameter tuning via CV for the Riesz loss, at least in the settings we consider here. We further suggest that in these cases, practitioners assess the sensitivity of the δ=0 results to the particular tuning procedure used or to the random choice of cross-validation splits.

### 7.2 Application to LaLonde (1986)

We now illustrate our equivalence and hyperparameter tuning results on real-world datasets. Following Chernozhukov et al. (2022a), we focus on the canonical (LaLonde, 1986) dataset evaluating a job training program in the National Supported Work (NSW) Demonstration. The primary outcome of interest is annual earnings in 1,978 dollars.

For these illustrations, we estimate the Average Treatment Effect on the Treated (ATT), E[Y(1)−Y(0)∣Z=1]. We recover the missing conditional mean E[Y(0)∣Z=1] using the set-up from online supplementary material, Example 3 in Appendix A, where the source and target populations are the control and treated units, respectively. Thus $\Phi_{p}$ and $\Phi_{q}$ correspond to the feature expansion ϕ(X) applied to the covariates in the control group and treated group, respectively. We consider two different features expansions of the original covariates: (1) a ‘short’ set of 11 covariates used in Dehejia and Wahba (1999)^1^ ; and (2) an expanded, ‘long’ set of 171 interacted features used in Farrell (2015).

Our goal is to explicate how augmented estimators under different hyperparameter tuning schemes undersmooth in practice in both low- and high-dimensional settings. In some cases, the augmented estimator collapses to exactly OLS as we document above. Online supplementary material, Appendix I contains extensive additional analyses, including dataset summaries, additional results from the Infant Health Development Program (IHDP), and sensitivity of these numerical results to cross-fitting.

#### 7.2.1 High-dimensional setting

**Figure 3.**
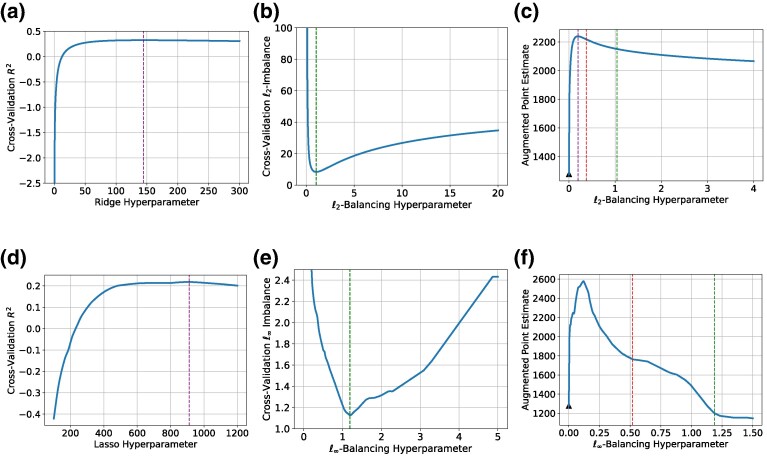
Augmented balancing weights estimates for the LaLonde (1986) dataset with the expanded set of 171 features used in Farrell (2015); the top row shows ridge-augmented $\ell_{2}$ balancing, and the bottom row shows lasso-augmented $\ell_{\infty }$ balancing. Panels (a) and (d) show the threefold cross-validated $R^{2}$ for the ridge- and lasso-penalized regression of $Y_{p}$ on $\Phi_{p}$ among control units across the hyperparameter *λ*; the dotted lines show the CV-optimal value for each. Panel (b) and (e) show the threefold cross-validated imbalance for $\ell_{2}$ and $\ell_{\infty }$ balancing weights across the hyperparameter *δ*; the dotted lines show the CV-optimal value for each. Panels (c) and (f) show the point estimates for the augmented estimators across the weighting hyperparameter *δ*; the triangles correspond to the OLS point estimate; the dotted lines in (c) from left to right correspond to the cross-validated ridge hyperparameter (for $\delta =\hat{\lambda })$, Riesz loss, and balance respectively; likewise, the dotted lines in (f) from left to right correspond to the cross-validated Riesz loss and balance respectively. (a) Ridge outcome model, (b) $\ell_{2}$ balancing, (c) estimate from ‘double ridge’, (d) Lasso outcome model, (e) $\ell_{\infty }$ balancing and (f) estimate from ‘double lasso’.

Following Chernozhukov et al. (2022a), we first consider the expanded set of 171 features for LaLonde (1986) used in Farrell (2015). Figure 3 shows estimates for ridge-augmented $\ell_{2}$ balancing (top row) and lasso-augmented $\ell_{\infty }$ balancing (bottom row). We explicitly characterize these results in terms of undersmoothing in online supplementary material, Appendix I.4. The left two panels of each row show the cross-validation curves for the outcome regression and balancing weights, respectively. The right panels show the point estimate as a function of the weighting hyperparamter *δ*, holding the outcome model hyperparameter *λ* fixed; the black triangle represents the OLS plug-in point estimate. For context, the corresponding experimental estimate is $1,794 (see Dehejia & Wahba, 1999). The green and red dotted lines correspond to hyperparameters chosen by cross-validating balance and the Riesz loss, respectively. For the double ridge estimate, the purple line corresponds to $\delta =\hat{\lambda }$, the outcome hyperparameter selected via cross validation.

Figure 3 highlights that both the imbalance and the point estimate are highly nonlinear close to zero. Thus, even small departures from OLS (at δ=0) lead to large changes in the point estimate—in online supplementary material, Appendix I.5 we give some suggestive evidence that the variance blows up relative to the bias in this range. We can also assess the sensitivity of the point estimate to the hyperparameter selection scheme. In this case, choosing *δ* via CV balance leads to meaningfully larger choices than via other methods.

Finally, the selected *δ* is always strictly greater than zero for this high-dimensional dataset. However, we find this is sensitive to small perturbations in the problem parameters. For example, when we perturb $E[\Phi_{q}]$ by adding a small value to all the even elements, then the cross-validated $\ell_{2}$ Riesz loss chooses δ=0 in 38% of draws of the cross-validation splits. As suggested by online supplementary material, Appendix I.5 and our simulation results, this is likely to result in extremely large mean squared error.

#### 7.2.2 Low-dimensional setting: recovering OLS

**Figure 4.**
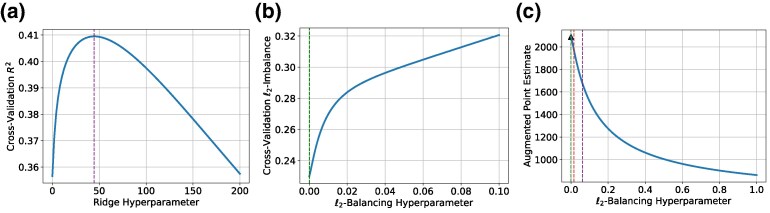
Ridge-augmented $\ell_{2}$ balancing weights (‘double ridge’) for LaLonde (1986) with the original 11 covariates. Panel (a) shows the threefold cross-validated $R^{2}$ for the Ridge-penalized regression of $Y_{p}$ on $\Phi_{p}$ among control units across the hyperparameter *λ*; the dotted line shows the CV-optimal value, $\hat{\lambda }$. Panel (b) shows the threefold cross-validated imbalance for $\ell_{2}$ balancing weights across the hyperparameter *δ*; the dotted line shows the CV-optimal value, which is δ=0 or exact balance. Panel (c) shows the point estimate for the augmented estimator across the weighting hyperparameter *δ*; the triangle corresponds to the OLS point estimate, the dotted lines from left to right correspond to cross-validated balance, Riesz loss, and ridge outcome hyperparameter respectively. (a) Ridge outcome model, (b) $\ell_{2}$ balancing and (c) Augmented estimate.

Finally, we apply double ridge to the ‘short’ version of the LaLonde (1986) dataset with 11 features. Figure 4 shows the cross-validation curves for the outcome and weighting models, as well as the point estimate as a function of the balance hyperparameter, with the OLS estimate given by the black triangle. As above, the green, red, and purple dotted lines correspond to hyperparameters chosen by cross-validating balance, cross-validating the Riesz loss, and choosing δ=λ, respectively.

Unlike for the ‘long’ dataset in Figures 3 and 4 does not display as stark a nonlinearity around zero. Importantly, however, setting *δ* by cross-validating imbalance can yield δ=0, which reduces the augmented estimator to exactly the estimate from a simple OLS regression—even though the base learner ridge outcome model is heavily regularized. By contrast, our preferred hyperparameter tuning scheme of choosing δ=λ results in an estimate that is roughly $400 dollars smaller than the OLS estimate. The choice of δ=0 is sensitive to the specific cross-validation splits used, further emphasizing that this is likely anomalous behaviour. See Section 7.1.2 for further discussion.

## 8 Discussion

We have shown that augmenting a plug-in regression estimator with linear balancing weights results in a new plug-in estimator with coefficients that are shrunk towards—in some cases all the way to—the estimates from OLS fit on the same observations. We generalize this equivalence for different choices of outcome and weighting regressions. In the asymptotic setting, we draw the explicit connection between augmented estimators and undersmoothing for the special case of kernel ridge regression. Then we derive the design-conditional finite sample MSE for the double ridge estimator, and use it to solve numerically for oracle hyperparameters. We compare the oracle hyperparameters with three practical tuning schemes and then illustrate our results on the canonical (LaLonde, 1986) dataset. In the online supplementary material, Appendix, we also explore many extensions, including to nonlinear weights and to high-dimensional features.

There are many promising avenues for future research. The fundamental connection between DR estimation and undersmoothing opens up several theoretical directions. While we focus on the special case of kernel ridge regression in Section 6.1, we anticipate that these connections will hold more broadly. Similarly, while our focus in this paper has been on interpreting balancing weights as a form of linear regression, the converse is also valid: we could instead focus on how many outcome regression-based plug-in estimators are, in fact, a form of balancing weights; see Lin and Han (2022) for connections between outcome modelling and density ratio estimation.

We also anticipate that the MSE we derive in Section 6.2 is a starting place for future theoretical analysis that can inform practice. We demonstrate in our simulation study that existing hyperparameter selection methods cannot perform uniformly well over all DGPs. We expect that analysing the optimal hyperparameters—e.g. in a proportionate asymptotics regime—can help devise new tuning schemes and inform which tuning method will work best on the dataset at hand.

We further conjecture that these results may provide new insights into the estimation of causal effects in the proximal causal inference framework (Tchetgen Tchetgen et al., 2020). This framework uses proxy variables to identify causal effects in the presence of unmeasured confounding. Estimation has been complicated by the fact that, in the absence of strong parametic assumptions, estimators of proximal causal effects are solutions to ill-posed Fredholm integral equations. Ghassami et al. (2022) and Kallus et al. (2021) recently proposed tractable nonparametric estimators in this setting. They use an ‘adversarial’ version of double kernel ridge regression—allowing the weighting and outcome models to have different bases—to estimate the solution to the required Fredholm integral equations. Our results apply immediately to standard augmented estimators with different bases for the outcome and weighting models, either via a union basis (Chernozhukov et al., 2022a) or by applying an appropriate projection as in Hirshberg and Wager (2021), and extending these results to proximal causal effect estimators might help in constructing new proximal balancing weights, matching, or regression estimators with attractive asymptotic properties.

Finally, many common panel data estimators are forms of augmented balancing weight estimation (Abadie et al., 2010; Arkhangelsky et al., 2021; Ben-Michael, Feller, Rothstein, 2021). We plan to use the numeric results here, especially the results for simplex-constrained weights in online supplementary material, Appendix D.2, to better understand connections between methods and to inform inference.

## Supplementary Material

qkaf019_Supplementary_Data

## Data Availability

The data analysed in this paper, including the code used to generate simulated data, is available at https://github.com/bruns-smith/balance-equiv-jrssb.

## Footnotes

1 These are: age, years of education, Black indicator, Hispanic indicator, married indicator, 1974 earnings, 1975 earnings, age squared, years of education squared, 1974 earnings squared, and 1975 earnings squared.
