# Supplementary material for: Augmented balancing weights as linear regression
Source: J R Stat Soc Series B Stat Methodol. 2025 Apr 24;88(3):699–723. doi: 10.1093/jrsssb/qkaf019 (PMC13370344; doi:10.1093/jrsssb/qkaf019)
Supplement: qkaf019_Supplementary_Data [file qkaf019_supplementary_data.pdf]

Supplementary Material for "Augmented balancing weights as linear regression"

Contents

A Additional background and examples **3**
- A.1 Examples of general linear functionals via the Riesz representer . . . 3
- A.2 Examples of balancing weights . . . 3
- A.3 Causal inference . . . 4
- A.4 Counterfactual features for linear functionals . . . 5
- A.5 Equivalences of outcome regression and balancing weighting methods . . . 5

B Details for when $d > n$ **6**
- B.1 Balancing weights when $d > n$ . . . 6
- B.2 Equivalences from Appendix A.5 when $d > n$ . . . 7
- B.3 Propositions 3.1 and 3.2 when $d > n$ . . . 8
- B.4 The RKHS Setting . . . 8

C Sample Splitting and Cross-Fitting **9**
- C.1 Proposition 3.2 with Sample Splitting . . . 10
- C.2 Unregularized Outcome Model . . . 10
- C.3 Unregularized Weight Model . . . 11
- C.4 "Double Ridge" . . . 11

D Nonlinear weights **11**
- D.1 General nonlinear weights . . . 12
  - D.1.1 Illustration: LaLonde . . . 13
- D.2 Constraining weights to be non-negative . . . 13
- D.3 Non-Linear Differentiable Outcome Models . . . 14

E Results with Correlated Features **15**
- E.1 Overview . . . 15
- E.2 Augmented ℓ_2 Balancing Weights with Correlated Features . . . 15
- E.3 Augmented ℓ_∞ balancing weights . . . 18

F Undersmoothing with general weights as viewed by the normal equations **19**

G Comparison to TMLE with Balancing Weights **20**

H Simulation Study Details **21**
- H.1 Setup . . . 21
- H.2 Synthetic DGPs . . . 21
- H.3 Semi-Synthetic DGPs . . . 22

I Additional Numerical Experiments 22
I.1 Dataset Summaries . . . 22
I.2 Numerical Example with cross-fitting . . . 23
I.3 Additional Experiments . . . 23
I.3.1 Low Dimensional . . . 23
I.3.2 High Dimensional . . . 24
I.4 Undersmoothing in LaLonde . . . 24
I.4.1 Undersmoothing for double ridge . . . 24
I.4.2 Undersmoothing in norm . . . 25
I.5 Semi-Synthetic Bias vs Variance . . . 25

J Empirical Bayes Interpretation of Augmented ℓ_2 Balancing Weights 25
J.1 Fixed outcome coefficients . . . 26
J.2 Accounting for uncertainty in outcome model coefficients via global-local shrinkage priors . . 27
J.2.1 Scale mixture of Normals . . . 27
J.2.2 Inverse-Gamma Gamma Prior . . . 29
J.2.3 Application to double ridge . . . 29
J.3 Connection to Zellner's g prior . . . 32

K Additional Proofs 34

L Additional Details for Asymptotic Results 37

A Additional background and examples

A.1 Examples of general linear functionals via the Riesz representer

Example 1 (Counterfactual mean)**.** *Let $Z \in \{0, 1\}$ and $\psi(m) = \mathbb{E}[m(X, 1)]$. Under SUTVA and conditional ignorability, this estimand is equal to $E[Y(1)]$. The Riesz representer is the IPW, $\alpha(X, Z) = Z/e(X)$.*

Example 2 (Average derivative)**.** *Let $Z \in \mathbb{R}$ and $\psi(m) = \mathbb{E}\left[\frac{\partial}{\partial z} m(X, Z)\right]$. Under an appropriate generalization of SUTVA and conditional ignorability, this estimand corresponds to the average derivative effect of a continuous treatment. Under regularity conditions the Riesz representer is given by $\alpha(X, Z) = -\frac{\frac{d}{dz}p(Z|X)}{p(Z|X)}$ where $p(z|x)$ is the conditional density of Z given X.*

Example 3 (Distribution shift)**.** *Consider an example without Z, following the machine learning literature on covariate shift. Let p denote the* source *distribution of the observed data, and let p^* over $\mathcal{X}$ denote the* target *distribution. The estimand is then $\psi(m) = \int_{\mathcal{X}} m(x)dp^*(x)$. In a causal inference setting, this can recover the Average Treatment Effect on the Treated (ATT) under SUTVA and conditional ignorability; i.e., let p be the distribution of covariates and outcomes for units assigned to control and p^* be the distribution of the covariates for units assigned to treatment. The Riesz representer is the density ratio, $\alpha(X) = \frac{dp^*}{dp}(X)$.*

A.2 Examples of balancing weights

Example 4 (Exact balancing weights)**.** *The most common balancing weights estimation problem finds the minimum weights that exactly balance each element of Φ. In the constrained form, exact balancing solves*

$$\begin{aligned} &\min_{w \in \mathbb{R}^n} \|w\|_2^2 \\ &\textit{such that } \tfrac{1}{n} w\phi_{pj} = \bar{\phi}_{qj} \quad \textit{for all } j \end{aligned} \tag{1}$$

Example 5 (ℓ_2 balancing)**.** *The ℓ_2 balancing weights problem is usually expressed via its penalized form:*

$$\min_{w \in \mathbb{R}^n} \left\{ \|\tfrac{1}{n} w\Phi_p - \overline{\Phi}_q\|_2^2 + \delta\|w\|_2^2 \right\}. \tag{2}$$

The automatic form is a ridge-penalized regression for the Riesz representer.

Example 6 (ℓ_∞ balancing)**.** *The constrained form of the ℓ_∞ balancing weights problem is*

$$\begin{aligned} &\min_{w \in \mathbb{R}^n} \|w\|_2^2 \\ &\textit{such that } \|\tfrac{1}{n} w\Phi_p - \overline{\Phi}_q\|_\infty \le \delta \end{aligned} \tag{3}$$

The automatic form is a lasso-penalized regression for the Riesz representer, sometimes known as the Minimum Distance Lasso *(Chernozhukov et al., 2022b).*

Example 7 (Kernel balancing)**.** *As a brief preview of the balancing problem in the infinite-dimensional setting, we provide an example where $\mathcal{F} = \mathcal{H}$ is a reproducing kernel Hilbert space on $\mathcal{X} \times \mathcal{Z}$ with norm $\|\cdot\|_{\mathcal{H}}$ and kernel $\mathcal{K} : (\mathcal{X} \times \mathcal{Z}) \times (\mathcal{X} \times \mathcal{Z}) \to \mathbb{R}$. Then for any $x_i \in \mathcal{X}$, $z_i \in \mathcal{Z}$, the features $\phi(x_i, z_i) := \mathcal{K}(x_i, z_i, \cdot, \cdot) \in \mathcal{H}$. Using infinite-dimensional matrix notation, we denote $\Phi_p \in \mathcal{H}^n$ and $\overline{\Phi}_q \in \mathcal{H}$ as above. The penalized balancing weights problem for $\mathcal{F} = \mathcal{H}$ is:*

$$\min_{w \in \mathbb{R}^n} \left\{ \|\tfrac{1}{n} w\Phi_p - \overline{\Phi}_q\|_{\mathcal{H}}^2 + \delta\|w\|_2^2 \right\}. \tag{4}$$

See Appendix B for details and references.

A.3 Causal inference

We now return to Example 1 above. Here the goal is estimating the unobserved potential outcomes in an observational study. Let Y be the potential outcome under control (with appropriate restrictions, such as SUTVA; Rubin, 1980), let p be the population of individuals in the control condition, and let q be the population of individuals in the treatment condition. Then Y is observed for population p but not for population q, and the missing mean, $\mathbb{E}_q[Y]$, is the average potential outcome under control for the individuals who in fact were treated. Letting Y be the potential outcome under treatment, p the population of individuals in the treatment condition, and q the population of individuals in the control condition, $\mathbb{E}_q[Y]$ is the average potential outcome under treatment for the individuals who in fact received control.

For both examples, the crucial assumption for identification is *conditional ignorability*: the conditional distribution of Y given X is the same in the source and target populations. This is also known as "conditional exchangeability," "selection on observables," or "no unmeasured confounding." For our purposes, we will require the mean, but not distributional, version of this assumption: $\mathbb{E}_p[Y|X] = \mathbb{E}_q[Y|X]$.

Since we assume the conditional expectations are the same in the two populations, we occasionally denote the common conditional mean functional without subscripts, $\mathbb{E}[Y|X]$. Under this assumption, we can identify $\mathbb{E}_q[Y]$ with the *regression functional*, also known as the *adjustment formula* or *g-formula*:

$$\mathbb{E}_q\left[\mathbb{E}_p[Y|X]\right] = \mathbb{E}_q\left[\mathbb{E}_q[Y|X]\right] = \mathbb{E}_q[Y]. \tag{5}$$

A complementary approach instead relies on the density ratio between the marginal covariate distributions in the source and target populations, $\frac{dq}{dp}(X)$, also known as the Radon-Nikodym derivative, importance sampling weights, or inverse propensity score weights (IPW). [1] This is also a special case of a Riesz representer (Chernozhukov et al., 2022b). Under an additional *population overlap assumption* that $q(x)$ is absolutely continuous with respect to $p(x)$, we can identify $\mathbb{E}_q[Y]$ via the *weighting functional*, also known as the *IPW functional*:

$$\mathbb{E}_p\left[\frac{dq}{dp}(X)\, Y\right] = \mathbb{E}_p\left[\frac{dq}{dp}(X)\, \mathbb{E}_p[Y|X]\right] = \mathbb{E}_q[\mathbb{E}_p[Y|X]] = \mathbb{E}_q[Y]. \tag{6}$$

Finally, we can combine the regression and weighting functionals to create a third identifying functional, known as the *doubly robust functional* (Robins et al., 1994):

$$\mathbb{E}_q\left[\mathbb{E}_p[Y|X]\right] + \mathbb{E}_p\left[\frac{dq}{dp}(X)\left\{Y - \mathbb{E}_p[Y|X]\right\}\right]. \tag{7}$$

This functional has the attractive property of being equal to $\mathbb{E}_q[Y]$ even if either one of $\frac{dq}{dp}(X)$ or $\mathbb{E}_p[Y|X]$ is replaced with an arbitrary function of X, hence the term "doubly robust."[2] See Chernozhukov et al. (2018); Kennedy (2022) for recent overviews of the active literature in causal inference and machine learning focused on estimating versions of Equation (3).

The *augmented* estimators that we analyze in this paper are based on estimating this doubly robust functional. These estimators *augment* an estimator of the regression functional based on an outcome regression (or *base learner*) with appropriately weighted residuals. Alternatively, they *augment* an estimator of the weighting functional with an outcome regression-based estimator of the regression functional (subtracting off the implied estimator of $\frac{dq}{dp}(X)\mathbb{E}_p[Y|X]$).

[1] Using Bayes Rule, we can equivalently express $\frac{dq}{dp}(X)$ via the *propensity score* $P(1_p|X)$, where 1_p is the indicator that an observation from the size-proportional mixture distribution of p and q is from population p: $\frac{dq}{dp}(X) = \frac{1-P(1_p|X)}{P(1_p|X)}\frac{P(1_p)}{1-P(1_p)}$

[2] This functional is equal to $\mathbb{E}_q[Y]$ if $\mathbb{E}_p[Y|X]$ is replaced with an arbitrary well-behaved functional of X_p, because the first and last terms cancel and we are left with the weighting functional $\mathbb{E}_p[\frac{dq}{dp}(X)Y]$. It is also equal to $\mathbb{E}_q[Y]$ if $\frac{dq}{dp}(X)$ is replaced with an arbitrary well-behaved functional of X_p, because the $\mathbb{E}_p[h(X)(Y - \mathbb{E}_p[Y|X])]$ is equal to 0 for any h and therefore we are left with the regression functional $\mathbb{E}_q\left[\mathbb{E}_p[Y|X]\right]$.

A.4 Counterfactual features for linear functionals

Recall that under linearity the imbalance over all $f \in \mathcal{F}$ has a simple closed form. For any $f(x,z) = \theta^\top \phi(x,z) \in \mathcal{F}$, $\mathbb{E}[h(X,Z,f)] = \theta^\top \mathbb{E}[h(X,Z,\phi)]$, where $h(X,Z,\phi)$ is short-hand for the vector with jth entry $h(X,Z,\phi_j)$. We can then write the imbalance in terms of a transformed feature space $h(X, Z\phi)$, giving a closed form that we can readily calculate by applying the linear functional ψ from (1) to the features ϕ :

$$\begin{aligned}\text{Imbalance}_{\mathcal{F}}(w) &:= \sup_{f\in\mathcal{F}} \Big\{\mathbb{E}[w(X,Z)f(X,Z)] - \mathbb{E}[h(X,Z,f)]\Big\} \\ &= \sup_{\|\theta\|\le 1} \Big\{\theta^\top \mathbb{E}[w(X,Z)\phi(X,Z)] - \theta^\top \mathbb{E}[h(X,Z,\phi)]\Big\} \\ &= \big\|\mathbb{E}[w(X,Z)\phi(X,Z)] - \mathbb{E}[h(X,Z,\phi)]\big\|_*.\end{aligned}$$

Consider the counterfactual mean estimand $\psi(m) = \mathbb{E}[m(X,0)|Z=1]$. We have

$$\text{Imbalance}_{\mathcal{F}}(w) = \big\|\mathbb{E}[w(X,Z)\phi(X,Z)] - \mathbb{E}[\phi(X,0)|Z=1]\big\|_*.$$

For a very simple example, let $\phi(x,z) = x$. Now we get $\text{Imbalance}_{\mathcal{F}}(w) = |\mathbb{E}[w(X,Z)X] - \mathbb{E}[X|Z=1]|$ and therefore, the balancing optimization problem finds weights w that reweight the total mean $\mathbb{E}[X]$ to approximate the conditional mean $\mathbb{E}[X|Z=1]$.

A.5 Equivalences of outcome regression and balancing weighting methods

For the special case of ℓ_2 kernel balancing, the balancing weights problem is numerically equivalent to directly estimating the conditional expectation $\mathbb{E}[Y_p|\Phi_p]$ via kernel ridge regression and applying the estimated coefficients to $\bar{\Phi}_q$. We begin with the special case of unregularized linear regression and then present the more general setting. We initially present the results assuming $d < n$ and that Φ_p has rank d, turning to the high-dimensional case with $d > n$ in Appendix B.

Linear regression. Ordinary least squares regression is equivalent to a weighting estimator that exactly balances the feature means. See Fuller (2002) for discussion in the survey sampling literature; see Robins et al. (2007), Abadie et al. (2010), Kline (2011), and Chattopadhyay et al. (2020) for relevant discussions in the causal inference literature.

In particular, let $\hat{w}_{\text{exact}}$ be the solution to the the exact balancing weights problem in Example 4 in the main text. Let $\hat{\beta}_{\text{ols}} = (\Phi_p^\top \Phi_p)^{-1}\Phi_p^\top Y_p$ be the OLS coefficients from the regression of Y_p on Φ_p. We then have the following numerical equivalence:

$$\begin{aligned}\hat{\mathbb{E}}[\Phi_q \hat{\beta}_{\text{ols}}] &= \hat{\mathbb{E}}[\hat{w}_{\text{exact}} \circ Y_p] \\ \bar{\Phi}_q \underbrace{(\Phi_p^\top \Phi_p)^{-1}\Phi_p^\top Y_p}_{\hat{\beta}_{\text{ols}}} &= \underbrace{\bar{\Phi}_q (\Phi_p^\top \Phi_p)^{-1}\Phi_p^\top}_{\frac{1}{n}\hat{w}_{\text{exact}}} Y_p,\end{aligned} \tag{8}$$

where the weights have the closed form $\frac{1}{n}\hat{w}_{\text{exact}} = \bar{\Phi}_q(\Phi_p^\top \Phi_p)^{-1}\Phi_p^\top$.

Ridge regression. This equivalence immediately extends to ridge regression (Speckman, 1979; Hirshberg et al., 2019; Kallus, 2020).[3] Let $\hat{w}^\delta_{\ell_2}$ be the minimizer of the ℓ_2 balancing weights problem in Example 5 in the main text, with hyperparameter δ. Let

$$\hat{\beta}^\delta_{\text{ridge}} := \underset{\beta \in \mathbb{R}^d}{\text{argmin}} \Big\{\|Y_p - \Phi_p \beta\|_2^2 + \delta \|\beta\|_2^2\Big\} \tag{9}$$

[3] See Harshaw et al. (2019) for an interesting connection of this equivalence to experimental design. See Ben-Michael et al. (2021b) and Shen et al. (2022) for related applications in the panel data setting.

be the ridge regression coefficients from least squares regression of Y_p on Φ_p. We then have the following numerical equivalence:

$$\begin{aligned} \hat{\mathbb{E}}[\Phi_q \hat{\beta}^{\delta}_{\text{ridge}}] &= \hat{\mathbb{E}}[\hat{w}^{\delta}_{\ell_2} \circ Y_p] \\ \overline{\Phi}_q \underbrace{(\Phi_p^\top \Phi_p + \delta I)^{-1} \Phi_p^\top Y_p}_{\hat{\beta}^{\delta}_{\text{ridge}}} &= \underbrace{\overline{\Phi}_q (\Phi_p^\top \Phi_p + \delta I)^{-1} \Phi_p^\top}_{\frac{1}{n}\hat{w}^{\delta}_{\ell_2}} Y_p, \end{aligned} \tag{10}$$

where the weights have the closed form $\frac{1}{n}\hat{w}^{\delta}_{\ell_2} = \overline{\Phi}_q(\Phi_p^\top \Phi_p + \delta I)^{-1}\Phi_p^\top$. Thus, the estimate from ridge regression is identical to the estimate using the ℓ_2 balancing weights. We leverage this equivalence in Section 3 below.

Kernel ridge regression. In general, the same equivalence holds in the non-parametric setting where ϕ is the feature map induced by an RKHS. In particular, let $\mathcal{F} = \{f \in \mathcal{H} : \|f\|_{\mathcal{H}} \leq r\}$, where $\mathcal{H}$ is a reproducing kernel Hilbert space (RKHS) on $\mathcal{X} \times \mathcal{Z}$ with kernel $\mathcal{K}$, $\|\cdot\|_{\mathcal{H}}$ denotes the norm of the RKHS, and $r > 0$. Then the equivalence above holds for $\phi(x, z) \coloneqq \mathcal{K}(x, z, \cdot, \cdot)$. Although ϕ is typically infinite-dimensional, the Riesz Representtation Theorem implies that the least squares regression and, equivalently, the balancing optimization problem have closed-form solutions. The least squares regression approach is *kernel ridge regression* and the weighting estimator is *kernel balancing weights* (see Hazlett, 2020; Kim et al., 2022). Hirshberg et al. (2019) leverage this equivalence to analyze the asymptotic bias of kernel balancing weights. For further discussion of this equivalence see Gretton et al. (2012); Kallus (2020).

Finally, we briefly mention some additional papers that discuss relevant equivalences. In the context of panel data, Shen et al. (2022) establish connections between different forms of regression, which is especially relevant for our discussion of high-dimensional features in Appendix B. In addition, Lin and Han (2022) provide an interesting alternative perspective by demonstrating that a large class of outcome regression estimators can be viewed as implicitly estimating the density ratio of the covariate distributions in the two treatment groups. Our results generalize and unify many of these existing numeric equivalences.

B Details for when $d > n$

In this section, we extend our results to the high-dimensional setting. In all that follows we will assume that $d > n$ and we will assume $\Phi_p^\top$ has rank n.[4] For $d = \infty$, we replace $\mathbb{R}^d$ with any infinite-dimensional Hilbert space $\mathcal{H}$ and we require the norm defining $\mathcal{F}$ to be the norm of the Hilbert space. In this case, it should be understood that $\Phi_p \in \mathcal{H}^n$.

B.1 Balancing weights when $d > n$

In the main text, recall that there are three equivalent versions of the balancing weights problem: the penalized, constrained, and automatic form with hyperparameters $\delta_1, \delta_2, \delta_3 \geq 0$ respectively. When $\Phi_p^\top \Phi_p$ is no longer invertible, a unique solution may fail to exist for certain values of these hyperparameters. We provide the relevant technical caveats here.

We begin by mentioning that for $\delta_1 > 0$, the penalized form of the balancing weights optimization problem is strictly convex, and therefore a unique solution exists, regardless of whether $d > n$. However, when $\delta_1 = 0$, there could potentially be infinite many solutions. In this setting, we choose the one with the minimum

[4] Alternatively, we could follow Bartlett et al. (2020) and assume that, almost surely, the projection of Φ_p on the space orthogonal to any eigenvector of $\mathbb{E}[\Phi_p \Phi_p^\top]$ spans a space of dimension n. But as our results are numerical this has no real advantage.

norm:

$$\min_{w \in \mathbb{R}^n} \|w\|_2^2 \quad \text{such that } \|w\Phi_p - \overline{\Phi}_q\|_*^2 = \min_v \|v\Phi_p - \overline{\Phi}_q\|_*^2. \tag{11}$$

If we define $\delta_{\min} := \min_v \|v\Phi_p - \overline{\Phi}_q\|_*^2$, we see that the minimum norm solution in Equation (11) corresponds to a solution to the constrained form of balancing weights with $\delta_2 = \delta_{\min}$. Importantly, no solution exists for $\delta_2 < \delta_{\min}$, and we must make the additional restriction that $\delta_2 \geq \delta_{\min}$. In particular, no solution exists for $\delta_2 = 0$ and we cannot achieve exact balance; that is, for all w, $w\Phi_p \neq \bar{\Phi}_q$.

As in the penalized form, the automatic form is strictly convex and a unique solution exists for $\delta_3 > 0$. When $\delta_3 = 0$ we choose the minimum norm solution: by duality this will be equivalent to the minimum norm solution to the penalized problem (see Bruns-Smith and Feller, 2022).

Note that for $d = \infty$, each "row" of Φ_p is a vector in a Hilbert space $\mathcal{H}$. To solve the balancing weights problem computationally, we need a closed-form solution to the Hilbert space norm $\|\cdot\|_{\mathcal{H}}$. For example, this is a tractable computation when $\mathcal{H}$ is an RKHS.

Singh (2024) gives the automatic form of this problem. See also Wong and Chan (2018), Hazlett (2020), Kallus (2020), and Singh et al. (2020).

B.2 Equivalences from Appendix A.5 when $d > n$

We now extend the equivalences from Appendix A.5 to the high-dimensional case. Let $\delta \geq 0$ be the hyperparameter for the penalized form of balancing weights — as we note above, this is important to state explicitly, since the constrained form will not have a solution for all values of its hyperparameter. For hyperparameter $\delta > 0$, the solutions to ℓ_2 balancing weights and ridge regression are identical as in Equation (9) with no alterations; ridge regression works by default when $d > n$. On the other hand, when $\delta = 0$, there exist infinitely many solutions to the normal equations that define the solution to the OLS optimization problem. Since $(\Phi_p^\top \Phi_p)$ is not invertible, Equation (9) does not apply directly. Instead, we introduce the minimum norm solution to OLS:

$$\min_{\beta \in \mathbb{R}^d} \|\beta\|_2^2 \quad \text{such that } \|\Phi_p\beta - Y_p\|_2^2 = \min_{\beta'} \|\Phi_p\beta' - Y_p\|_2^2.$$

See Bartlett et al. (2020) for an extensive discussion of this optimization problem and its statistical properties as an OLS estimator. For $d > n$, the minimum norm solution is:

$$\hat{\beta}_{\text{ols}} := (\Phi_p^\top \Phi_p)^\dagger \Phi_p^\top Y_p = \Phi_p^\top (\Phi_p \Phi_p^\top)^{-1} Y_p,$$

where $A^\dagger$ denotes the pseudoinverse of a matrix A. Note that the definition holds in general.[5] The second equality holds only when $d > n$. In the low-dimensional setting in the main text, $(\Phi_p^\top \Phi_p)$ is invertible, and so $(\Phi_p^\top \Phi_p)^\dagger = (\Phi_p^\top \Phi_p)^{-1}$.

A version of Equation (8) holds between the minimum norm ℓ_2 balancing weights and minimum norm OLS estimators. Because the minimum norm ℓ_2 balancing weights do not achieve exact balance, we change the notation from $\hat{w}_{\text{exact}}$ to $\hat{w}_{\ell_2}^0$. In this setting, $\|\cdot\|_* = \|\cdot\|_2$ and the minimum-norm balancing weights problem in Equation (11) is also a minimum norm linear regression, but of $\bar{\Phi}_q \in \mathbb{R}^d$ on $\Phi_p^\top \in \mathbb{R}^{d \times n}$:

$$\hat{w}_{\ell_2}^0 = \Phi_p^\top (\Phi_p^\top \Phi_p)^\dagger \bar{\Phi}_q = (\Phi_p \Phi_p^\top)^{-1} \Phi_p \bar{\Phi}_q.$$

[5] For example, when $\Phi_p \in \mathcal{H}^n$ for an infinite-dimensional Hilbert space $\mathcal{H}$, $(\Phi_p^\top \Phi_p)^\dagger$ is guaranteed to exist, since the operator $\Phi_p^\top \Phi_p$ is bounded and has closed range.

Therefore, Equation (8) holds by replacing the inverse with the pseudo-inverse:

$$
\begin{aligned}
\hat{\mathbb{E}}[\Phi_q \hat{\beta}_{\text{ols}}] &= \hat{\mathbb{E}}[\hat{w}^0_{\ell_2} \circ Y_p] \\
\hat{\mathbb{E}}[\Phi_q \underbrace{(\Phi_p^\top \Phi_p)^\dagger \Phi_p^\top Y_p}_{\hat{\beta}_{\text{ols}}}] &= \hat{\mathbb{E}}[\underbrace{\bar{\Phi}_q (\Phi_p^\top \Phi_p)^\dagger \Phi_p^\top}_{\hat{w}^0_{\ell_2}} \circ Y_p], \\
\hat{\mathbb{E}}[\Phi_q \underbrace{\Phi_p^\top (\Phi_p \Phi_p^\top)^{-1} Y_p}_{\hat{\beta}_{\text{ols}}}] &= \hat{\mathbb{E}}[\underbrace{\bar{\Phi}_q \Phi_p^\top (\Phi_p \Phi_p^\top)^{-1}}_{\hat{w}^0_{\ell_2}} \circ Y_p].
\end{aligned}
$$

B.3 Propositions 3.1 and 3.2 when $d > n$

The results in Propositions 3.1 and 3.2 apply to the setting where $d > n$ without any further alteration using the pseudo-inverse.

Proof of Proposition 3.1.

$$
Y_p^\top \Phi_p \hat{\theta}^\delta = Y_p^\top \Phi_p \Phi_p^\dagger \Phi_p \hat{\theta}^\delta = Y_p^\top \Phi_p (\Phi_p^\top \Phi_p)^\dagger \Phi_p^\top \Phi_p \hat{\theta}^\delta = \hat{\beta}_{\text{ols}} \hat{\Phi}_q,
$$

where the first two equalities follow from the pseudoinverse identities $A = AA^\dagger A$ and $A^\dagger = (A^\top A)^\dagger A^\top$ for any matrix A. □

Likewise Proposition 3.2 holds exactly for $\hat{\beta}_{\text{ols}}$ defined with the pseudoinverse.

B.4 The RKHS Setting

The results for $d = \infty$ can be computed efficiently for reproducing kernel Hilbert spaces. For notational simplicity, we will consider the distribution shift setting from Example 3. Let $\mathcal{H}$ be a possibly-infinite-dimensional RKHS on $\mathcal{X}$ with kernel $\mathcal{K}$ and induced feature map via the representer theorem, $\phi : \mathcal{X} \to \mathcal{H}$ with $\phi(x) = \mathcal{K}(x, \cdot)$. Let $\|\cdot\|_{\mathcal{H}}$ denote the norm of $\mathcal{H}$. Let K_p be the matrix with entries $\mathcal{K}(x_i, x_j)$, where $x_i, x_j \in \mathcal{X}$ are the ith and jth entries of X_p. Then $\Phi_p \Phi_p^\top = K_p$ is invertible.

We will write out the versions of the main results for $\mathcal{F} = \mathcal{H}$ to demonstrate how to compute the corresponding results for RKHSs even though $d = \infty$. Denote the solution to the regularized least squares problem in $\mathcal{H}$ with $\lambda \geq 0$:

$$
\hat{f}^\delta := \operatorname{argmin}_{f \in \mathcal{H}} \|f(X_p) - Y_p\|_2^2 + \lambda \|f\|_{\mathcal{H}}^2.
$$

This is equivalent to the following problem by the representer theorem:

$$
\begin{aligned}
\hat{\beta}^\delta_{\mathcal{H}} &:= \operatorname{argmin}_{\beta \in \mathbb{R}^n} \|K\beta - Y_p\|_2^2 + \lambda \beta K \beta \\
&= (K_p + \lambda I)^{-1} Y_p.
\end{aligned}
$$

Let $K_{x,p} \in \mathbb{R}^n$ be the row vector with entries $\mathcal{K}(x, x_i)$ where x is an arbitrary element of $\mathcal{X}$ and x_i is the ith entry of X_p. Then for any element $x \in \mathcal{X}$, $\hat{f}^\delta(x) = K_{x,p} \hat{\beta}^\delta_{\mathcal{H}}$. In particular, let define $K_{q,p}$ as the matrix with (i,j)th entry $\mathcal{K}(x_{qi}, x_{pj})$ where x_{qi} is the ith sample from the target population and x_{qj} is the jth entry of X_p. Furthermore, define $\bar{K}_{q,p} := \hat{\mathbb{E}}[K_{p,q}] \in \mathbb{R}^n$. Then, for any solution $\hat{w}^\delta_{\mathcal{H}}$ to the penalized form of balancing weights with function class $\mathcal{F}$ and hyperparameter $\delta \geq 0$:

$$
\hat{w}^\delta_{\mathcal{H}} = (K_p + \lambda I)^{-1} \bar{K}_{q,p}. \tag{12}
$$

The proof follows from the closed-form of Imbalance$_{\mathcal{H}}(w)$, known as the Maximum Mean Discrepancy (MMD) (Gretton et al., 2012); see, e.g., Hirshberg et al. (2019); Kallus (2020); Bruns-Smith and Feller (2022).

With these preliminaries, we immediately have the following equivalence from Hirshberg et al. (2019), which generalizes Appendix A.5 to the RKHS case:

$$\begin{aligned} \hat{\mathbb{E}}[K_{q,p}\hat{\beta}^{\delta}_{\mathcal{H}}] &= \hat{\mathbb{E}}[\hat{w}^{\delta}_{\mathcal{H}} \circ Y_p] \\ \hat{\mathbb{E}}[K_{q,p}\underbrace{(K_p + \delta I)Y_p}_{\hat{\beta}^{\delta}_{\mathcal{H}}}] &= \hat{\mathbb{E}}[\underbrace{\bar{K}_{q,p}(K_p + \delta I)^{-1}}_{\hat{w}^{\delta}_{\mathcal{H}}} \circ Y_p]. \end{aligned} \tag{13}$$

Likewise, we have the following form for Proposition 3.1. Define $\hat{K}_{q,p} := \hat{w}^{\delta T}_{\mathcal{H}} K_p$. Then, for any $\delta \geq 0$:

$$\hat{\mathbb{E}}[\hat{w}^{\delta}_{\mathcal{H}} \circ Y_p] = \hat{\mathbb{E}}[\hat{K}_{q,p}\hat{\beta}^{0}_{\mathcal{H}}].$$

The resulting expression for Proposition 3.2 is:

$$\hat{\mathbb{E}}[\hat{w}^{\delta}_{\mathcal{H}} \circ Y_p] + \hat{\mathbb{E}}\left[\left(K_{q,p} - \hat{K}^{\delta}_{q,p}\right)\hat{\beta}^{\lambda}_{\mathcal{H}}\right] = \hat{\mathbb{E}}[K_{q,p}\hat{\beta}_{\text{aug}}],$$

where the jth element of $\hat{\beta}_{\text{aug}}$ is:

$$\begin{aligned} \hat{\beta}_{\text{aug},j} &:= \left(1 - a^{\delta}_j\right)\hat{\beta}^{\lambda}_{\mathcal{H},j} + a^{\delta}_j \hat{\beta}^{0}_{\mathcal{H},j} \\ a^{\delta}_j &:= \frac{\widehat{\Delta}^{\delta}_j}{\Delta_j}, \end{aligned}$$

where $\Delta_j = \bar{K}_{q,p,j} - \bar{K}_{p,j}$ and $\widehat{\Delta}^{\delta}_j = \hat{K}^{\delta}_{q,p,j} - \bar{K}_{p,j}$ with $\bar{K}_{p,j} := \hat{\mathbb{E}}[K_p]$.

Identical versions for the RKHS setting apply to Section 4. These follow directly from the expressions above so we will omit repeating them explicitly. Importantly, equivalent versions for ℓ_∞ balancing in Section 5 do *not* follow immediately because an infinite dimensional vector space equipped with the ℓ_1 norm does not form a Hilbert space. We conjecture that such extensions could be constructed using the Reproducing Kernel Banach Space literature (Lin et al., 2022).

C Sample Splitting and Cross-Fitting

We briefly discuss the application of our numerical results in the setting with sample splitting. In standard balancing weights, we fit the weighting model θ^δ using data Φ_p from population p — and then also apply the weighting model at Φ_p. Cross-fitting breaks possible dependencies by only applying parameters on samples that were not used for estimation; this is a core technique in AutoDML (Chernozhukov et al., 2022b) and has been widely studied in the recent literature on doubly robust estimation (see, for example Newey and Robins, 2018; Kennedy, 2022). With cross-fitting, our numerical results only hold approximately, though we argue that the overall behavior of the estimators is qualitatively similar.

To illustrate this, let the n samples from population p be split into S partitions or "splits" and assume for simplicity that each split has size $n' := n/S$. Denote the split s covariates $\Phi_{p,s}$ and outcomes $Y_{p,s}$. Let $\Phi_{p,-s}$ and $Y_{p,-s}$ denote covariates and outcomes that are not in split s. As a simple example, consider cross-fit, unaugmented ℓ_2 balancing weights with parameter $\delta = 0$ (i.e., OLS). For each split, we first solve the balancing problem *out-of-sample* by solving for the coefficients as in the example in Equation (8):

$$\begin{aligned} \hat{\theta}^{0}_{-s} &:= \text{argmin}_{\theta \in \mathbb{R}^d}\left\{\|\tfrac{1}{n-n'}\theta\Phi^{\top}_{p,-s}\Phi_{p,-s} - \bar{\Phi}_q\|^2_2\right\} \\ &= (n - n')\overline{\Phi}_q(\Phi^{\top}_{p,-s}\Phi_{p,-s})^{\dagger}. \end{aligned}$$

Note that we have re-written the balancing problem to be in terms of the coefficients θ instead of the weights $w = \theta\Phi_{p,-s}$, in order to emphasize that the goal is to apply this weighting model to the split s samples to

obtain weights $\hat{\theta}^0_{-s}\Phi_{p,s}^\top$. The split s balancing weights estimate is then $\frac{1}{n'}\hat{\theta}^0_{-s}\Phi_{p,s}^\top Y_{p,s}$ and the final cross-fit estimator averages over these splits:

$$\frac{1}{S}\sum_{s=1}^{S}\hat{\theta}^0_{-s}\Phi_{p,s}^\top Y_{p,s}.$$

Note that the coefficients $\hat{\theta}^0_{-s}$ enforce exact balance — but only for data outside split s. In general, these weights will not achieve exact balance for split s. That is:

$$\|\tfrac{1}{n-n'}\hat{\theta}^0_{-s}\Phi_{p,-s}^\top\Phi_{p,-s} - \bar{\Phi}_q\|_2 = 0,$$
$$\|\tfrac{1}{n'}\hat{\theta}^0_{-s}\Phi_{p,-s}^\top\Phi_{p,s} - \bar{\Phi}_q\|_2 \neq 0.$$

For an augmented estimator, we would also fit an outcome model using data from outside split s. For example, we could fit OLS:

$$\hat{\beta}_{\text{ols},-s} := (\Phi_{p,-s}^\top\Phi_{p,-s})^\dagger\Phi_{p,-s}^\top Y_{p,-s}.$$

The augmented estimator combining $\hat{\theta}^0_{-s}$ with $\hat{\beta}_{\text{ols},-s}$ would give:

$$\frac{1}{S}\sum_{s=1}^{S}\Big(\bar{\Phi}_q\hat{\beta}_{\text{ols},-s} + \tfrac{1}{n'}\hat{\theta}^0_{-s}\Phi_{p,s}^\top(Y_{p,s} - \Phi_{p,s}\hat{\beta}_{\text{ols},-s})\Big).$$

C.1 Proposition 3.2 with Sample Splitting

For Proposition 3.2 with sample splitting, within a single split the numerical result is identical, but the substantive point of interest is that we always shift coefficients toward the *in-sample* OLS coefficients.

Let the coefficients $\hat{\beta}^\lambda_{-s}$ and $\hat{\theta}^\delta_{-s}$ be fixed vectors; in practice they will be models fit using samples not in s. Define $\hat{\Phi}^\delta_{q,s} := \frac{1}{n'}\hat{\theta}^\delta_{-s}\Phi_{p,s}^\top\Phi_{p,s}$, and $\hat{\beta}_{\text{ols},s} := (\Phi_{p,s}^\top\Phi_{p,s})^\dagger\Phi_{p,s}^\top Y_{p,s}$. Then the augmented estimator in the sth partition follows immediately from Proposition 3.2 in the main text:

$$\hat{\mathbb{E}}[\Phi_q\hat{\beta}_{\text{aug},s}],$$

where the jth element of $\hat{\beta}_{\text{aug},s}$ is:

$$\hat{\beta}_{\text{aug},s,j} := \big(1 - a^\delta_{j,s}\big)\,\hat{\beta}^\lambda_{-s,j} + a^\delta_{j,s}\hat{\beta}_{\text{ols},s,j}$$

$$a^\delta_{j,s} := \frac{\widehat{\Delta}^\delta_{j,s}}{\Delta_{j,s}},$$

where $\Delta_{j,s} = \bar{\Phi}_{q,j} - \bar{\Phi}_{p,s,j}$ and $\widehat{\Delta}^\delta_{j,s} = \hat{\Phi}^\delta_{q,s,j} - \bar{\Phi}_{p,j}$.

C.2 Unregularized Outcome Model

Whereas Proposition 3.2 is unchanged by sample splitting, some of the equivalence results are affected by sample splitting. For example, we know from Robins et al. (2007) that when the base learner is unregularized, i.e., $\hat{\beta}^\lambda_{\text{reg}} = \hat{\beta}_{\text{ols}}$, then the entire estimator collapses to OLS alone. With sample splitting, this is only true if $\hat{\beta}^\lambda_{\text{reg}} = \hat{\beta}_{\text{ols},s}$. With cross-fitting, however, the outcome model would typically be estimated using only data from outside split s.

For example, consider $\hat{\beta}_{\text{ols},-s}$ introduced above. Plugging this into the result in Appendix C.1 yields:

$$\hat{\beta}_{\text{aug},s,j} := \big(1 - a^\delta_{j,s}\big)\,\hat{\beta}_{\text{ols},-s,j} + a^\delta_{j,s}\hat{\beta}_{\text{ols},s,j}.$$

When the OLS coefficients are fit out of sample, this prevents overfitting to the ℓth split. Augmentation shifts the out-of-split OLS coefficents back toward the in-split OLS coefficients. As the sample size in each split goes to infinity, then both $\hat{\beta}_{\text{ols},s}$ and $\hat{\beta}_{\text{ols},-s}$ converge to the same population OLS coefficients and the augmented coefficients converge to standard OLS for any weighting model $\theta^\delta \in \mathbb{R}^d$.

C.3 Unregularized Weight Model

Consider the opposite case where $\hat{\beta}^{\lambda}_{\text{reg}}$ is arbitrary and the weight model θ^0_{-s} achieves exact balance between $\Phi_{p,-s}$ and Φ_q, as defined above. Then, as suggested in Appendix C.1, $\hat{\Phi}^0_{q,s} := \hat{\theta}^0_{-s}\Phi^\top_{p,s}\Phi_{p,s} \neq \bar{\Phi}_q$ in general. Instead, we only have an approximation:

$$a^{\delta}_{j,s} := \frac{\hat{\Phi}^0_{q,s,j} - \bar{\Phi}_{p,s,j}}{\bar{\Phi}_{q,j} - \bar{\Phi}_{p,s,j}} \approx 1,$$

where the approximation becomes equality as the sample size in each split goes to infinity. As a result, $\hat{\beta}_{\text{aug},s} \approx \hat{\beta}_{\text{ols},s}$, the in-split OLS coefficients, where again these coefficients are equal as the sample size in each split goes to infinity.

C.4 "Double Ridge"

Similarly, whereas Proposition 4.3 reduced to a single ridge outcome model, with sampling splitting we instead obtain an affine combination of in-split and out-of-split ridge regressions. Let $\hat{\beta}^{\lambda}_{-s}$ and $\hat{\theta}^{\delta}_{-s}$ denote ridge and ℓ_2 balancing coefficients respectively fit outside of the ℓth split. For notational simplicity, assume that $(\Phi^\top_{p,s}\Phi_{p,s}) = \text{diag}(\sigma^2_{1,s}, ..., \sigma^2_{d,s})$ and similarly for $-s$. Then the augmented estimator in the sth split equals $\hat{\mathbb{E}}[\Phi_q\hat{\beta}_{\text{aug},s}]$, where

$$\hat{\beta}_{\text{aug},s,j} := \left(\frac{\sigma^2_{j,-s} - \sigma^2_{j,s} + \delta}{\sigma^2_{j,-s} + \delta}\right)\hat{\beta}^{\lambda}_{-s,j} + \left(\frac{\sigma^2_{j,s} + \delta}{\sigma^2_{j,-s} + \delta}\right)\hat{\beta}^{\delta}_{s,j}.$$

D Nonlinear weights

Our main results focus on *linear* balancing weights, where $\hat{w} = \hat{\theta}\Phi^\top_p$. A rich tradition in survey statistics (e.g., Deville and Särndal, 1992), machine learning (e.g., Menon and Ong, 2016), and causal inference (e.g., Zhao, 2019) focuses instead on *non-linear* balancing weights, such as when the weights correspond to a specific *link function* $g(\cdot)$ applied to the linear predictor, $\hat{w} = g(\hat{\theta}\Phi^\top_p)$, or, equivalently, when the balancing weights problem penalizes an alternative dispersion penalty. Other balancing weights estimators are inherently nonlinear, for instance with weights estimated via a neural network (Chernozhukov et al., 2022a).

In this section, we extend our results to nonlinear weights. We first consider general, arbitrary weights. We then consider the special case of minimum-variance weights that are constrained to be non-negative, which includes many important examples (Zubizarreta, 2015; Athey et al., 2018; Abadie et al., 2010). For this special case, we show that the non-negativity constraint corresponds to sample trimming.

Before turning to these results, we briefly review several important examples of nonlinear weights.

- **Entropy balancing and alternative dispersion measures.** There is a large literature exploring alternative dispersion penalties for balancing weights and other calibrated estimators; see Deville and Särndal (1992) for a discussion in survey sampling and Zhao (2019) for a discussion in causal inference. The most common alternative to the variance penalty is *entropy balancing* (Hainmueller, 2012), which has a dual representation as linear weights passed through an exponential link function, $\hat{w} = \exp(\hat{\theta}\Phi^\top_p)$. For estimands like the Average Treatment Effect on the Treated, this implies a (calibrated) logistic regression model for the propensity score:

$$\hat{w} = \exp(\hat{\theta}\Phi^\top_p) = \text{logit}^{-1}(\hat{\theta}\Phi^\top_p) / \left(1 - \text{logit}^{-1}(\hat{\theta}\Phi^\top_p)\right).$$

  See Tan (2020) for further discussion.

- **Traditional IPW via maximum likelihood estimation.** Importantly, the form above is purely numeric and does not depend on the particular estimation procedure for the dual parameters, θ. Thus, our results below also apply to the common practice of IPW with a maximum likelihood (or other) estimate of a logistic regression propensity score model, as well as to AIPW and *Double Machine Learning* estimators (Chernozhukov et al., 2018).
- **Generalized Empirical Likelihood.** The balancing weights literature is closely related to the empirical likelihood approach, which also finds weights over units to estimate an outcome model. Examples of (generalized) empirical likelihood in similar settings include Hellerstein and Imbens (1999), Qin and Zhang (2007), Newey and Smith (2004), Graham et al. (2012), and Duchi et al. (2021). See Hirano et al. (2003, Sec 3) for a didactic example connecting inverse propensity score weighting, outcome modeling, and the empirical likelihood approach. Standard Empirical Likelihood estimators penalize the log of the weights, $\log(w)$. Many other dispersion choices are common; see, for example, Newey and Smith (2004).

Our results also apply to weighting methods that do not have this same structure, such as Covariate Balancing Propensity Scores (Imai and Ratkovic, 2014). Finally, the implied weights could also arise from an outcome model that can be represented as a linear smoother, such as a random forest; see, for example, Lin et al. (2022).

D.1 General nonlinear weights

We now show that Proposition 3.1 holds approximately for arbitrary, non-linear weights.

Proposition D.1. *Let $\hat{w} \in \mathbb{R}^n$, be any arbitrary weights. Denote the corresponding weighted features $\hat{\Phi}_q := \frac{1}{n}\hat{w}\Phi_p$. Let $\hat{\beta}_{ols} = (\Phi_p^\top \Phi_p)^\dagger \Phi_p^\top Y_p$ be the OLS coefficients of the regression of Y_p on Φ_p. Then for any $\eta \in \mathbb{R}^d$, $c \in \mathbb{R}$:*

$$\hat{\mathbb{E}}\left[\hat{w} \circ Y_p\right] = \hat{\Phi}_q \hat{\beta}_{ols} + \underbrace{(\hat{w} - \Phi_p \eta - c)^\top (Y_p - \Phi_p \hat{\beta}_{ols})}_{\text{approximation error}}.$$

Importantly, Proposition D.1 holds for arbitrary weights and, as we discuss above, does not require estimating the weights via a balancing weights optimization problem.

Because Proposition D.1 holds for any η and c, we have the following bound on the approximation error:

$$\left|\hat{\mathbb{E}}\left[\hat{w} \circ Y_p\right] - \hat{\Phi}_q \hat{\beta}_{\text{ols}}\right| \leq \min_{c,\eta} \left\|\hat{w} - \Phi_p \eta - c\right\|_2 \left\|Y_p - \Phi_p \hat{\beta}_{\text{ols}}\right\|_2, \tag{14}$$

where we could replace the 2-norms with any valid Hölder inequality pair.

The approximation error will necessarily depend on the specific data and target functional. We can nonetheless offer some remarks. First, when $d > n$, we have $\|Y_p - \Phi_p \hat{\beta}_{\text{ols}}\|_2 = 0$, and we recover Proposition 3.1 exactly. Thus, in the high dimensional setting, Proposition 3.2 holds exactly for arbitrary non-linear weights. For $n > d$, our bound on the approximation error will generally be non-zero and depends on the size of the deviations of $\hat{w}$ from its linear projection — a natural measure of the "non-linearity" of the weights. For a given weight vector, we can compute this measure by first fitting an unpenalized linear regression of the weights on Φ_p and then taking the norm of the residuals. By a simple Taylor approximation argument, this value will be small when Φ_p is tightly concentrated around its mean.

Finally, following the argument in Section 3, we can extend this proposition to augmented estimators with nonlinear weights: the augmented estimator equals $\hat{\mathbb{E}}[\Phi_q \hat{\beta}_{\text{aug}}]$ (just as before) plus the approximation error in Proposition D.1. Therefore if *nonlinear* weights $\hat{w}$ give exact balance, i.e., if $\hat{\Phi}_q = \bar{\Phi}_q$, then $\hat{\beta}_{\text{aug}} = \hat{\beta}_{\text{ols}}$, and the final point estimate differs from OLS by the approximation error in Proposition D.1.

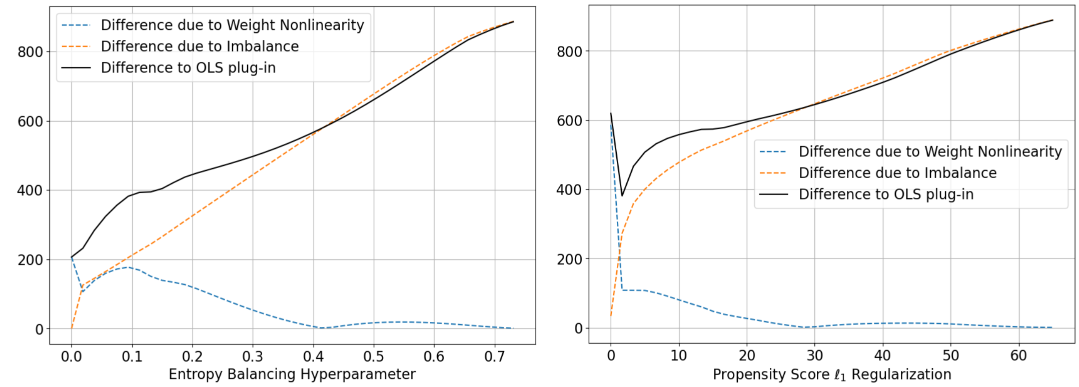

(a) Entropy Balancing (b) Traditional IPW

Figure D.1: Decomposing the estimate from nonlinear augmented balancing weights for the "short" LaLonde (1986) example.

D.1.1 Illustration: LaLonde

As an illustration, we compute both entropy balancing weights and logistic regression IPW weights for the "short" LaLonde dataset with $d = 11$ features. When targeting the Average Treatment Effect on the Treated, both weights have the same exponential link form.

We use a (cross-validated) lasso-penalized regression for the outcome model, and compute the corresponding augmented estimators, $\hat{\psi} := \bar{\Phi}_q^\top \hat{\beta}_{\text{reg}} + \hat{w}^\top (Y_p - \Phi_p \hat{\beta}_{\text{reg}})$, and augmented coefficients $\hat{\beta}_{\text{aug}}$ as defined in Proposition 3.2. We compare the augmented estimators to the plug-in OLS estimate via a triangle-inequality-type decomposition:

$$\begin{aligned}
\text{Difference to OLS plug-in: } & |\bar{\Phi}_q^\top \hat{\beta}_{\text{ols}} - \hat{\psi}| \\
\text{Difference due to weight non-linearity: } & |\bar{\Phi}_q^\top \hat{\beta}_{\text{aug}} - \hat{\psi}| \\
\text{Difference due to imbalance: } & |\bar{\Phi}_q^\top \hat{\beta}_{\text{ols}} - \bar{\Phi}_q^\top \hat{\beta}_{\text{aug}}|
\end{aligned}$$

We plot these metrics as a function on the entropy balancing hyperparameter and an ℓ_1 penalty for the propensity score model in Figure D.1.

Figure D.1 shows that there are two countervailing features at play here. First, as the weights become linear, $\hat{\psi} \to \bar{\Phi}_q \hat{\beta}_{\text{aug}}$ with the difference quantified by Proposition D.1. This occurs as the regularization strength increases because regularization pushes the weights to be uniform. Second, as the imbalance goes to zero, $\hat{\beta}_{\text{aug}} \to \hat{\beta}_{\text{ols}}$. For entropy balancing, this happens exactly as the hyperparamater goes to zero. The IPW weights typically yield substantially larger imbalance, and this imbalance does not vanish as the regularization parameter goes to zero.

D.2 Constraining weights to be non-negative

A common modification of the (minimum variance) balancing weights problem is to constrain the estimated weights to be non-negative.[6] Such weights have a number of attractive practical properties: they limit extrapolation; they ensure that the final weighting estimator is sample bounded; and they are typically sparse, which can sometimes aid interpretability (Robins et al., 2007; Abadie et al., 2010). Examples of constrained ℓ_∞ weights include Stable Balancing Weights and extensions (Zubizarreta, 2015; Athey et al.,

[6]Recall that we already impose the constraint that the weights sum to 1. So imposing non-negativity is equivalent to constraining the weights to be on the simplex.

2018; Wang and Zubizarreta, 2020); examples of constrained ℓ_2 weights include popular variants of the synthetic control method (Abadie et al., 2010; Ben-Michael et al., 2021b).

Using the dual form of the problem, Ben-Michael et al. (2021a) show that (minimum variance) linear balancing weights with a non-negativity constraint have the form $\hat{w} = \{\Phi_p \hat{\theta}^\delta\}_+$, where $\{x\}_+ = \max(x, 0)$ and where the coefficients $\hat{\theta}^\delta$ are generally different from the corresponding coefficients in the unconstrained model.[7] We can apply this insight to extend Proposition 3.1 to non-negative weights.

Proposition D.2. *Let $\hat{w}_+^\delta := \{\Phi_p \hat{\theta}^\delta\}_+$, with $\hat{\theta}^\delta \in \mathbb{R}^d$ and where $\{x\}_+ = max(x, 0)$, be any linear balancing weights with a non-negativity constraint, with corresponding weighted covariates $\hat{\Phi}_q^\delta := \hat{w}_+ \Phi_p$. Let Φ_{p+}, and Y_{p+} denote the respective quantities restricted to those data points where $\hat{w}_+^\delta > 0$. Let $\hat{\beta}_{ols}^+ := (\Phi_{p+}^\top \Phi_{p+})^{-1} \Phi_{p+}^\top Y_{p+}$ be the OLS coefficients of the regression of Y_{p+} on Φ_{p+}. Then:*

$$\hat{\mathbb{E}}\left[\hat{w}_+^\delta \circ Y_p\right] = \hat{\Phi}_q^\delta \hat{\beta}_{ols}^+.$$

So with the non-negativity constraint, we recover Proposition 3.1 almost exactly, except that the usual OLS coefficients from the entire population p, $\hat{\beta}_{\text{ols}}$, are replaced with the OLS coefficients from only those units with positive weight, $\hat{w}_+^\delta > 0$, $\hat{\beta}_{\text{ols}}^+$. We can therefore view the non-negativity constraint as a form of sample trimming. In particular, we can think of the data points where $\hat{w}_+^\delta = 0$ as a set of outliers — too dissimilar from the target population Φ_q — that we trim before applying OLS. But the key is that the definition of "outlier" depends the choice of δ and $\mathcal{F}$, and even the target covariates Φ_q: in general, changing δ, $\mathcal{F}$, or Φ_q will change the set where $\hat{w}_+^\delta > 0$. Defining and characterizing how this set changes is a promising avenue for future research. Finally, note that it is not generally the case that re-estimating the balancing weights problem after sample trimming will lead to non-negative weights (though this does hold for OLS).

Proposition D.2 simplifies further when the balancing weights achieve exact balance; see Rubinstein et al. (2021, Proposition 10) for a discussion of this special case. When $\hat{\Phi}_q = \Phi_q$, the balancing weight estimator with the non-negativity constraint is equivalent to $\hat{\mathbb{E}}[\Phi_q \hat{\beta}_{\text{ols}}^+]$. Thus, linear balancing weights with a simplex constraint is equivalent to trimming the control group and applying standard OLS (note that the trimming does not affect the target covariate profile, Φ_q).

Finally, we can extend the results in Proposition 3.2 for augmented balancing weights to incorporate a non-negativity constraint. Many popular augmented balancing weights estimators have the form of Proposition 3.2, including Athey et al. (2018) and Ben-Michael et al. (2021b). Understanding the implications of this connection is an interesting direction for future work.

D.3 Non-Linear Differentiable Outcome Models

In this section, we provide a very preliminary sketch of how the results might be extended to the case where $\mathcal{F}$ is non-linear but still differentiable in its parameters.

Let $\mathcal{F} = \{f(X, \theta) : \theta \in \mathbb{R}^d, \nabla_\theta f(X, \theta) \text{ exists}\}$. Then just like Proposition 3.1 relates any w that are linear in X to the OLS coefficients, we can relate any $w \in \mathcal{F}$ to the least squares regressor in the function class $\mathcal{F}$.

First, let θ_{LS} be the unregularized least squares regressor (where we choose the least norm θ to break ties):

$$\theta_{\text{LS}} := \min_\theta \|Y_p - f(\theta, X_p)\|_2^2$$

We have the first-order condition:

$$\nabla_\theta f(\theta_{\text{LS}}, X_p)^\top (Y_p - f(\theta_{\text{LS}}, X_p)) = 0$$

[7] This "positive part link" representation is unique to the minimum variance weights. Other dispersion penalties, such as the entropy of the weights, imply a different link function. See Zhao (2019) and Ben-Michael et al. (2021a).

Now we can get a version of Proposition 2.1 for $w \in \mathcal{F}$ by considering the following taylor expansion:

$$w(X_p) \coloneqq f(\theta_w, X_p) \approx f(\theta_{\text{LS}}, X_p) + \nabla_\theta f(\theta_{\text{LS}}, X_p)(\theta_w - \theta_{\text{LS}})$$

In which case, applying the first-order condition above, we get:

$$w(X_p)^\top y \approx \underbrace{w(X_p)^\top f(\theta_{\text{LS}}, X_p)}_{\text{identical to 3.1}} + \underbrace{f(\theta_{\text{LS}}, X_p)^\top (Y_p - f(\theta_{\text{LS}}, X_p))}_{\text{this term is zero in linear case}}.$$

E Results with Correlated Features

E.1 Overview

Throughout the main text, we have made the assumption that $\Phi_p^\top \Phi_p$ is a diagonal matrix. This is a useful simplifying assumption for illustrating the intuition behind our results. Our central result, Proposition 3.2, holds for arbitrary $\Phi_p^\top \Phi_p$. However, when $\Phi_p^\top \Phi_p$ is not diagonal, i.e. the covariates are correlated, there are some important additional subtleties for interpreting the results.

In Appendix E.2, we show that the results in the main text for ℓ_2 balancing weights still hold for non-diagonal $\Phi_p^\top \Phi_p$ by applying an additional rotation step. By constrast, in Appendix E.3, we demonstrate that diagonal $\Phi_p^\top \Phi_p$ is *not* without loss of generality for ℓ_∞ balancing. Not only do the weights not have a closed form, but the resulting augmented coefficients will not be sparse.

E.2 Augmented ℓ_2 Balancing Weights with Correlated Features

To begin, we provide the following characterization of ℓ_2 augmenting with correlated features *directly*, that is, without using Proposition 3.2:

Proposition E.1. *Let $\hat{w}^\delta_{\ell_2}$ denote the solution to the penalized linear form of balancing weights with parameter δ and $\mathcal{F} = \{f(x) = \theta^\top \phi(x) : \|\theta\|_2 \leq r\}$. Then,*

$$\hat{w}^\delta_{\ell_2} = \Phi_p(\Phi_p^\top \Phi_p + \delta I)^{-1} \overline{\Phi}_q.$$

Therefore, the augmented ℓ_2 balancing weights estimator with outcome model $\hat{\beta}_{reg} \in \mathbb{R}^d$ has the form,

$$\begin{aligned} &\hat{\mathbb{E}}[\Phi_q \hat{\beta}_{reg}] + \hat{\mathbb{E}}[\hat{w}^\delta_{\ell_2}(Y_p - \Phi_p \hat{\beta}_{reg})] = \hat{\mathbb{E}}[\Phi_q \hat{\beta}_{\ell_2}], \\ &\hat{\beta}_{\ell_2} \coloneqq (I - A_\delta)\hat{\beta}_{reg} + A_\delta \hat{\beta}_{ols} \\ &A_\delta \coloneqq (\Phi_p^\top \Phi_p + \delta I)^{-1}(\Phi_p^\top \Phi_p). \end{aligned} \tag{15}$$

For comparison, ridge regression with parameter δ has closed-form coefficients:

$$\hat{\beta}^\delta_{ridge} = (\Phi_p^\top \Phi_p + \delta I)^{-1} \Phi_p^\top Y_p = A_\delta \beta_{ols}.$$

This is a generalization of Proposition 4.1 to the correlated setting. The result follows by simply plugging in the closed-form of $\hat{w}^\delta_{\ell_2}$ into the augmented estimator. We will now show that the resulting $\hat{\beta}_{\ell_2}$ is equivalent to applying Proposition 3.2 to rotated versions of the outcome and weighting coefficients.

First, we define the rotated version of Proposition 3.2:

Proposition E.2. *Let $\Phi_p^\top \Phi_p$ be arbitrary, with eigendecomposition $\Phi_p^\top \Phi_p = VD^2V^\top$ where D^2 is a diagonal matrix with jth entry σ_j^2. For any $\hat{\beta}_{reg}^\lambda \in \mathbb{R}^d$, and any linear balancing weights estimator with estimated coefficients $\hat{\theta}^\delta \in \mathbb{R}^d$, and with $\hat{w}^\delta := \hat{\theta}^\delta \Phi_p^\top$ and $\hat{\Phi}_q^\delta := \hat{w}^\delta \Phi_p$, the resulting augmented estimator*

$$\begin{aligned} &\hat{\mathbb{E}}[\hat{w}^\delta \circ Y_p] + \hat{\mathbb{E}}\left[\left(\Phi_q - \hat{\Phi}_q^\delta\right)\hat{\beta}_{reg}^\lambda\right] \\ &= \hat{\mathbb{E}}[\Phi_q V \hat{\beta}_{aug}^{rot}], \end{aligned}$$

where the jth element of $\hat{\beta}_{aug}^{rot}$ is:

$$\begin{aligned} \hat{\beta}_{aug,j}^{rot} &:= \left(1 - a_j^{rot}\right)\hat{\beta}_{reg,j}^{rot} + a_j^{rot}\hat{\beta}_{ols,j}^{rot} \\ a_j^{rot} &:= \frac{\widehat{\Delta}_j^{rot}}{\Delta_j^{rot}}, \end{aligned}$$

defined in terms of the rotated quantities:

$$\begin{aligned} \hat{\beta}_{reg}^{rot} &:= V^\top \hat{\beta}_{reg}^\lambda, \\ \hat{\beta}_{ols}^{rot} &:= V^\top \hat{\beta}_{ols}, \\ \widehat{\Delta}^{rot} &:= (\hat{\Phi}_q - \bar{\Phi}_p)V, \\ \Delta^{rot} &:= (\bar{\Phi}_q - \bar{\Phi}_p)V. \end{aligned}$$

Proof. First notice that:

$$\begin{aligned} &\hat{\mathbb{E}}[\hat{w}^\delta \circ Y_p] + \hat{\mathbb{E}}\left[\left(\Phi_q - \hat{\Phi}_q^\delta\right)\hat{\beta}_{\text{reg}}^\lambda\right] \\ &= \hat{\mathbb{E}}\left[\hat{\Phi}_q^\delta \hat{\beta}_{\text{ols}} + \left(\Phi_q - \hat{\Phi}_q^\delta\right)\hat{\beta}_{\text{reg}}^\lambda\right] \\ &= \hat{\mathbb{E}}\left[\hat{\Phi}_q^\delta VV^\top \hat{\beta}_{\text{ols}} + \left(\Phi_q - \hat{\Phi}_q^\delta\right)VV^\top\hat{\beta}_{\text{reg}}^\lambda\right] \end{aligned}$$

Then the result follows immediately from Proposition 3.2 applied to the covariates $\Phi_p V$ and $\Phi_q V$ with weights $\hat{w}$ and outcome model $V^\top \hat{\beta}_{\text{reg}}^\lambda$. □

Note that we could also compute $\hat{\beta}_{\text{aug}}$ directly as in Proposition 3.2 because the original derivation does not depend on $\Phi_p^\top \Phi_p$ being diagonal. Combining the two propositions we have that:

$$\hat{\mathbb{E}}[\Phi_q \hat{\beta}_{\text{aug}}] = \hat{\mathbb{E}}[\Phi_q V \hat{\beta}_{\text{aug}}^{\text{rot}}].$$

However, *it is not the case* that $\hat{\beta}_{\text{aug}} = V\hat{\beta}_{\text{aug}}^{\text{rot}}$ in general. For ℓ_2 balancing weights, $V\hat{\beta}_{\text{aug}}^{\text{rot}}$ is the correct generalization of Proposition 3.2 to the correlated setting as we demonstrate in the next few propositions.

Proposition E.3. *Let $\hat{\beta}_{\ell_2}$ be defined as in Equation (15). For the same weights $\hat{w}_{\ell_2}^\delta$, let $\hat{\beta}_{aug}$ and $\hat{\beta}_{aug}^{rot}$ be defined as in Propositions 3.2 and E.2 respectively. Then for any $\Phi_p^\top \Phi_p$ (diagonal or not):*

$$\hat{\mathbb{E}}[\Phi_q \hat{\beta}_{\ell_2}] = \hat{\mathbb{E}}[\Phi_q V \hat{\beta}_{aug}^{rot}] = \hat{\mathbb{E}}[\Phi_q \hat{\beta}_{aug}].$$

Additionally, when $\Phi_p^\top \Phi_p$ is diagonal, then

$$\hat{\beta}_{\ell_2} = V\hat{\beta}_{aug}^{rot} = \hat{\beta}_{aug}.$$

However, when $\Phi_p^\top \Phi_p$ is not diagonal, then in general

$$\hat{\beta}_{\ell_2} = V\hat{\beta}_{aug}^{rot} \neq \hat{\beta}_{aug}.$$

Proof. The fist claims follows from Propositions 3.2, E.2, and E.1. For the second claim, if $\Phi_p^\top \Phi_p$ is diagonal, then $V = I$ and $\hat{\beta}_{\text{aug}} = \hat{\beta}_{\text{aug}}^{\text{rot}}$. Next we will show for general $\Phi_p^\top \Phi_p$ that $\hat{\beta}_{\ell_2} = V\hat{\beta}_{\text{aug}}^{\text{rot}}$. Note that

$$A_\delta = V(D^2 + \delta I)^{-1} D^2 V^\top,$$

and that

$$\begin{aligned}\hat{\Delta}^{\text{rot}} &= \overline{\Phi}_q(\Phi_p^\top \Phi_p + \delta I)^{-1}(\Phi_p^\top \Phi_p)V \\ &= \overline{\Phi}_q V(D^2 + \delta I)^{-1} D^2\end{aligned}$$

and so $\text{diag}(a^{\text{rot}}) = (D^2 + \delta I)^{-1} D^2$. Therefore,

$$\begin{aligned}V\hat{\beta}_{\text{aug}}^{\text{rot}} &= V(I - \text{diag}(a^{\text{rot}}))V^\top \hat{\beta}_{\text{reg}} + V\text{diag}(a^{\text{rot}})V^\top \hat{\beta}_{\text{ols}} \\ &= (VV^\top - A_\delta)\hat{\beta}_{\text{reg}} + A_\delta \hat{\beta}_{\text{ols}} \\ &= (I - A_\delta)\hat{\beta}_{\text{reg}} + A_\delta \hat{\beta}_{\text{ols}}.\end{aligned}$$

Finally, for a counter-example where $V\hat{\beta}_{\text{aug}}^{\text{rot}} \neq \hat{\beta}_{\text{aug}}$, see Table E.1. □

Proposition E.4. *Consider the setting in Proposition E.3, but now assume that the outcome model $\hat{\beta}_{reg} = \hat{\beta}_{ridge}^{\lambda}$, the ridge regression coefficients of Y_p on Φ_p with hyperparameter λ. Then:*

$$\hat{\beta}_{\ell_2} = V(D^2 + \mathit{diag}(\gamma))^{-1} V^\top \Phi_p^\top Y_p,$$

where $\mathit{diag}(\gamma)$ is the diagonal matrix with jth entry

$$\gamma_j \coloneqq \frac{\delta\lambda}{\sigma_j^2 + \lambda + \delta}.$$

Furthermore,

$$\|\hat{\beta}_{ridge}^{\lambda}\|_2 \leq \|\hat{\beta}_{\ell_2}\|_2 = \|V\hat{\beta}_{aug}^{rot}\|_2 \leq \|\hat{\beta}_{ols}\|_2,$$

but in general it is possible that:

$$\|\hat{\beta}_{ols}\|_2 < \|\hat{\beta}_{aug}\|_2.$$

Proof.

$$\begin{aligned}\hat{\beta}_{\ell_2} &= (I - A_\delta)(\Phi_p^\top \Phi_P + \lambda I)^{-1} \Phi_p^\top Y_p + A_\delta(\Phi_p^\top \Phi_P)^{-1} \Phi_p^\top Y_p \\ &= V\Big[(I - (D^2 + \delta I)^{-1} D^2)(D^2 + \lambda I)^{-1} + (D^2 + \delta I)^{-1} D^2 (D^2)^{-1}\Big] V^\top \Phi_p^\top Y_p \\ &= V(D^2 + (\delta + \lambda)I)(D^2 + \delta I)^{-1}(D^2 + \lambda I)^{-1} V^\top \Phi_p^\top Y_p \\ &= V(D^2 + \text{diag}(\gamma))^{-1} V^\top \Phi_p^\top Y_p.\end{aligned}$$

We now demonstrate the two norm inequalities. First,

$$\begin{aligned}&\|\hat{\beta}_{\text{ridge}}^{\lambda}\|_2^2 \leq \|\hat{\beta}_{\ell_2}\|_2^2 \\ \iff &\|V(D^2 + \lambda I)^{-1} V^\top \Phi_p^\top Y_p\|_2^2 \leq \|V(D^2 + \text{diag}(\gamma))^{-1} V^\top \Phi_p^\top Y_p\|_2^2 \\ \iff &Y_p^\top \Phi_p(V\left[(D^2 + \lambda I)^{-1}(D^2 + \lambda I)^{-1} - (D^2 + \text{diag}(\gamma))^{-1}(D^2 + \text{diag}(\gamma))^{-1}\right] V^\top)\Phi_p^\top Y_p \geq 0 \\ \iff &(D^2 + \lambda I)^{-1}(D^2 + \lambda I)^{-1} \preceq (D^2 + \text{diag}(\gamma))^{-1}(D^2 + \text{diag}(\gamma))^{-1} \\ \iff &(D^2 + \lambda I)^{-1} \preceq (D^2 + \text{diag}(\gamma))^{-1}.\end{aligned}$$

Note that

$$\frac{1}{\sigma_j^2 + \gamma_j} = \left(\frac{1}{\sigma_j^2 + \lambda}\right)\left(\frac{\sigma_j^2 + \lambda + \delta}{\sigma_j^2 + \delta}\right), \tag{16}$$

and that

$$\frac{\sigma_j^2 + \lambda + \delta}{\sigma_j^2 + \delta} \geq 1. \tag{17}$$

Combining facts (16) and (17), we have $1/(\sigma_j^2 + \gamma_j) > 1/(\sigma_j^2 + \lambda)$, and we're done.

Similarly, for the second inequality:

$$\begin{aligned}
&\|\hat{\beta}_{\ell_2}\|_2^2 \leq \|\hat{\beta}_{\text{ols}}\|_2^2 \\
\iff &\|V(D^2 + \text{diag}(\gamma))^{-1}V^\top \Phi_p^\top Y_p\|_2^2 \leq \|V(D^2)^{-1}V^\top \Phi_p^\top Y_p\|_2^2 \\
\iff &(D^2 + \text{diag}(\gamma))^{-1} \preceq (D^2)^{-1}
\end{aligned}$$

which follows because $\gamma \geq 0$.

Finally, for a counter-example where $\|\hat{\beta}_{\text{ols}}\|_2 < \|\hat{\beta}_{\text{aug}}\|_2$, see Table E.1. □

Even when $\Phi_p^\top \Phi_p$ is diagonal, if $\hat{\beta}_{\text{reg}}$ can be arbitrary — and in particular, adversarially chosen — then we are not guaranteed to see undersmoothing. Consider an arbitrary $\hat{\beta}_{\text{reg}}$ that ℓ_2-smoothed with respect to OLS. That is, $\|\hat{\beta}_{\text{reg}}\|_2 \leq \|\hat{\beta}_{\text{ols}}\|_2$. If there are no other constraints on $\hat{\beta}_{\text{reg}}$, then there exist problem instances where $\hat{\beta}_{\ell_2}$ has ℓ_2-norm larger than $\hat{\beta}_{\text{ols}}$ or smaller than $\hat{\beta}_{\text{reg}}$. For a simple example, consider $a^\delta = [0, 1/(1+\delta)]$, and $\hat{\beta}_{\text{ols}} = [0, 1]$ — note that these are jointly realizable by some Φ_p, Y_p. Then $\hat{\beta}_{\text{reg}} = [1, 0]$ will have ℓ_2-norm bigger than 1 for sufficiently large δ.

Empirical Results for augmented ℓ_2-balancing weights. To see in practice that the rotated procedure obtains the properly undersmoothed augmented coefficients, we report norm values for ℓ_2 augmented estimators using both a ridge and a lasso base learner in the high-dimensional LaLonde and IHDP settings. The key takeaway is that $\hat{\beta}_{\ell_2} = V\hat{\beta}_{\text{aug}}^{\text{rot}}$ has the correct undersmoothing properties.

$\hat{\beta}_{\text{reg}}$	$\|\hat{\beta}_{\text{reg}}\|_2$	$\|\hat{\beta}_{\ell_2}\|_2$	$\|\hat{\beta}_{\text{ols}}\|_2$	$\|\hat{\beta}_{\text{aug}}\|_2$
LaLonde Ridge	6.495e3	1.160e4	4.469e7	4.633e7
LaLonde Lasso	7.273e3	1.154e4	4.469e7	4.633e7
IHDP Ridge	1.038	8.569	9.299	9.722
IHDP Lasso	1.262	8.666	9.299	9.626

Table E.1: The ℓ_2 norm of base learner coefficients, unrotated augmented coefficients, OLS coefficients, and properly rotated augmented coefficients. Results are reported for the LaLonde and IHDP datasets using their respective high-dimensional feature sets and with both ridge and lasso base learners.

E.3 Augmented ℓ_∞ balancing weights

When we use ℓ_∞ balancing for the weighting model (or equivalently the minimum distance lasso estimator for the Riesz representer), the story becomes more complicated. When $\Phi_p^\top \Phi_p$ is not diagonal, both the Lasso outcome model and the ℓ_∞ balancing weights lack a closed-form solution.

For Lasso with correlated features, the regularization path is qualitatively similar to the diagonal case. The coefficients start at OLS and shrink exactly to zero at different rates. However, due to the correlation between the features, each coefficient no longer linearly (or even monotonically) moves toward zero. Likewise, for ℓ_∞ balancing weights, we see similar behavior for the coefficients of the Riesz representer model. These coefficients are sparse, with regularization paths that move toward zero (but not monotonically).

In the diagonal setting, the regularization path for the augmented estimator, a_j^δ, is proportionate (element-wise) to $\hat{\theta}$. Therefore, because $\hat{\theta}$ is sparse, the augmented estimator coefficients defined in Proposition 3.2 is also sparse. For general design, the impact of $\hat{\theta}$ on the augmented coefficients also depends on the empirical covariance matrix.

Lemma E.5. *Let $\hat{w}$ be the solution to balancing weights with hyperparameter δ for $\mathcal{F} = \{f(x) = \theta^\top \phi(x) : \|\theta\| \le r\}$, where $\|\cdot\|$ is any norm. Let $\hat{\theta}$ be the corresponding solution to the dual form of balancing weights, so that $\hat{w} = \Phi_p \hat{\theta}$. Then,*

$$\hat{\Phi}_q = \hat{\theta}^\top \Sigma,$$

and

$$a_j^\delta = \frac{\hat{\theta}^\top \Sigma_j}{\bar{\Phi}_{q,j}}.$$

In the case of ℓ_∞ balancing, since $\hat{\theta}$ is sparse, a_j^δ is a sparse combination of the elements of the jth column of the empirical covariance matrix Σ. But unless this combination is exactly zero (typically a measure zero event), the resulting a^δ will not inherit sparsity from $\hat{\theta}$.

F Undersmoothing with general weights as viewed by the normal equations

Our key numerical result, Proposition 3.2, allows us to write augmented weighting estimators as a plug-in estimator using coefficients $\hat{\beta}_{\text{aug}}$. Notably, this result holds for weights $\hat{w}$ as long as $\hat{w} = \hat{\theta}\Phi_p^\top$ for *any* vector $\hat{\theta} \in \mathbb{R}^d$. The numerical result holds even if $\hat{\theta}$ doesn't result in a reasonable estimator of the Riesz representer.

It is interesting to see under what conditions on $\hat{w}$ does $\hat{\beta}_{\text{aug}}$ still behave as an "undersmoothed" estimator. We discussed some ways to define this in Appendix E.2; for example requiring that the ℓ_2-norm of the coefficieents be between that of the base learner $\hat{\beta}$ and $\hat{\beta}_{\text{ols}}$. In this section, we develop a different notion of "undersmoothing" in terms of the size of the normal equation violations. In penalized linear regression, the smaller the penalty, the closer the LHS of the normal equation gets to zero. Therefore one way to view "undersmoothing" is to measure the size of the normal equation violations. Formally, the first order condition of (unpenalized) linear regression with coefficients β is:

$$\mathcal{N}(\hat{\beta}_{\text{ols}}) \coloneqq \Phi_p^\top (Y_p - \Phi_p \hat{\beta}_{\text{ols}}) = 0.$$

Define A to be the diagonal matrix with jth entry a_j as defined in Proposition 3.2. Then the corresponding normal equation violation (the gradient of the squared loss) for $\hat{\beta}_{\text{aug}}$ is:

$$\begin{aligned}
\mathcal{N}(\hat{\beta}_{\text{aug}}) &= \Phi_p^\top (Y_p - \Phi_p A \hat{\beta}_{\text{ols}} - \Phi_p (I - A)\hat{\beta}) \\
&= \Phi_p^\top (Y - \Phi_p \hat{\beta}) + (\Phi_p^\top \Phi_p) A (\hat{\beta} - \hat{\beta}_{\text{ols}}) \\
&= \Phi_p^\top (Y - \Phi_p \hat{\beta}_{\text{ols}}) + (\Phi_p^\top \Phi_p)(A - I)(\hat{\beta} - \hat{\beta}_{\text{ols}}) \\
&= (\Phi_p^\top \Phi_p)(I - A)(\hat{\beta}_{\text{ols}} - \hat{\beta})
\end{aligned}$$

Note that when $A = 0$, we recover the original outcome model, e.g. $\hat{\beta}_{\text{aug}} = \hat{\beta}$ and so the normal equation violations of $\hat{\beta}$ can be written:

$$\mathcal{N}(\hat{\beta}) = (\Phi_p^\top \Phi_p)(\hat{\beta}_{\text{ols}} - \hat{\beta}).$$

In other words, how close $\hat{\beta}$ is to optimizing the squared loss (in terms of the size of the gradient) is just equal to how close the coefficients are to the OLS coefficients, but mediated via the sample covariance matrix. E.g. large eigenvalues of $\Phi_p^\top \Phi_p$ will amplify the difference more, and small eigenvalues less. The only difference with the augmented coefficients $\hat{\beta}_{\text{aug}}$ is the introduction of the scaling diagonal matrix $(I - A)$. If A is uniformly close to zero, it will damp the eigenvalues of the covariance matrix and result in small normal equation violations. This is most clear when the sample covariance matrix is diagonal with entries σ_j^2. In this case, the jth element of the squared loss gradient becomes:

$$\sigma_j^2(1 - a_j)(\hat{\beta}_{\text{ols},j} - \hat{\beta}_j).$$

Whenever all a_j are between 0 and 2, then the violations of the normal equations for $\hat{\beta}_{\text{aug}}$ are guarantee to be uniformly at least as small as that of $\hat{\beta}$.

G Comparison to TMLE with Balancing Weights

As pointed out in Chernozhukov et al. (2022b), the balancing weights estimator of the Riesz representer can be applied to any doubly-robust meta algorithm. In the main text, we used the standard AIPW form, but we can also consider the TMLE form Van Der Laan and Rubin (2006); Van der Laan et al. (2011). In the linear setting, for an outcome model $\hat{\beta}$ and weights $\hat{w} := \Phi_p\hat{\theta}$, the TMLE estimate is:

$$\bar{\Phi}_q^T(\hat{\beta} + \hat{\epsilon}\hat{\theta}),$$

where

$$\begin{aligned}\hat{\epsilon} &:= \text{argmin}_\epsilon \left\{ \|Y_p - \Phi_p\hat{\beta} - \epsilon\hat{w}\|_2^2 \right\} \\ &= \frac{\hat{w}^T(Y_p - \Phi_p\hat{\beta})}{\hat{w}^T\hat{w}}.\end{aligned}$$

In this section, we show the explicit connection between the AIPW and TMLE forms which are closely related. This connection can be stated in two ways, an "AIPW-centric" form and a "TMLE-centric" form.

AIPW-centric version of stating the connection:

$$\begin{aligned}&\text{AIPW:} \quad \bar{\Phi}_q^T\hat{\beta} + \frac{1}{n} \hat{w}^T(Y_p - \Phi_p\hat{\beta}) \\ &\text{TMLE:} \quad \bar{\Phi}_q^T\hat{\beta} + \underbrace{\left(\frac{\bar{\Phi}_q^T\hat{\theta}}{\hat{\theta}^T\Phi_p^T\Phi_p\hat{\theta}}\right)}_{\text{scaled ratio of terms in Riesz loss}} \hat{w}^T(Y_p - \Phi_p\hat{\beta})\end{aligned}$$

TMLE-centric version of stating the connection:

$$\begin{aligned}
\text{TMLE:}\quad & \bar{\Phi}_q^T\hat{\beta} + \hat{\epsilon}\bar{\Phi}_q^T\hat{\theta} \\
\text{AIPW:}\quad & \bar{\Phi}_q^T\hat{\beta} + \underbrace{\hat{\epsilon}\hat{\Phi}_q^T\hat{\theta}}_{\hat{\Phi}_q \text{ instead of } \bar{\Phi}_q}
\end{aligned}$$

From the "AIPW-centric" version of the connection, we see the familiar $\hat{w}^T Y_p$ term appear in the TMLE, and can apply our main results to rewrite the TMLE estimator around $\hat{\beta}_{\text{ols}}$. However, because we now weight the average by a term that is different from $\frac{1}{n}$, the results may have different variance properties. We think this is a promising direction for future research.

H Simulation Study Details

H.1 Setup

We consider 36 different data generating processes (DGPs) for our simulation study. For each of them, we compare an oracle baseline with three feasible hyperparameter tuning schemes.

For the remainder of this section, when we write "numerically optimize", we mean using the scipy minimize solver with tolerance 1e-12. In particular, we pre-compute the SVD of the covariance matrix before solving, so that we can compute the pseudoinverse for all involved expressions in closed form without performing traditional matrix inversion during optimization.

For the oracle hyperparameters, we compute λ^* by numerically optimizing the in-distribution mean squared error for ridge regression from Section 6.2. We then fix $\lambda = \lambda^*$ and then numerically optimize the expression in Proposition 6.1 to get the MSE-optimal δ^*

The three feasible hyperparameter tuning schemes we consider, by contrast, use a particular draw of Y_p. In all cases, we choose λ by cross-validating a ridge outcome model. Then we considering choosing δ by: (1) cross-validating balance, (2) cross-validating the Riesz loss, and (3) setting δ equal to the cross-validated ridge λ. In all cases, cross-validation is performed 5-fold via numerical optimization as described above instead of using a grid.

Note that the oracle hyperparameters are only optimal in a setting where we have to pick a single λ and δ for each draw of Y_p. Cross-validation picks a new λ for each Y_p and so can theoretically outperform the oracle. This happens very rarely, but a non-zero amount of the time. Overall, the results in Table 1 suggest that this is still a very good baseline. Finally, note that we could have picked λ by cross validation for the oracle, and then solved for the optimal δ^* separately for each Y_p, fixing the CV value of λ. However, we cannot guarantee that the resulting δ will have any optimality properties, since the mean squared error expression is derived by averaging over Y_p draws for fixed values of λ and δ. But this could be an interesting follow up experiment.

In all cases, we take 3000 draws of Y_p and then compute the squared error and take the average as a monte carlo estimate of the mean squared error.

H.2 Synthetic DGPs

To compute the oracle λ^* and δ^* from Section 6.2, we need: the true coefficients, β_0, the population covariance matrix, $\mathbb{E}[\Phi_p^T \Phi_p]$, a sample covariance matrix $\hat{\Sigma}$, the conditional variance σ^2, and the target covariance mean, $\mathbb{E}[\Phi_q]$. So for each DGP, we need to specify these five objects.

We consider synthetic DGPs with three basic setups. They all use $n = 2000$ and $d = 50$. For each of the three setups, we draw a random β_0 as a the absolute value of a d-dimensional standard normal normalized to have 2-norm equal to 1. The three setups each generate a population covariance matrix in roughly the same way. In each case, we choose a maximum and minimum eigenvalue, $\eta_{\min}$ and $\eta_{\max}$ respectively. We then generate an equally space grid between $\eta_{\min}^{1/c}$ and $\eta_{\min}^{1/c}$ for some curvature constant c. We choose the eigenvalues of the covariance matrix to be the numbers in this grid raised to the cth power. We then draw a random eigenvector matrix from the special orthogonal group, U, and form the covariance matrix from the eigenvectors and eigenvalues in the standard way. Next, we draw an Φ_p with $n = 2000$ samples from a mean-zero multi-variate normal with this covariance matrix, and compute $\hat{\Sigma} = \Phi_p^T \Phi_p / n$.

The three basic setups differ in the choice of $\eta_{\min}, \eta_{\max}$, and c. For setting 1 we choose $\eta_{\min} = 1e - 4$, $\eta_{\max} = 3$ and $c = 5000$. For setting 2 we choose $\eta_{\min} = 1e - 8, \eta_{\max} = 3$, and $c = 5000$. For setting 3, we choose $\eta_{\min} = 1e - 10, \eta_{\max} = 5, c = 10$.

Then for each of these basic setups, we create 10 DGPs, by consider all combinations of a list of $\mathbb{E}[\Phi_q]$ and σ^2. We use $\sigma^2 \in \{0.1, 2\}$. For $\mathbb{E}[\Phi_q]$ we use: the vector of all 0.1, the vector of all 2, and then 3 vectors chosen randomly uniformly between -1 and 1 which are then scaled to have norm 1. Thus the total of $2 \times 5 = 10$ DGPs for each setup.

H.3 Semi-Synthetic DGPs

We then also use semi-synthetic DGPs based on Lalonde Long and IHDP Long (see Appendix I for details on these datasets). For each of these datasets, we recenter Φ_p and Y_p to have mean-zero. Then we choose β_0 to be the coefficients from cross-validated ridge regression of Y_p on Φ_p. We let σ^2 be the variance of the residuals from this regression, and we choose the population covariance matrix to be $\Phi_p^T \Phi_p / n$. Next, we *redraw* a new matrix of samples from a mean-zero normal distribution with this covariance matrix, and compute the sample covariance $\hat{\Sigma}$ from these *new* samples. For targets, we use the actual $\mathbb{E}[\Phi_q]$, but also include two perturbed versions where all even elements of $\mathbb{E}[\Phi_q]$ are either increased or decreased by a proportion of the norm of $\mathbb{E}[\Phi_q]$. For IHDP, the perturbation is 1/10 times the norm, for Lalonde, the perturbation is 1/100 times the norm.

So since for each semi-synthetic setting we have three values of $\mathbb{E}[\Phi_q]$ and one value of σ^2, that corresponds to 6 DGPs each, for a total of 36 together with the synthetic DGPs.

I Additional Numerical Experiments

I.1 Dataset Summaries

The following table summarizes the number of samples and features in the datasets used for numerical illustrations. In the main text, we presented results for "LaLonde Short" and "LaLonde Long", the LaLonde (1986) data with the original 11 features and the expanded 171 features from Farrell (2015).

In Appendix I.3, we also provide results for the Infant Health and Development Program (IHDP) dataset, a standard observational causal inference benchmark from Hill (2011). IHDP is based on data from a randomized control trial of an intensive home visiting and childcare intervention for low birth weight infants born in 1985. For all children, we have a range of baseline covariates (contained in "IHDP Short"), including both categorical covariates, like the mother's educational attainment, and continuous covariates, like the child's birth weight. Our goal is to estimate the average outcome (a standardized test score) in the absence of the intensive intervention. We additionally create an "IHDP Long" extended feature set that includes all pair-wise interaction terms between the discrete covariates and a second-order polynomial expansion of all continuous covariates.

Dataset	Features	Train Samples	Test Samples
LaLonde Short	11	727	185
LaLonde Long	171	727	185
IHDP Short	25	608	139
IHDP Long	193	608	139

Table I.2: Summary of the four datasets used for numerical illustrations including the number of features, the number of control/train observations (used as samples from p) and the number of treated/test observations (used as samples from q).

I.2 Numerical Example with cross-fitting

In Section 7.2.2, we demonstrated that for the low-dimensional NSW setting, the cross-validated weighting hyperparameter is $\delta = 0$. As a result, for any linear base learner, the augmented estimator is numerically equivalent to a simple OLS plug-in estimate. As discussed in Appendix C.3, with cross-fitting this should only remain approximately true. In this section, we numerically assess the sensitivity of this result to cross-fitting.

We repeat the experiment with the 11-dimensional NSW covariates using a lasso base learner and ℓ_∞ balancing weights. We perform 5-fold cross-fitting with random splits. In each split, we compute the augmented estimate using models fit with data from the other 4 splits as described at the beginning of Appendix C. Each model has its hyperparameter chosen separately by cross-validation. Thus for 5-fold cross-fitting, we fit 5 lasso regressions and 5 balancing weights estimators each with 4/5ths the sample size and a separate hyperparameter. In the manner of bootstrapping, we re-run this end-to-end procedure 1000 times for different random choices of the splits.

Note that there are two main ways that cross-fitting might cause the final estimate to deviate from simple OLS. The first is the variation due to only applying the models out of sample. The second is that with smaller sample sizes, cross-validation might select larger values for the hyperparameter. We find that for 80% of the 5×1000 weighting models, $\delta = 0$. In the remaining 20% some of the hyperparameters take on a very small but non-zero value. The mean δ is 0.0037 and the CDF across the 5×1000 models is given in Figure I.2a.

Figure I.2b shows a smoothed density plot for the cross-fit augmented estimates over the 1000 draws. The black dotted line marks the augmented estimate without cross-fitting (recall that this is identical to the plug-in OLS estimate for this dataset). The red dotted line marks the mean of the cross-fit estimates over the 1000 draws. *On average*, cross-fitting has virtually no effect on the augmented estimate — at least in this setting where the weighting hyperparameter is usually close to zero. However, there is substantial variation across the 1000 draws. In particular, the density is skewed and the mode of the cross-fit estimates is slightly smaller than the OLS point estimate. There is also a meaningful tail of estimates larger than the OLS point estimate, including a second larger mode.

I.3 Additional Experiments

I.3.1 Low Dimensional

We begin by providing additional examples for the low dimensional setting. We repeat the experiments in Section 7.2.2 in three additional settings: the LaLonde low-dimensional setting with ℓ_2 balancing weights in Figure 4, the IHDP low-dimensional setting with ℓ_∞ balancing weights in Figure I.4, and the IHDP low-dimensional setting with ℓ_2 balancing weights in Figure I.5.

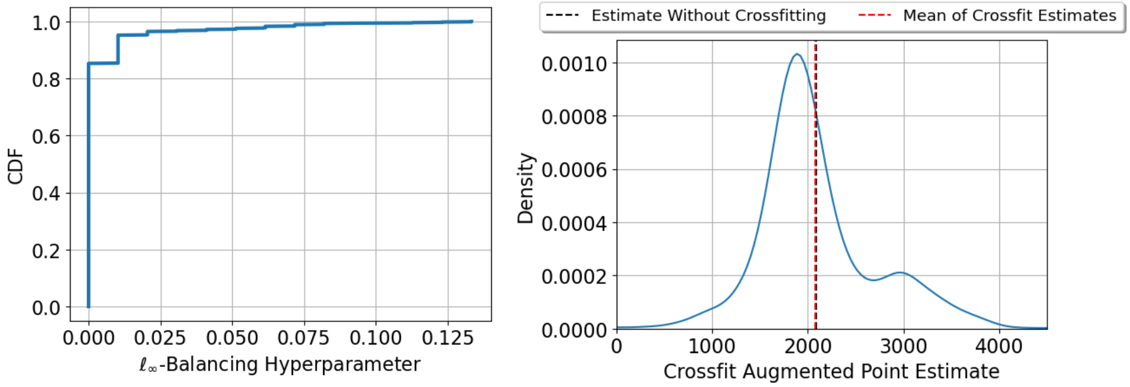

(a) Weighting Hyperparameter CDF

(b) Distribution of cross-fit Estimates

Figure I.2: Results with 5-fold cross-fitting for the LaLonde (1986) dataset with the low-dimensional set of 11 features. The cross-fitting procedure is repeated 1000 times. Panel (a) plots the empirical cumulative distribution of weighting hyperparameters over the 1000×5 weighting models; in the main text, the ℓ_∞ hyperparameter extends from 0 to 1 (here truncated at 0.12). Panel (b) shows a density plot for the cross-fit augmented point estimates over the 1000 repeats.

I.3.2 High Dimensional

We also replicate the experiments in Section 7.2.1 for high dimensional double ridge and double lasso but using the IHDP data with a high dimensional feature set. Figure I.6 replicates the reporting of point estimates and cross-validation curves for double lasso and double ridge with IHDP as in Figure 3; Figure I.7 replicates undersmoothing in double ridge as in Figure I.9; and Figure I.8 replicates undersmoothing in the ℓ_0 "norm" as in Figure I.10.

I.4 Undersmoothing in LaLonde

I.4.1 Undersmoothing for double ridge

For the special case of double ridge, we now illustrate our result from Proposition 4.3 that ℓ_2 balancing weights produce a new outcome model that is *undersmoothed* relative to the original ridge regression model. Recall that when the base learner is a generalized ridge model with parameters λ_j, then the augmented estimator is equivalent to plugging in a generalized ridge models with parameters $\gamma_j \leq \lambda_j$. In this example, the λ_j are all equal to the same value (chosen via cross-validation), indicated by the purple dotted line in Figure 3a. We compute the corresponding γ_j for δ chosen by cross-validation, indicated by the green dotted line in Figure 3b.

Figure I.9a plots the λ_j and γ_j, with j on the x-axis sorted in the order of the eigenvalues of $\Phi_p^\top \Phi_p$ from largest to smallest. Figure I.9b plots the corresponding eigenvalues. For standard ridge regression, the key idea is to push the eigenvalues of $\Phi_p^\top \Phi_p$ away from zero so that the resulting matrix $\Phi_p^\top \Phi_p + \lambda I$ is invertible and well-conditioned. Thus, the original ridge regression applies the same amount of regularization across the spectrum, as shown by the blue line in Figure I.9a. By contrast, the implied outcome model from the augmented procedure uses far less regularization and is substantially undersmoothed. Importantly, the augmented estimator undersmooths more where the eigenvalues of $\Phi_p^\top \Phi_p$ are large and undersmooths less where the eigenvalues of $\Phi_p^\top \Phi_p$ are close to zero. The augmented estimator therefore avoids bias by only regularizing the spectral directions that are the most significant sources of variation — and even these to a much smaller degree than is optimal for MSE predictions.

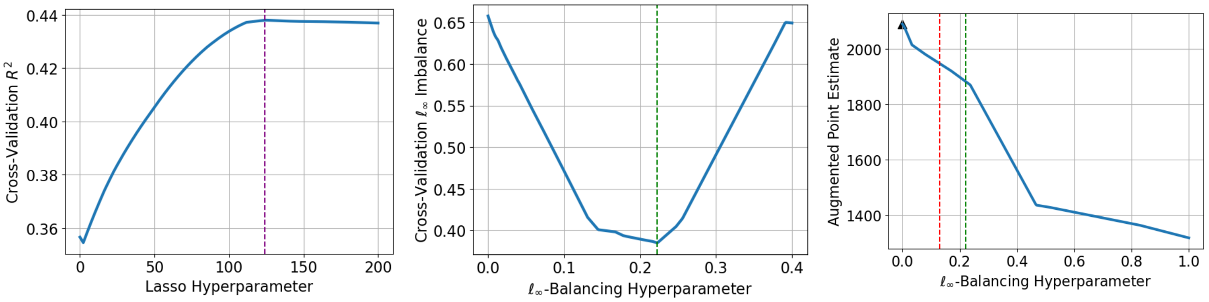

(a) Lasso outcome model

(b) ℓ_∞ balancing

(c) Augmented estimate

Figure I.3: Lasso-augmented ℓ_∞ balancing weights ("double lasso") for LaLonde (1986) with the original 11 features. Panel (a) shows the 10-fold cross-validated R^2 for the Lasso-penalized regression of Y_p on Φ_p among control units across the hyperparameter λ; the purple dotted line shows the CV-optimal value. Panel (b) shows the 10-fold cross-validated imbalance for ℓ_∞ balancing weights across the hyperparameter δ; the green dotted line shows the CV-optimal value. Panel (c) shows the point estimate for the augmented estimator across the weighting hyperparameter δ; the black triangle corresponds to the OLS point estimate, the green dotted line corresponds to cross-validating imbalance, the red dotted line corresponds to cross-validating the Riesz loss.

I.4.2 Undersmoothing in norm

For the special case with diagonal covariates, both double ridge and double lasso undersmooth in a particular norm of $\|\hat{\beta}_{\text{aug}}\|$, relative to the unaugmented outcome model. We demonstrate this phenomenon in Figure I.10, where we first use the eigenvectors of $\Phi_p^\top \Phi_p$ to decorrelate the features; as we discuss above, this is without loss of generality for ℓ_2 balancing but not for ℓ_∞ balancing. In particular, Figures I.10a and I.10c show the ℓ_2 norm and the number of included covariates (the ℓ_0 "norm") for the ridge and lasso outcome regression models, respectively, as a function of the outcome hyperparameter λ. The purple dotted lines show the values of λ chosen via cross validation, and the corresponding values of the ℓ_2 and ℓ_0 norms. Figures I.10b and I.10d show the corresponding norm for the unaugmented and balancing weights estimators, "double ridge" and "double lasso," respectively. For both, the norms for the augmented estimators are always at least as large as the norms for the outcome model alone. While the patterns are qualitatively the same, the behavior for ℓ_∞ balancing does not correspond to traditional undersmoothing, since the union of non-zero coefficients of the outcome and weighting models *cannot* generally be recovered by changing the hyperparameter of the lasso outcome model.

I.5 Semi-Synthetic Bias vs Variance

For the Lalonde long data, we use the same semi-synthetic strategy as in Appendix I, and then plot the analytical design-conditional bias and variance in Figure I.11. In this plot, we fix the (analytical) prediction-optimal value of λ, and then sweep the riesz regularization hyperparameter δ. Note that the variance dominates, and there's an especially large non-linearity when δ is close to zero.

J Empirical Bayes Interpretation of Augmented ℓ_2 Balancing Weights

This section discusses possible Empirical Bayes interpretations of augmented ℓ_2 balancing weights. We begin with the simple case with fixed outcome model coefficients, before exploring models that incorporate the uncertainty in these coefficients. While plausible, we do not find this interpretation particularly compelling, and instead point interested readers to recent work on weighting and Bayesian double robustness, especially Murray and Feller (2024).

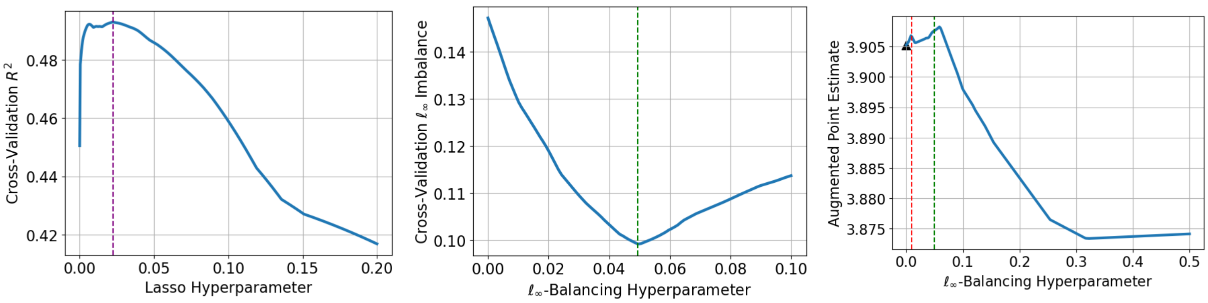

(a) Lasso outcome model

(b) ℓ_∞ balancing

(c) Augmented estimate

Figure I.4: Lasso-augmented ℓ_∞ balancing weights ("double lasso") for IHDP with the original 25 covariates. Panel (a) shows the 10-fold cross-validated R^2 for the Lasso-penalized regression of Y_p on Φ_p among control units across the hyperparameter λ; the purple dotted line shows the CV-optimal value. Panel (b) shows the 10-fold cross-validated imbalance for ℓ_∞ balancing weights across the hyperparameter δ; the green dotted line shows the CV-optimal value. Panel (c) shows the point estimate for the augmented estimator across the weighting hyperparameter δ; the black triangle corresponds to the OLS point estimate, the dotted green line corresponds to cross-validated imbalance, and the dotted red line corresponds to cross-validated Riesz loss.

J.1 Fixed outcome coefficients

We initially consider the straightforward special case where the outcome model coefficients $\hat{\beta}_{\text{reg}}$ are considered fixed, for example if obtained from an entirely different sample.

To fix ideas, consider a basic Bayesian linear model for a single data point i with covariates $x \in \mathbb{R}^J$ and outcome $y \in \mathbb{R}$:

$$y_i = \beta^\top x_i + \varepsilon_i, \quad \varepsilon_i \sim N(0,1).$$

As in the main text, we assume that the covariance matrix is diagonal with entries $\sigma_1^2, \ldots, \sigma_J^2$.

We briefly review the Bayesian interpretation of ridge regression as the posterior mean of β corresponding to the following prior:

$$\beta_j \mid \tau^2 \sim N(0, \tau^2),$$

with hyperparameter τ^2. The posterior for β_j is conditionally Normal:

$$\beta_j \mid \tau^2 \sim N\left(\frac{\tau^2}{\tau^2 + \frac{1}{\sigma_j^2}}\hat{\beta}_{ols,j}, \frac{1}{\frac{1}{\tau^2} + \sigma_j^2}\right)$$

where the posterior mean equals:

$$\left(\frac{\sigma_j^2}{\sigma_j^2 + \frac{1}{\tau^2}}\right)\hat{\beta}_{ols,j} + \left(\frac{\frac{1}{\tau^2}}{\sigma_j^2 + \frac{1}{\tau^2}}\right)0 = \left(\frac{\sigma_j^2}{\sigma_j^2 + \frac{1}{\tau^2}}\right)\hat{\beta}_{ols,j}.$$

To recover the augmented ℓ_2 balancing weights form, we simply center the prior for β_j at the fixed $\hat{\beta}_{\text{reg},j}$:

$$\beta_j \mid \tau^2 \sim N(\hat{\beta}_{reg,j}, \tau^2)$$

The posterior mean is then equal to:

$$\left(\frac{\sigma_j^2}{\sigma_j^2 + \frac{1}{\tau^2}}\right)\hat{\beta}_{ols,j} + \left(\frac{\frac{1}{\tau^2}}{\sigma_j^2 + \frac{1}{\tau^2}}\right)\hat{\beta}_{reg,j}$$

Setting $\tau^2 = \frac{1}{\delta}$ recovers the augmented ℓ_2 result from the main text.

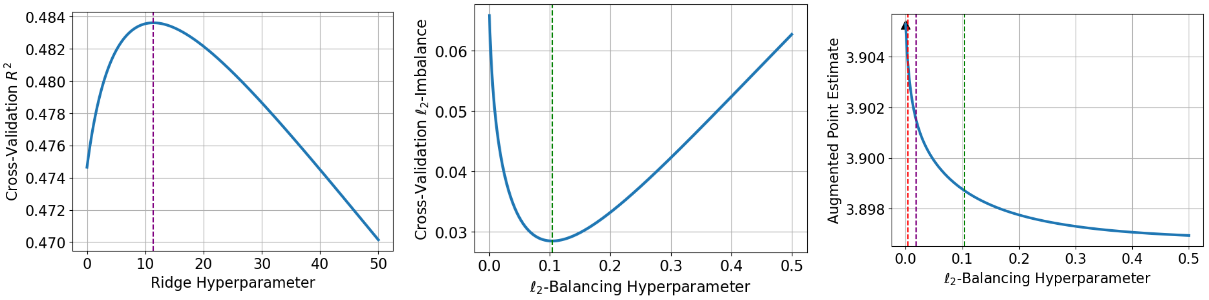

(a) Ridge outcome model

(b) ℓ_2 balancing

(c) Augmented estimate

Figure I.5: Ridge-augmented ℓ_2 balancing weights ("double ridge") for IDHP with the original 25 covariates. Panel (a) shows the 10-fold cross-validated R^2 for the Ridge-penalized regression of Y_p on Φ_p among control units across the hyperparameter λ; the purple dotted line shows the CV-optimal value. Panel (b) shows the 10-fold cross-validated AutoDML loss (6) for ℓ_2 balancing weights across the hyperparameter δ; the green dotted line shows the CV-optimal value. Panel (c) shows the point estimate for the augmented estimator across the weighting hyperparameter δ; the black triangle corresponds to the OLS point estimate, the green dotted line corresponds to cross-validated balance, the red dotted line corresponds to cross-validated Riesz loss, and the purple dotted line corresponds to the cross-validated ridge outcome hyperparameter.

J.2 Accounting for uncertainty in outcome model coefficients via global-local shrinkage priors

We now instead consider an Empirical Bayes interpretation of the "double ridge" form:

$$\left(\frac{\sigma_j^2}{\sigma_j^2 + \frac{\delta\lambda}{\sigma_j^2+\lambda+\delta}}\right)\hat{\beta}_{ols,j}.$$

Note that we cannot simply replace $\hat{\beta}_{reg,j}$ in the derivation above with $\left(\frac{\sigma_j^2}{\sigma_j^2+\lambda}\right)\hat{\beta}_{ols,j}$ since this ignores the uncertainty in estimating these coefficients.

J.2.1 Scale mixture of Normals

Again consider a general Bayesian linear model for a single data point i:

$$y_i = \beta^\top x_i + \varepsilon_i, \quad \varepsilon_i \sim N(0,1)$$

with the following general class of *global-local shrinkage priors* (Polson and Scott, 2010):

$$\begin{aligned}\beta_j \mid \xi_j, \tau, \sigma_j &\sim N(0, \tau^2\xi_j^2)\\ \xi_j &\sim p(\xi)\\ \tau &\sim p(\tau),\end{aligned}$$

where τ is called the *global* parameter, and ξ_j is called the *local* parameter. To simplify exposition, we again assume that the covariates are uncorrelated with zero mean and variances $\sigma_1^2, \ldots, \sigma_J^2$ (e.g., the principal components). As above, the posterior for β_j is conditionally Normal:

$$\beta_j \mid \tau^2, \xi_j, \sigma_j \sim N\left(\frac{\tau^2\xi_j^2}{\tau^2\xi_j^2 + \frac{1}{\sigma_j^2}}\hat{\beta}_j, \frac{1}{\frac{1}{\tau^2\xi_j^2} + \sigma_j^2}\right),$$

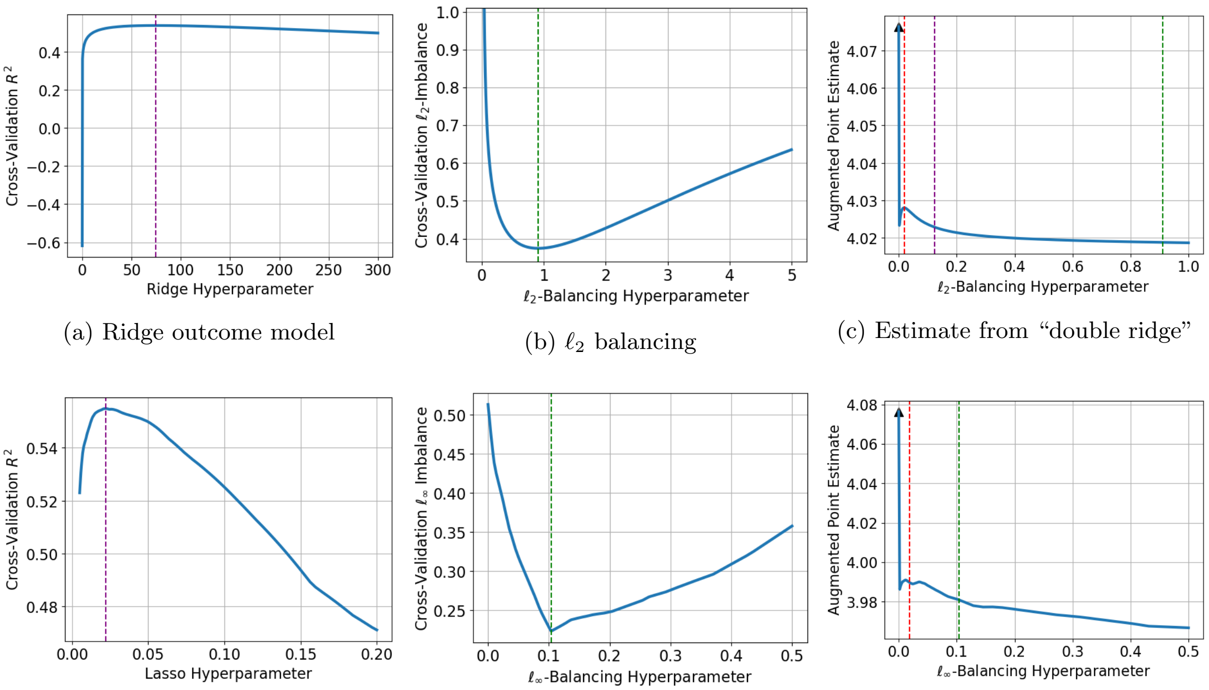

(a) Ridge outcome model

(b) ℓ_2 balancing

(c) Estimate from "double ridge"

(d) Lasso outcome model

(e) ℓ_∞ balancing

(f) Estimate from "double lasso"

Figure I.6: Augmented balancing weights estimates for the IHDP data set with the expanded set of 193 features; the top row shows ridge-augmented ℓ_2 balancing, and the bottom row shows lasso-augmented ℓ_∞ balancing. Panels (a) and (d) show the 3-fold cross-validated R^2 for the ridge- and laso-penalized regression of Y_p on Φ_p among control units across the hyperparameter λ; the purple dotted lines show the CV-optimal value for each. Panel (b) and (e) show the 10-fold cross-validated AutoDML loss (6) for ℓ_2 and ℓ_∞ balancing weights across the hyperparameter δ; the green dotted lines show the CV-optimal value for each. Panels (c) and (f) show the point estimates for the augmented estimators across the weighting hyperparameter δ; the black triangles correspond to the OLS point estimate; the green and red dotted lines correspond to the cross-validated balance and Riesz loss respectively; the purple line corresponds to the cross-validated ridge hyperparameter (for $\delta = \hat{\lambda}$).

where $\hat{\beta}$ is the usual OLS estimate.

Let $\tilde{\beta}_j$ be the posterior mean. We can write this in the following canonical shrinkage form:

$$\tilde{\beta}_j = (1 - \kappa_j)\hat{\beta}_j,$$

where

$$\kappa_j = \frac{1}{1 + \tau^2 \xi_j^2 \sigma_j^2}.$$

Here $\kappa_j = 0$ implies no shrinkage and $\kappa_j = 1$ implies complete shrinkage. Piironen and Vehtari (2017) show that all regression priors that are scale-mixture of Normals can be written in this form. Some prominent examples include:

- **Ridge.** Standard ridge regression sets $\xi_j^2 = 1$.
- **Horseshoe prior.** Here $\xi_j \sim \mathrm{C}^+(0, 1)$, where C^+ denotes the half-Cauchy density (Carvalho et al., 2010).

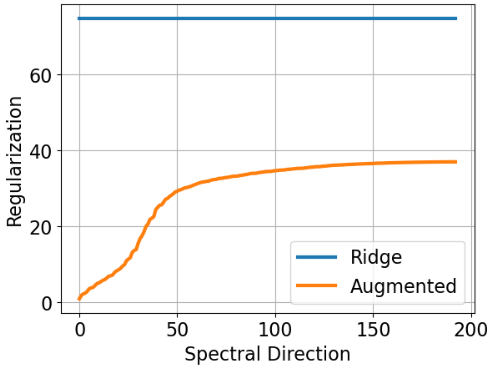

(a) Adaptive regularization

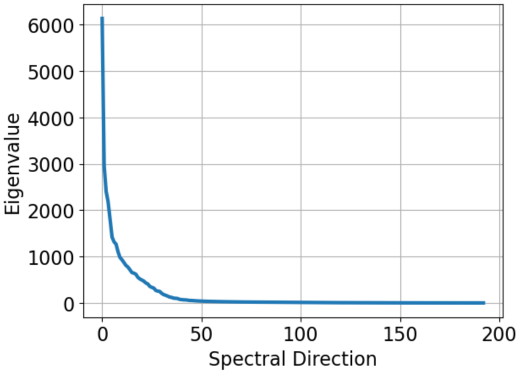

(b) Eigenvalues of $\Phi_p^\top \Phi_p$

Figure I.7: Single and double ridge regression applied to the "high-dimensional" version of the IHDP data set. Panel (a) shows λ_j, the single ridge hyperparameter, and γ_j, the implied double ridge hyperparameter, as functions of the spectral direction j. Panel (b) shows the corresponding eigenvalues.

- **Spike-and-slab priors.** When the spike is a point mass at 0, the resulting prior also has this shrinkage form.
- **Zellner's g prior.** While not a proper prior, Zellner's g prior has the same canonical form, with $\xi_j^2 = \frac{1}{\sigma_j^2}$ in the case of a diagonal covariance matrix, with corresponding $\kappa_j = \frac{1}{1+\tau^2}$.

J.2.2 Inverse-Gamma Gamma Prior

We focus on a flexible hierarchical prior known as the *Inverse Gamma-Gamma* prior (Bai and Ghosh, 2017) or, equivalently, *Beta-prime* prior (Armagan et al., 2011):

$$\begin{aligned} \beta_j \mid \xi_j, \tau &\sim N(0, \tau^2 \xi_j^2) \\ \xi_j^2 &\sim \text{Gamma}(a, 1) \\ \frac{1}{\tau^2} &\sim \text{Gamma}(b, 1) \end{aligned}$$

This formulation guarantees that the product $\xi^2\tau^2 \sim \text{Beta-prime}(a, b)$, also known as the inverse-Beta distribution. For $\sigma_j^2 = 1$, we then have $(1 - \kappa_j) \equiv \frac{\xi^2\tau^2}{1+\xi^2\tau^2} \sim \text{Beta}(a, b)$, and by symmetry, $\kappa_j \sim \text{Beta}(b, a)$; with the expectation of $\kappa_j = \frac{b}{a+b} = \frac{1}{1+\frac{a}{b}}$. The horseshoe is a special case with $a = b = 1/2$. Incorporating σ_j^2, the implied prior distribution for the shrinkage factor is $\kappa_j \sim \text{Beta}(b, a\sigma_j^2)$, with expectation $\frac{b}{a\sigma_j^2+b} = \frac{1}{1+\frac{a\sigma_j^2}{b}}$.

J.2.3 Application to double ridge

With a bit of algebra, we can write the shrinkage factor for double ridge as:

$$\kappa_j^* = \frac{1}{1 + \frac{\sigma_j^2(\sigma_j^2+\lambda+\delta)}{\delta\lambda}}$$

This corresponds to an Inverse Gamma-Gamma prior with $a = \sigma_j^2 + \lambda + \delta$ and $b = \delta\lambda$.

$$\begin{aligned} \beta_j \mid \xi_j, \tau &\sim N(0, \tau^2 \xi_j^2) \\ \xi_j^2 \mid \delta, \lambda, \sigma_j^2 &\sim \text{Gamma}(\sigma_j^2 + \lambda + \delta, 1) \\ \frac{1}{\tau^2} \mid \delta, \lambda &\sim \text{Gamma}(\delta\lambda, 1), \end{aligned}$$

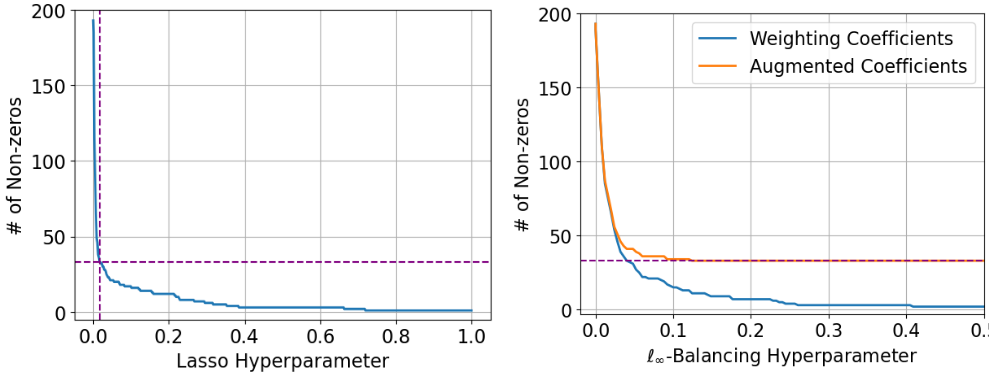

(a) ℓ_0 "norm" for ridge outcome model (b) ℓ_0 "norm" for weighting and double lasso

Figure I.8: Undersmoothing in ℓ_0 "norm" for augmented balancing weights applied to the "high dimesional" version of the IHDP data set. Panels (a) shows the number of non-zero covariates (the ℓ_0 "norm") for lasso regression of Y_p on Φ_p among control units. Panels (b) shows the number of non-zero covariates for the weighting model and augmented coefficients. The dotted purple line is the outcome model hyperparameter chosen via cross validation.

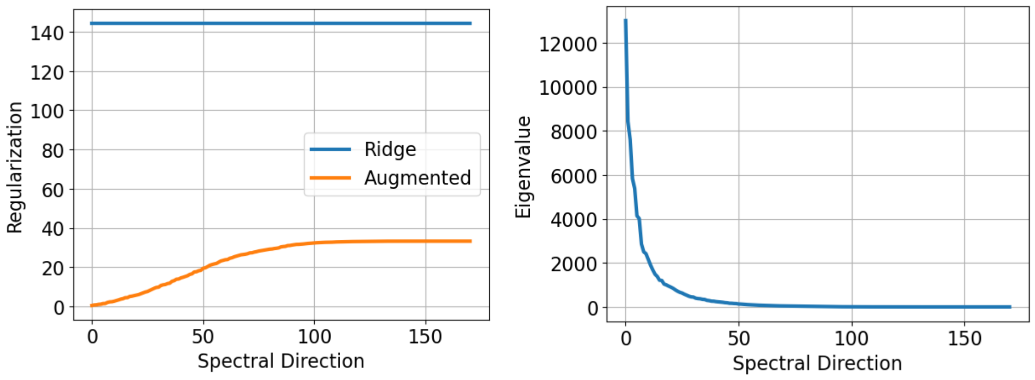

(a) Adaptive regularization (b) Eigenvalues of $\Phi_p^\top \Phi_p$

Figure I.9: Single and double ridge regression applied to the "high-dimensional" version of the LaLonde (1986) data set. Panel (a) shows λ_j, the single ridge hyperparameter, and γ_j, the implied double ridge hyperparameter, as functions of the spectral direction j. Panel (b) shows the corresponding eigenvalues.

where the implied prior for κ_j^* is:

$$\kappa_j^* \mid \delta, \lambda, \sigma_j^2 \sim \text{Beta}(\delta\lambda, \sigma_j^2(\sigma_j^2 + \delta + \lambda))$$

As expected, δ and λ have symmetric roles — the hyperprior distributions only depend on their product and sum. We can assess the behavior of this prior in two extreme cases. First, when $\delta \to \infty$ or $\lambda \to \infty$, the prior on κ_j^* tends to Beta(b^*, a^*) with $a^* \to \infty, b^* \to \infty$. The corresponding expectation of κ_j^* tends to 1, which implies full shrinkage, $\tilde{\beta}_j = 0$. Second, when $\delta \to 0$ or $\lambda \to 0$, the prior on κ_j^* tends to Beta(b^*, a^*) with $a^* \to \sigma_j^4, b^* \to 0$. The corresponding expectation of κ_j^* tends to 0, or no shrinkage, $\tilde{\beta}_j = \hat{\beta}_j$.

As Figure J.12 shows, the shrinkage depends strongly on the eigenvalue σ_j^2 and the hyperparameters δ and λ. Holding δ and λ fixed, κ_j is decreasing in σ_j^2 — i.e., there is less shrinkage for larger σ_j^2. Holding σ_j^2 fixed,

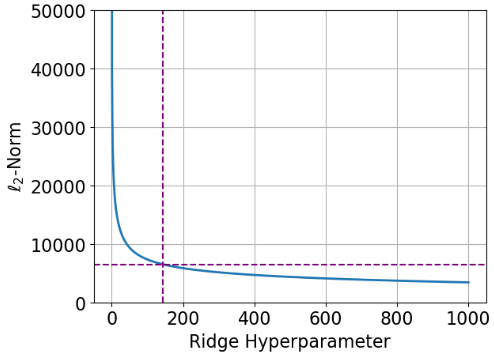

(a) ℓ_2 norm for ridge outcome model

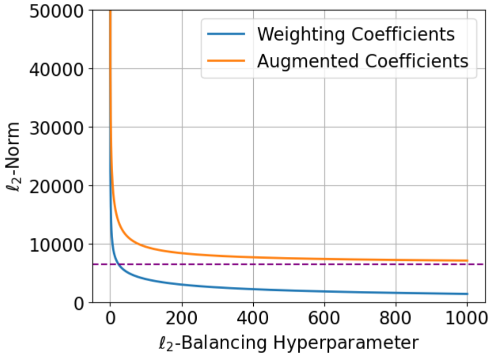

(b) ℓ_2 norm for weighting and double ridge

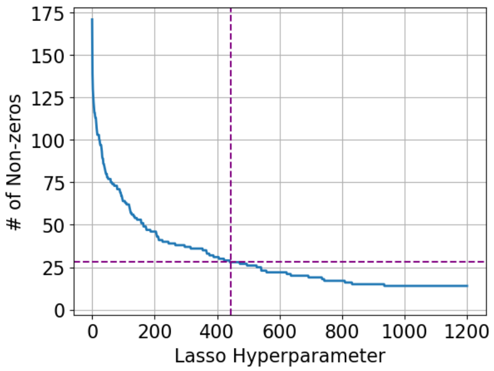

(c) ℓ_0 "norm" for lasso outcome model

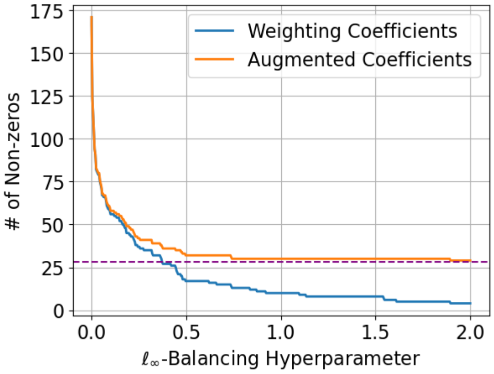

(d) ℓ_0 "norm" for weighting and double lasso

Figure I.10: Undersmoothing in norms for augmented balancing weights applied to the "high dimensional" version of the LaLonde (1986) data set. Panels (a) and (c) show the ℓ_2 norm and the number of non-zero covariates (the ℓ_0 "norm") for the ridge and lasso regression, respectively, of Y_p on Φ_p among control units. Panels (b) and (d) show the ℓ_2 and number of non-zero covariates for the corresponding unaugmented and augmented balancing weights estimators. The dotted purple line is the outcome model hyperparameter chosen via cross validation. The norms for the augmented estimators are always at least as large as for the outcome model alone.

κ_j is increasing δ and λ — i.e., as the hyperparameters increase, both the outcome and weighting models move closer to uniform weights and greater shrinkage.

Connection to Student t shrinkage. If we drop the hyperprior on ξ^2 and set $\xi_j^2 = 1$, this reduces to:

$$\beta_j \mid \tau \sim N(0, \tau^2)$$
$$\frac{1}{\tau^2} \sim \text{Gamma}(\delta\lambda, 1)$$

This yields a student t posterior for β_j.

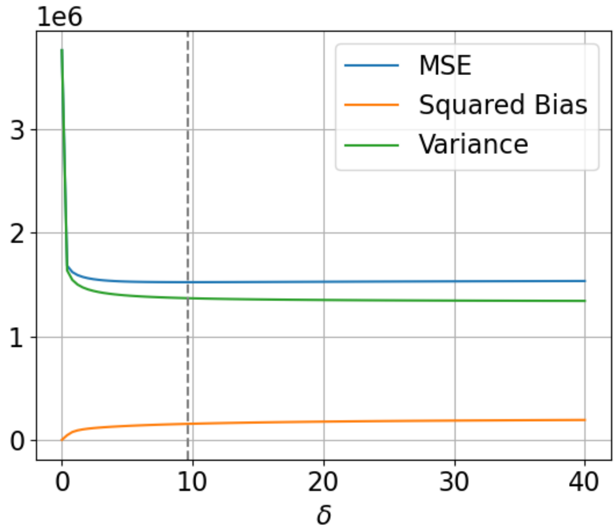

Figure I.11: The exact, finite sample, bias, variance, and mean-squared error for the semi-synthetic LaLonde DGP as a function of the riesz regularization hyperparameter δ.

Connection to Normal-Gamma prior. If we instead drop the hyperprior on τ^2 and set $\tau^2 = 1$, this reduces to:

$$\beta_j \mid \xi_j, \tau \sim N(0, \xi_j^2)$$
$$\xi_j^2 \sim \text{Gamma}(\sigma_j^2 + \lambda + \delta, 1)$$

which is a Normal-Gamma prior, where the hyperparameter depends on σ_j^2. Here the prior mean for ξ_j^2 is simply $\sigma_j^2 + \delta + \lambda$, which is increasing in ξ_j^2.

J.3 Connection to Zellner's g prior

Finally, we can connect this approach to generalization of Zellner's g prior known as *adaptive powered correlation prior* (Krishna et al., 2009), which has the form:

$$\beta_j \mid g \sim N\left(0, \frac{1}{g}(X'X)^{\nu}\right)$$

with additional hyperparameter ν. Zellner's g prior corresponds to $\nu = -1$, and standard ridge regression corresponds to $\nu = 0$. We are interested in the "inverted g prior" with $\nu = +1$. Since we focus on diagonal covariance, we can re-write the prior as:

$$\beta_j \mid g \sim N\left(0, \frac{1}{g}\sigma_j^{2\nu}\right)$$

Then the posterior mean is:

$$\tilde{\beta} = \left(\frac{\frac{1}{g\sigma_j^{2\nu}}}{\frac{1}{g\sigma_j^{2\nu}} + \frac{1}{\sigma_j^2}}\right)\hat{\beta}_j$$
$$= \left(\frac{\sigma_j^{(2\nu+2)}}{\sigma_j^{(2\nu+2)} + g}\right)\hat{\beta}_j$$

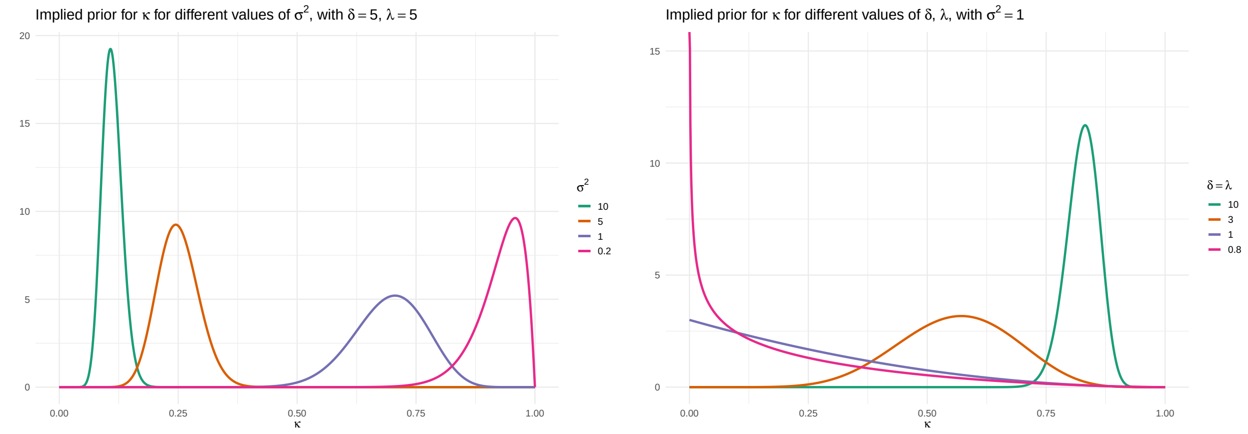

Figure J.12: Implied prior on κ for: (left) varying σ_j^2, with $\delta = 5$, $\lambda = 5$; (right) varying $\delta = \lambda$, with $\sigma_j^2 = 1$.

Special cases include:

- **Zellner's g prior:** $(\nu = -1)$

$$\tilde{\beta} = \left(\frac{1}{1+g}\right)\hat{\beta}_j$$

- **Ridge regression:** $(\nu = 0)$

$$\tilde{\beta} = \left(\frac{\sigma_j^2}{\sigma_j^2 + g}\right)\hat{\beta}_j$$

- **Inverted g prior:** $(\nu = +1)$

$$\tilde{\beta} = \left(\frac{\sigma_j^4}{\sigma_j^4 + g}\right)\hat{\beta}_j$$

We can view the double ridge as a regularized form of this inverted g prior, with $\nu = +1$ and $g = \delta\lambda$.

K Additional Proofs

Closed forms for ℓ_2 and exact balancing weights. We derive the closed form for ℓ_2 balancing weights with parameter δ (with exact balance following as a special case). The optimization problem:

$$\min_{w \in \mathbb{R}^n} \|w^\top \Phi_p - \bar{\Phi}_q\|_2^2 + \delta \|w\|_2^2 = w^\top \Phi_p \Phi_p^\top w - 2w^\top \Phi_p \bar{\Phi}_q + \delta w^\top w$$

has first order condition:

$$2(\Phi_p \Phi_p^\top + \delta I_n)w - 2\Phi_p \bar{\Phi}_q = 0,$$

which gives the solution:

$$\begin{aligned} w^* &= (\Phi_p \Phi_p^\top + \delta I_n)^\dagger \Phi_p \bar{\Phi}_q \\ &= \Phi_p (\Phi_p^\top \Phi_p + \delta I_d)^\dagger \bar{\Phi}_q. \end{aligned}$$

□

Proof of Proposition 4.1. We apply Proposition 3.2. We have $a_j^\delta = \hat{\Phi}_{q,j}^\delta / \bar{\Phi}_{q,j}^\delta$. Then:

$$\hat{\Phi}_q^\delta = \hat{w}_{\ell_2}^\delta \Phi_p = \overline{\Phi}_q (\Phi_p^\top \Phi_p + \delta I)^{-1} \Phi_p^\top \Phi_p.$$

Since we have assumed that $\Phi_p^\top \Phi_p$ is diagonal, with jth diagonal entry, σ_j^2, we have:

$$\hat{\Phi}_{q,j}^\delta = \left(\frac{\sigma_j^2}{\sigma_j^2 + \delta} \right) \bar{\Phi}_{q,j}.$$

Plugging this back into a_j^δ completes the proof. □

Proof of Proposition 4.3. Applying Proposition Proposition 4.1:

$$\begin{aligned}
\hat{\beta}_{\ell_2,j} &= \left(\frac{\sigma_j^2}{\sigma_j^2+\delta}\right)\hat{\beta}_{\text{ols,j}} + \left(\frac{\delta}{\sigma_j^2+\delta}\right)\hat{\beta}^{\lambda}_{\text{ridge,j}} \\
&= \left(\frac{\sigma_j^2}{\sigma_j^2+\delta}\right)\hat{\beta}_{\text{ols,j}} + \left(\frac{\delta}{\sigma_j^2+\delta}\right)\left(\frac{\sigma_j^2}{\sigma_j^2+\lambda}\right)\hat{\beta}_{\text{ols},j} \\
&= \frac{\sigma_j^2(\sigma_j^2+\lambda+\delta)}{(\sigma_j^2+\delta)(\sigma_j^2+\lambda)}\hat{\beta}_{\text{ols,j}}
\end{aligned}$$

Then taking:

$$\frac{\sigma_j^2(\sigma_j^2+\lambda+\delta)}{(\sigma_j^2+\delta)(\sigma_j^2+\lambda)} = \frac{\sigma_j^2}{\sigma_j^2+\gamma_j}$$

and solving for γ_j gives:

$$\gamma_j \coloneqq \frac{\delta\lambda}{\sigma_j^2+\lambda+\delta}$$

which completes the proof. □

Proof of Proposition 5.1. We begin with the constrained form of the balancing problem:

$$\begin{aligned}
&\min_{w\in\mathbb{R}^n} \|w\|_2^2 \\
&\text{such that } \left\|w\Phi_p - \bar{\Phi}_q\right\|_\infty \le \delta.
\end{aligned}$$

Note that we can rewrite the norm constraint as two vector-valued linear constraints:

$$\begin{aligned}
w\Phi_p &\preceq \bar{\Phi}_q + \delta \\
-w\Phi_p &\preceq -\bar{\Phi}_q + \delta,
\end{aligned}$$

which results in the Lagrangian,

$$\mathcal{L}(w,\mu,\nu) = \|w\|_2^2 + \mu^\top\left(w\Phi_p - \bar{\Phi}_q - \delta\right) - \nu^\top\left(w\Phi_p - \bar{\Phi}_q + \delta\right).$$

The first-order conditions for the optimal w^*, μ^*, ν^* are:

$$\begin{aligned}
w^* &= -\frac{1}{2}\left(\Phi_p\mu^* - \Phi_p\nu^*\right) \\
\mu_j^*\left(w^*\Phi_{p,j} - \bar{\Phi}_{q,j} - \delta\right) &= 0, \forall j \\
\nu_j^*\left(w^*\Phi_{p,j} - \bar{\Phi}_{q,j} + \delta\right) &= 0, \forall j \\
\mu_j^*, \nu_j^* &\ge 0, \forall j
\end{aligned}$$

plus the linear constraints on $w^*\Phi_p$. Note that the linear constraints plus the complimentary slackness conditions imply that one of three mutually-exclusive cases holds for each covariate. Case 1: $w^*\Phi_{p,j} = \bar{\Phi}_{q,j} - \delta$, in which case $\mu_j^* = 0$. Case 2: $w^*\Phi_{p,j} = \bar{\Phi}_{q,j} + \delta$, in which case $\nu_j^* = 0$. Or Case 3: $w^*\Phi_{p,j} \in (\bar{\Phi}_{q,j} - \delta, \bar{\Phi}_{q,j} + \delta)$, in which case $\mu_j^* = \nu_j^* = 0$.

Define:

$$\theta_j^* \coloneqq \begin{cases} 0 & \text{if } w^*\Phi_{p,j} \in (\bar{\Phi}_{q,j} - \delta, \bar{\Phi}_{q,j} + \delta) \\ -\mu_j^*/2 & \text{if } w^*\Phi_{p,j} = \bar{\Phi}_{q,j} + \delta \\ \nu_j^*/2 & \text{if } w^*\Phi_{p,j} = \bar{\Phi}_{q,j} - \delta. \end{cases}$$

Then we have $w^* = \Phi_p \theta^*$ from the first-order condition, and thus $w^* \Phi_p = (\Phi_p^\top \Phi_p)\theta^*$. Using the fact that $(\Phi_p^\top \Phi_p)$ is diagonal, we get $w^* \Phi_{p,j} = \sigma_j^2 \theta_j^*$.

Finally, we can plug this into the three cases that define θ^*. First, $\theta_j^* = 0$ when

$$\begin{aligned} &\sigma_j^2 \theta_j^* \in (\bar{\Phi}_q - \delta, \bar{\Phi}_q + \delta) \\ \implies &0 \in (\bar{\Phi}_q - \delta, \bar{\Phi}_q + \delta) \\ \implies &\bar{\Phi}_q \in (-\delta, \delta). \end{aligned}$$

Second, $\theta_j^* = -\mu_j^*/2$ when $\sigma_j^2 \theta_j^* = \bar{\Phi}_{q,j} + \delta$, which implies $\mu_j^* = -2(\bar{\Phi}_{q,j} + \delta)/\sigma_j^2$. We then apply the dual variable constraint:

$$\begin{aligned} &\mu_j^* \geq 0 \\ \implies &-2(\bar{\Phi}_{q,j} + \delta)/\sigma_j^2 \geq 0 \\ \implies &\bar{\Phi}_{q,j} \leq -\delta. \end{aligned}$$

Third, $\theta_j^* = \nu_j^*/2$ when $\sigma_j^2 \theta_j^* = \bar{\Phi}_{q,j} - \delta$, which implies $\nu_j^* = 2(\bar{\Phi}_{q,j} - \delta)/\sigma_j^2$. We then apply the dual variable constraint:

$$\begin{aligned} &\nu_j^* \geq 0 \\ \implies &2(\bar{\Phi}_{q,j} - \delta)/\sigma_j^2 \geq 0 \\ \implies &\bar{\Phi}_{q,j} \geq \delta. \end{aligned}$$

Putting the cases together we get:

$$\theta_j^* := \begin{cases} 0 & \text{if } \bar{\Phi}_q \in (-\delta, \delta) \\ (\bar{\Phi}_{q,j} + \delta)/\sigma_j^2 & \text{if } \bar{\Phi}_{q,j} \leq -\delta. \\ (\bar{\Phi}_{q,j} - \delta)/\sigma_j^2 & \text{if } \bar{\Phi}_{q,j} \geq \delta. \end{cases}$$

This is exactly the soft-thresholding operator, which completes the proof. ☐

Proof of Proposition 5.2. To obtain this result from the general form of $a_j^\delta = \widehat{\Delta}_j/\Delta_j$ in Proposition 3.2, notice that the implied feature shift, $\widehat{\Delta}_j = \hat{\Phi}_{q,j}^\delta - \bar{\Phi}_{p,j} = \mathcal{T}_\delta(\bar{\Phi}_{q,j} - \bar{\Phi}_{p,j})$ is:

$$\widehat{\Delta}_j = \begin{cases} 0 & \text{if } |\Delta_j| < \delta \\ \Delta_j - \delta & \text{if } \Delta_j > \delta \\ \Delta_j + \delta & \text{if } \Delta_j < -\delta \end{cases}.$$

Thus, for instance, $\frac{\widehat{\Delta}_j}{\Delta} = \frac{\Delta_j - \delta}{\Delta_j} = 1 - \frac{\delta}{\Delta_j}$ when $\Delta_j > \delta$. ☐

Proof of Proposition 5.3. The result follows immediately from Proposition 5.2. ☐

Proof of Proposition L.1. Rewriting the definition of γ_n with $\lambda_n = \delta_n$, we have

$$\gamma_n = \frac{\lambda_n^2}{\sigma^2 + 2\lambda_n^2} = \frac{1}{\sigma^2 \lambda_n^{-2} + 2\lambda_n^{-1}}.$$

Because $\sigma^2 x^2 + 2x = O(x^2)$ as a function of x, and because λ_n^{-1} is monotonically increasing, $\sigma^2 \lambda_n^{-2} + 2\lambda_n^{-1} = O(\lambda_n^{-2})$. And $\lambda_n^{-2} = O(\sigma^2 \lambda_n^{-2} + 2\lambda_n^{-1})$ because $\sigma^2 \geq 0$ and $\lambda_n^{-1} > 0$. Thus $\sigma^2 \lambda_n^{-2} + 2\lambda_n^{-1} \asymp \lambda_n^{-2}$.

Finally, note that for any two functions of n, f_n and g_n,

$$f_n \asymp g_n \iff f_n^{-1} \asymp g_n^{-1},$$

and therefore,

$$\gamma_n \asymp \lambda_n^2.$$

□

L Additional Details for Asymptotic Results

Our setup for the RKHS follows Singh (2024). First, assume that the space $\mathcal{X} \times \mathcal{Z}$ is Polish. Let $\mathcal{H}$ be an RKHS on $\mathcal{X} \times \mathcal{Z}$ with corresponding kernel k satisfying standard regularity conditions (Singh, 2024, Assumption 5) and let η_j, φ_j denote the eigenvalues and eigenfunctions respectively of its kernel integral operator under p. Next, assume that the eigenvalues satisfy the decay condition $\eta_j \leq Cj^{-b}$ for some $b > 1$ and a constant C. The parameter b encodes information on the effective dimension of $\mathcal{H}$. For a bounded kernel, $b > 1$ (Fischer and Steinwart, 2020): the case where $b = \infty$ corresponds to a finite-dimensional RKHS; for the case with $1 < b < \infty$, the η_j must decay at a polynomial rate.

We then assume that for some $c \in [1, 2]$, the outcome function $m(x, z)$ belongs to the set:

$$\mathcal{H}^c := \left\{ f = \sum_{j=1}^{\infty} a_j \varphi_j : \sum_{j=1}^{\infty} \frac{a_j^2}{\eta_j^c} < \infty \right\} \subset \mathcal{H}, \tag{18}$$

where c encodes additional *smoothness* of the conditional expectation. If $c = 1$, then by the spectral decomposition of the RKHS, Equation (18) is equivalent to requiring $m \in \mathcal{H}$; choosing larger values of c corresponds to m being a smoother element of $\mathcal{H}$, with a "saturation effect" kicking in for $c > 2$ (Bauer et al., 2007). Varying b (the effective dimension of the RKHS) and c (the additional smoothness of the outcome function) changes the optimal rates for regression, with larger values of both corresponding to faster rates of convergence.

As noted in the main text, we will begin with the assumption where the minimal riesz representer also belongs to $\mathcal{H}^c$ — this appears to be a non-trivial assumption, because the closure condition on the minimal riesz representer is in terms of the L_2 distance instead of the Hilbert space distance. However, it's possible that some version of this result holds without making any assumptions. For example, it has been shown that one can achieve optimal undersmoothing for RKHS plug-in estimates of linear functionals just by changing the outcome schedule λ_n, which doesn't appear to (separately) require knowledge of the smoothness of the Riesz representer. Note that we can relax the assumption that the riesz representer is in $\mathcal{H}^c$, following Singh (2024), but in general the outcome model and riesz representer would then have *different* smoothness parameters, c. Under the conditions above, combining the rate results from Fischer and Steinwart (2020) and Singh (2024) with Theorem 4.2 of Chernozhukov et al. (2022c), we get that an augmented estimator combining kernel balancing weights and a kernel ridge regression base learner is root-n consistent and asymptotically normal.

Following Caponnetto and De Vito (2007), Theorem 2 of Singh (2024) use hyperparameter schedules, which depend on the effective dimension b and smoothness c:

$$\lambda_n = \delta_n = \begin{cases} n^{-1/2} & \text{if } b = \infty \\ n^{-\frac{b}{bc+1}} & \text{if } b \in (1, \infty), \quad c \in (1, 2] \\ (n/\log(n))^{-b/(b+1)} & \text{if } b \in (1, \infty), \quad c = 1 \end{cases},$$

We can compute the implied augmented hyperparameter sequence γ_n using the following proposition.

Proposition L.1. *Let $\lambda_n > 0$ be any monotonically decreasing function of n and let $\delta_n = \lambda_n$. Then:*

$$\gamma_n := \frac{\lambda_n \delta_n}{\sigma^2 + \lambda_n + \delta_n} \asymp \lambda_n^2.$$

The standard ridge regression case corresponds to the finite-dimensional setting with $b = \infty$. When $c > 1$, the optimal rate for λ_n is $n^{-\frac{b}{bc+1}}$; the implied hyperparameter is then order $n^{-2b/bc+1} \in (n^{-2}, n^{-2/3})$ for $c \in (1, 2]$ and $b \in (1, \infty)$. Whether or not this smooths more than n^{-1} therefore depends on the relationship between the effective dimension b and the smoothness c. In particular, the implied hyperparameter goes to zero at a slower rate than n^{-1} whenever $c \geq 2 - \frac{1}{b}$. It is unclear whether the rates we find here are the only undersmoothed rates that will yield efficiency for fixed b and c; we leave a thorough investigation to future work.

References

A. Abadie, A. Diamond, and J. Hainmueller. Synthetic control methods for comparative case studies: Estimating the effect of california's tobacco control program. *Journal of the American statistical Association*, 105(490):493–505, 2010.

A. Armagan, M. Clyde, and D. Dunson. Generalized beta mixtures of gaussians. *Advances in neural information processing systems*, 24, 2011.

S. Athey, G. W. Imbens, and S. Wager. Approximate residual balancing: debiased inference of average treatment effects in high dimensions. *Journal of the Royal Statistical Society: Series B (Statistical Methodology)*, 80(4):597–623, 2018.

R. Bai and M. Ghosh. The inverse gamma-gamma prior for optimal posterior contraction and multiple hypothesis testing. *arXiv preprint arXiv:1710.04369*, 2017.

P. L. Bartlett, P. M. Long, G. Lugosi, and A. Tsigler. Benign overfitting in linear regression. *Proceedings of the National Academy of Sciences*, 117(48):30063–30070, 2020.

F. Bauer, S. Pereverzev, and L. Rosasco. On regularization algorithms in learning theory. *Journal of complexity*, 23(1):52–72, 2007.

E. Ben-Michael, A. Feller, D. A. Hirshberg, and J. R. Zubizarreta. The balancing act in causal inference. *arXiv preprint arXiv:2110.14831*, 2021a.

E. Ben-Michael, A. Feller, and J. Rothstein. The augmented synthetic control method. *Journal of the American Statistical Association*, 116(536):1789–1803, 2021b.

D. A. Bruns-Smith and A. Feller. Outcome assumptions and duality theory for balancing weights. In *International Conference on Artificial Intelligence and Statistics*, pages 11037–11055. PMLR, 2022.

A. Caponnetto and E. De Vito. Optimal rates for the regularized least-squares algorithm. *Foundations of Computational Mathematics*, 7:331–368, 2007.

C. M. Carvalho, N. G. Polson, and J. G. Scott. The horseshoe estimator for sparse signals. *Biometrika*, 97 (2):465–480, 2010.

A. Chattopadhyay, C. H. Hase, and J. R. Zubizarreta. Balancing vs modeling approaches to weighting in practice. *Statistics in Medicine*, 39(24):3227–3254, 2020.

V. Chernozhukov, D. Chetverikov, M. Demirer, E. Duflo, C. Hansen, W. Newey, and J. Robins. Double/debiased machine learning for treatment and structural parameters. *The Econometrics Journal*, 21 (1):C1–C68, 01 2018.

V. Chernozhukov, W. Newey, V. M. Quintas-Martinez, and V. Syrgkanis. Riesznet and forestriesz: Automatic debiased machine learning with neural nets and random forests. In *International Conference on Machine Learning*, pages 3901–3914. PMLR, 2022a.

V. Chernozhukov, W. K. Newey, and R. Singh. Automatic debiased machine learning of causal and structural effects. *Econometrica*, 90(3):967–1027, 2022b.

V. Chernozhukov, W. K. Newey, and R. Singh. Debiased machine learning of global and local parameters using regularized riesz representers. *The Econometrics Journal*, 2022c.

J.-C. Deville and C.-E. Särndal. Calibration estimators in survey sampling. *Journal of the American statistical Association*, 87(418):376–382, 1992.

J. C. Duchi, P. W. Glynn, and H. Namkoong. Statistics of robust optimization: A generalized empirical likelihood approach. *Mathematics of Operations Research*, 46(3):946–969, 2021.

M. H. Farrell. Robust inference on average treatment effects with possibly more covariates than observations. *Journal of Econometrics*, 189(1):1–23, 2015.

S. Fischer and I. Steinwart. Sobolev norm learning rates for regularized least-squares algorithms. *The Journal of Machine Learning Research*, 21(1):8464–8501, 2020.

W. A. Fuller. Regression estimation for survey samples. *Survey Methodology*, 28(1):5–24, 2002.

B. S. Graham, C. C. de Xavier Pinto, and D. Egel. Inverse probability tilting for moment condition models with missing data. *The Review of Economic Studies*, 79(3):1053–1079, 2012.

A. Gretton, K. M. Borgwardt, M. J. Rasch, B. Schölkopf, and A. Smola. A kernel two-sample test. *The Journal of Machine Learning Research*, 13(1):723–773, 2012.

J. Hainmueller. Entropy balancing for causal effects: A multivariate reweighting method to produce balanced samples in observational studies. *Political analysis*, 20(1):25–46, 2012.

C. Harshaw, F. Sävje, D. Spielman, and P. Zhang. Balancing covariates in randomized experiments with the gram–schmidt walk design. *arXiv preprint arXiv:1911.03071*, 2019.

C. Hazlett. Kernel balancing. *Statistica Sinica*, 30(3):1155–1189, 2020.

J. K. Hellerstein and G. W. Imbens. Imposing moment restrictions from auxiliary data by weighting. *Review of Economics and Statistics*, 81(1):1–14, 1999.

J. L. Hill. Bayesian nonparametric modeling for causal inference. *Journal of Computational and Graphical Statistics*, 20(1):217–240, 2011.

K. Hirano, G. W. Imbens, and G. Ridder. Efficient estimation of average treatment effects using the estimated propensity score. *Econometrica*, 71(4):1161–1189, 2003.

D. A. Hirshberg, A. Maleki, and J. R. Zubizarreta. Minimax linear estimation of the retargeted mean. *arXiv preprint arXiv:1901.10296*, 2019.

K. Imai and M. Ratkovic. Covariate balancing propensity score. *Journal of the Royal Statistical Society: Series B: Statistical Methodology*, pages 243–263, 2014.

N. Kallus. Generalized optimal matching methods for causal inference. *J. Mach. Learn. Res.*, 21:62–1, 2020.

E. H. Kennedy. Semiparametric doubly robust targeted double machine learning: a review. *arXiv preprint arXiv:2203.06469*, 2022.

K. Kim, B. A. Niknam, and J. R. Zubizarreta. Scalable kernel balancing weights in a nationwide observational study of hospital profit status and heart attack outcomes. 2022.

P. Kline. Oaxaca-blinder as a reweighting estimator. *American Economic Review*, 101(3):532–37, 2011.

A. Krishna, H. D. Bondell, and S. K. Ghosh. Bayesian variable selection using an adaptive powered correlation prior. *Journal of statistical planning and inference*, 139(8):2665–2674, 2009.

R. J. LaLonde. Evaluating the econometric evaluations of training programs with experimental data. *The American economic review*, pages 604–620, 1986.

R. R. Lin, H. Z. Zhang, and J. Zhang. On reproducing kernel banach spaces: Generic definitions and unified framework of constructions. *Acta Mathematica Sinica, English Series*, 38(8):1459–1483, 2022.

Z. Lin and F. Han. On regression-adjusted imputation estimators of the average treatment effect. *arXiv preprint arXiv:2212.05424*, 2022.

A. Menon and C. S. Ong. Linking losses for density ratio and class-probability estimation. In *International Conference on Machine Learning*, pages 304–313. PMLR, 2016.

J. Murray and A. Feller. Bayesian causal models from a weighting perspective: Balance, bias, and double robustness. 2024.

W. K. Newey and J. R. Robins. Cross-fitting and fast remainder rates for semiparametric estimation. *arXiv preprint arXiv:1801.09138*, 2018.

W. K. Newey and R. J. Smith. Higher order properties of gmm and generalized empirical likelihood estimators. *Econometrica*, 72(1):219–255, 2004.

J. Piironen and A. Vehtari. Sparsity information and regularization in the horseshoe and other shrinkage priors. *Electronic Journal of Statistics*, 11(2):5018 – 5051, 2017. doi: 10.1214/17-EJS1337SI. URL https://doi.org/10.1214/17-EJS1337SI.

N. G. Polson and J. G. Scott. Shrink globally, act locally: Sparse bayesian regularization and prediction. *Bayesian statistics*, 9(501-538):105, 2010.

J. Qin and B. Zhang. Empirical-likelihood-based inference in missing response problems and its application in observational studies. *Journal of the Royal Statistical Society: Series B (Statistical Methodology)*, 69 (1):101–122, 2007.

J. Robins, M. Sued, Q. Lei-Gomez, and A. Rotnitzky. Comment: Performance of double-robust estimators when" inverse probability" weights are highly variable. *Statistical Science*, 22(4):544–559, 2007.

J. M. Robins, A. Rotnitzky, and L. P. Zhao. Estimation of regression coefficients when some regressors are not always observed. *Journal of the American statistical Association*, 89(427):846–866, 1994.

D. B. Rubin. Randomization analysis of experimental data: The fisher randomization test comment. *Journal of the American statistical association*, 75(371):591–593, 1980.

M. Rubinstein, A. Haviland, and D. Choi. Balancing weights for region-level analysis: the effect of medicaid expansion on the uninsurance rate among states that did not expand medicaid. *arXiv preprint arXiv:2105.02381*, 2021.

D. Shen, P. Ding, J. Sekhon, and B. Yu. A tale of two panel data regressions. *arXiv preprint arXiv:2207.14481*, 2022.

R. Singh. Kernel ridge riesz representers: Generalization, mis-specification, and the counterfactual effective dimension. *arXiv preprint arXiv:2102.11076v4*, 2024.

R. Singh, L. Xu, and A. Gretton. Kernel methods for causal functions: Dose, heterogeneous, and incremental response curves. *arXiv preprint arXiv:2010.04855*, 2020.

P. Speckman. Minimax estimates of linear functionals in a hilbert space. *Unpublished Manuscript*, 1979.

Z. Tan. Regularized calibrated estimation of propensity scores with model misspecification and high-dimensional data. *Biometrika*, 107(1):137–158, 2020.

M. J. Van Der Laan and D. Rubin. Targeted maximum likelihood learning. *The international journal of biostatistics*, 2(1), 2006.

M. J. Van der Laan, S. Rose, et al. *Targeted learning: causal inference for observational and experimental data*, volume 4. Springer, 2011.

Y. Wang and J. R. Zubizarreta. Minimal dispersion approximately balancing weights: asymptotic properties and practical considerations. *Biometrika*, 107(1):93–105, 2020.

R. K. Wong and K. C. G. Chan. Kernel-based covariate functional balancing for observational studies. *Biometrika*, 105(1):199–213, 2018.

Q. Zhao. Covariate balancing propensity score by tailored loss functions. *The Annals of Statistics*, 47(2): 965–993, 2019.

J. R. Zubizarreta. Stable weights that balance covariates for estimation with incomplete outcome data. *Journal of the American Statistical Association*, 110(511):910–922, 2015.
